# Synthesis of *cis*-Oriented Vicinal Diphenylethylenes through a Lewis Acid-Promoted Annulation of Oxotriphenylhexanoates

**DOI:** 10.1021/acs.joc.1c00445

**Published:** 2021-06-17

**Authors:** Martin Kamlar, Elin Henriksson, Ivana Císařová, Marcus Malo, Henrik Sundén

**Affiliations:** † Chalmers University of Technology, Department of Chemistry and Chemical Engineering, Kemivägen 10, 412 96 Gothenburg, Sweden; ‡ Department of Organic Chemistry, Faculty of Science, Charles University, Hlavova 2030/8, 128 43 Prague 2, Czech Republic; § Department of Inorganic Chemistry, Faculty of Science, Charles University, Hlavova 2030/8, 128 43 Prague 2, Czech Republic; ∥ University of Gothenburg, Department of Chemistry and Molecular Biology, Kemivägen 10, SE-412 96 Gothenburg, Sweden

## Abstract

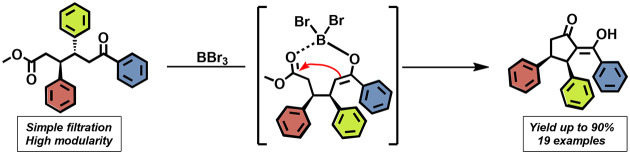

This study explores the synthesis of cyclic *cis*-vicinal phenyl ethylenes from oxotriphenylhexanoates. The reaction is a BBr_3_-promoted cyclization of 1,6-ketoesters (**1**) to five-membered diketo compounds (**2**). The synthesis is interesting as it constitutes one of the few examples of modular stereoselective synthesis of structures with a *cis*-oriented vicinal diphenylethylene. The core structure of **2** can be smoothly derivatized, which makes it a promising synthetic building block for further stereoselective synthetic applications.

## Introduction

**Figure 1:**
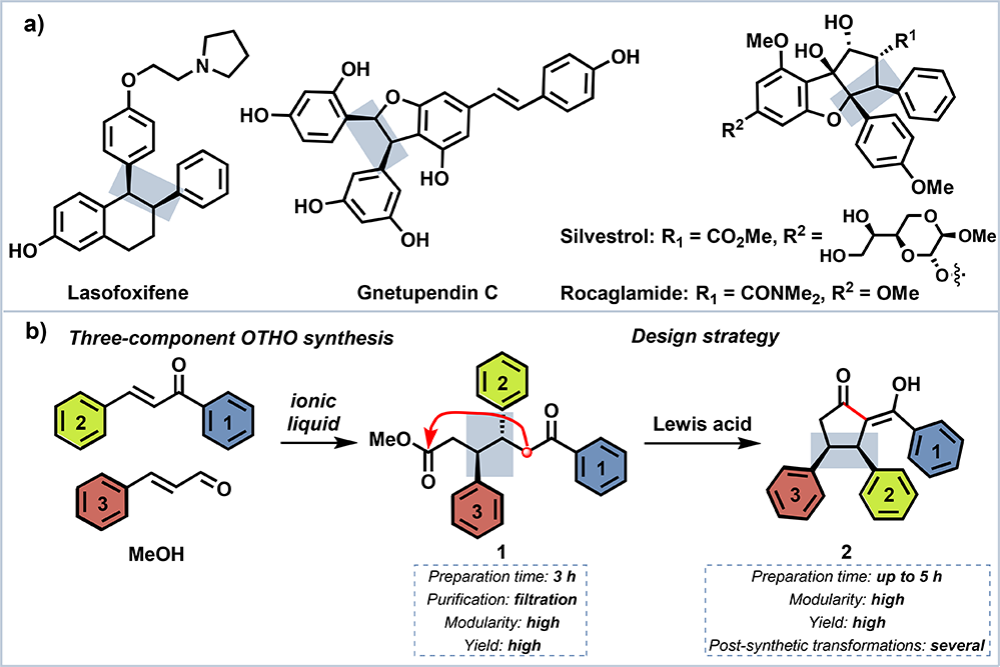
(a) Examples of biologically active compounds with a *cis*-oriented diphenylethylene moiety. (b) Design approach for the synthesis of the desired carbocycle.

Cyclic compounds possessing adjacent C(sp^3^)–aryl moieties are widely present in nature. While in most cases these compounds have a *trans-vic*-diphenylethylene moiety such as indanone^1−7^ and reservatrol-based natural products,^8−19^ the *cis-vic*-diphenylethylene motif has been explored less. Nevertheless, these interesting substances still show biological activity, and representative examples include both pharmaceuticals^20^ and natural products (Figure 1a).^21−34^ For example, the rocaglates, rocaglamide, and silvestrol have received widespread attention as they have been investigated for therapeutic targets such as cancer^21,34,35^ and recently viruses like the coronavirus,^36^ the Ebola virus,^37^ and the hepatitis E virus.^38^ Thus, synthetic strategies leading to the formation the *cis-vic*-diphenylethylene are attractive. However, there are only a limited number of general methods for direct stereoselective formation of cyclic compounds possessing the *cis-vic*-diphenylethylene moiety. It is also worth noting that a majority of these syntheses are devoted to the diphenyl dihydrobenzofuran moiety found in the rocaglates.^21,23,24,34,39−51^

A practical way to prepare the *cis-vic*-configured C(sp^3^)–aryl could potentially be a ring closure of an acyclic starting material with a preset configuration on the carbons bearing the aryl substituents. We envisioned that a Dieckmann type condensation of a 1,6-ketoester such as the oxotriphenylhexanoate (OTHO)^52−56^ would be ideal for this type of transformation. The OTHO has an enolizable ketone and an ester moiety and can, after cyclization, generate a five-membered diketo compound. Furthermore, one very interesting feature of the OTHO is that the synthesis of the OTHO proceeds with exclusive formation of the *trans*-*vic*-diphenylethylene. Used as a starting material in the Dieckmann condensation would thus ensure total control of the *cis*-*vic*-diphenylethylene buildup (Figure 1b).

## Results and Discussion

Our studies commenced with screening of different Lewis acids for the cyclization of **1a** (Table 1) because Lewis acids were already reported to be useful promoters in the Dieckmann type cyclization.^57^ Initial attempts showed that the cyclization reaction was highly dependent on the Lewis acid used. Only BBr_3_ and BCl_3_ delivered the corresponding cyclized product **2a** in good yield after 24 and 48 h, respectively (Table 1, entries 2 and 3, respectively). Single-crystal X-ray analysis of compound **2a** confirmed that the vicinal aromatic rings were in the *cis* orientation (Table 2, entry 2a). Other Lewis acids failed even after prolonged reaction times (Table 1, entries 1 and 4–9). Generally basic conditions were not efficient in promoting the cyclization; only NaH in THF as a solvent successfully afforded **2a** in 45% yield (Table1, entry 10). Investigating the loading of BBr_3_ showed that 1.5 equiv of BBr_3_ is sufficient to reach full conversion within 3 h and produce a yield of 80% (Table 1, entry 11). Higher loadings of BBr_3_ (2–3 equiv) resulted in a significant decrease in the level of formation of **2a**, and the polycyclization reaction of **1a** afforded tricyclic compound **3** (Table 1, entries 12 and 13).^58^ At substoichiometric loadings of BBr_3_, the cyclization reaction does not proceed efficiently. For instance, in the presence of 0.25 equiv of BBr_3_, the reaction reaches 25% conversion and stops (Table 1, entry 14). The reaction performs best in chlorinated solvents such as dichloromethane and chloroform; additionally, toluene is shown to promote the reaction efficiently (Table 1, entries 11, 15, and 16).

**Table 1:**
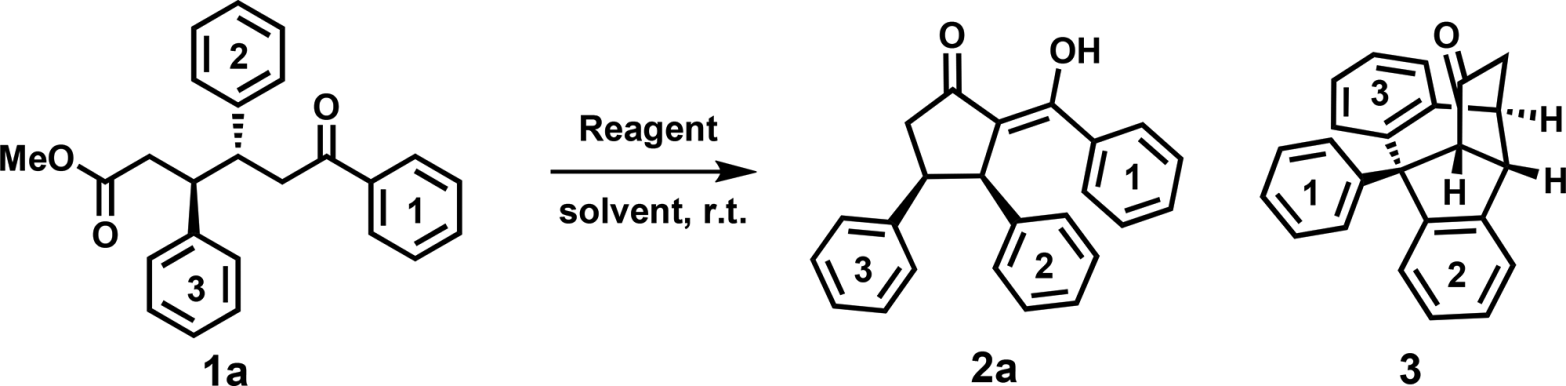
Conditions for the Intramolecular Cyclization of **1a**^a^

entry	reagent	solvent	time (h)	conversion (%)	yield of **2a**/**3** (%)^b^
1	BF_3_·OEt_2_ (1.0 equiv)	DCM	168	0	0/0
2	BBr_3_ (1.0 equiv)	DCM	24	100	67/0
3	BCl_3_ (1.0 equiv)	DCM	48	100	60/0
4	AgOAc (1.0 equiv)	DCM	168	0	–
5	SnCl_4_ (1.0 equiv)	DCM	168	0	–
6	TiCl_4_ (1.0 equiv)	DCM	168	0	–
7	AlCl_3_ (4.0 equiv)	DCM	168	0	–
8	FeCl_3_ (4.0 equiv)	DCM	168	0	–
9	(C_6_F_5_)_3_B (1.0 equiv)	DCM	168	0	–
10	NaH (1.5 equiv)	THF	6	50	45
11	BBr_3_ (1.5 equiv)	DCM	3	100	80/0
12	BBr_3_ (2.0 equiv)	DCM	5	100	30/21
13	BBr_3_ (3.0 equiv)	DCM	5	100	11/28
14	BBr_3_ (0.25 equiv)	DCM	168	25	15/0
15	BBr_3_ (1.5 equiv)	CHCl_3_	20	100	50/0
16	BBr_3_ (1.5 equiv)	toluene	16	100	63/0

a Reactions were carried out on a 0.25 mmol scale of **1a** in DCM (3 mL) in a sealed reaction vessel at room temperature.

b Isolated yield.

**Table 2:**
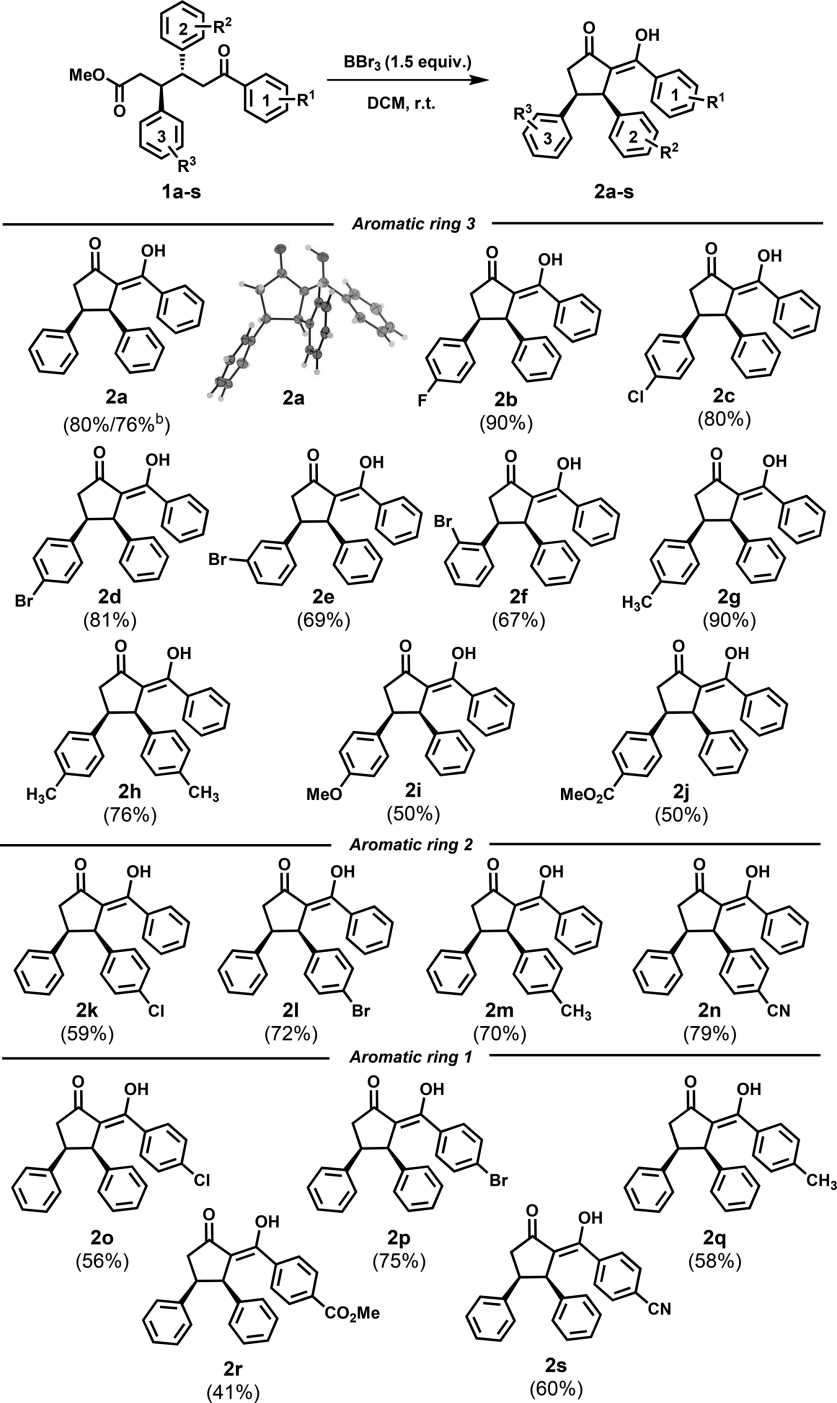
Substrates in BBr_3_-Promoted Intramolecular Cyclization of **1a–s**^a^

a Reactions were carried out on a 0.25 mmol scale of **1** in DCM (3 mL) in a sealed reaction vessel at room temperature.

b 1 mmol reaction scale.

With our optimized conditions in hand, we first decided to verify the effectiveness of the reaction on a larger scale. It was shown that the reaction of **1a** on a 1 mmol scale proceeds with only small decrease in the yield and the corresponding compound **2a** could be isolated in 76% yield. Next, we turned our attention to the scope of the reaction (Table 2). The effect of aromatic ring 3 (Ar3) on the course of cyclization was investigated first. This revealed that the reaction tolerates a broad scope of substituents. For example, OTHOs substituted with halogens in the *para* position delivered the corresponding products **2b–d** in good yields (81–90%). Similarly, OTHOs with a sterically demanding bromine in the *meta* and *ortho* positions on Ar3 could also be employed in the cyclization reaction. However, a decrease in the yield could be observed for compounds **2e** and **2f** (69% and 67%, respectively) compared to that *para*-substituted derivative **2d** (81%). Substrates with an electron rich Ar3 are tolerated by the reaction, as well. For instance, OTHO with a methyl group at the *para* position on Ar3 provides the corresponding product **2g** in high yield (90%). A slight decrease in the yield was observed when OTHO substituted with methyl groups at both Ar2 and Ar3 was used. Corresponding derivative **2h** was isolated in a good yield (76%). Introduction of a methoxy group at the *para* position of Ar3 decreased the yield of the corresponding product **2i** to 50%. The lower yield can be explained by the formation of a demethylated OTHO (**1i′**) as a side product in 25% yield. The influence of a strong electron-withdrawing group was also verified, and BBr_3_ applied on OTHO substituted with a methyl ester group at the *para* position of Ar3 led to the formation of product **2j** in 50% yield. Next, we investigated the effect of aromatic ring 2 (Ar2) in the cyclization reaction. The results showed lower reactivity compared to that of OTHOs substituted at Ar3. This was demonstrated on OTHOs substituted with chlorine or bromine at the *para* position on Ar2 where the corresponding diketones **2k** and **2l** could be isolated in 59% and 72% yields, respectively. OTHO substituted with a methyl at the *para* position of Ar2 led to the formation of **2m**; however, the isolated yield was lower (70%) than that of derivative **2g** (90%). Also, the effect of the electron-withdrawing cyano group at the *para* position of OTHO on Ar2 was successfully investigated, isolating the corresponding product **2n** in 79% yield. Finally, substituent effects at Ar1 of the OTHOs were investigated. Both *p*-chloro and *p*-bromo are well tolerated, and the corresponding diketones **2o** and **2p** were isolated in good yields (56–75%). A comparable result was obtained with OTHO substituted with a methyl group yielding derivative **2q** in 58% yield. In addition, OTHOs substituted with electron-withdrawing methyl ester and cyano groups at the *para* position on Ar1 were successfully employed in the cyclization reaction; however, the yields of corresponding compounds **2r** and **2s** were lower (41% and 60%, respectively) than those of derivatives **2j** and **2n**. Notably, the ester group and the cyano group can be readily transferred to carboxylic acid and amide or amine, respectively, and thus, these substrates open up for the preparation of potentially biologically active compounds with hydrogen bond donor and acceptor capabilities. Expanding the scope by replacing the aryl moieties with an alkyl group is difficult as the synthesis of **1** is not compatible with alkyl substituents.

**Scheme 1:**
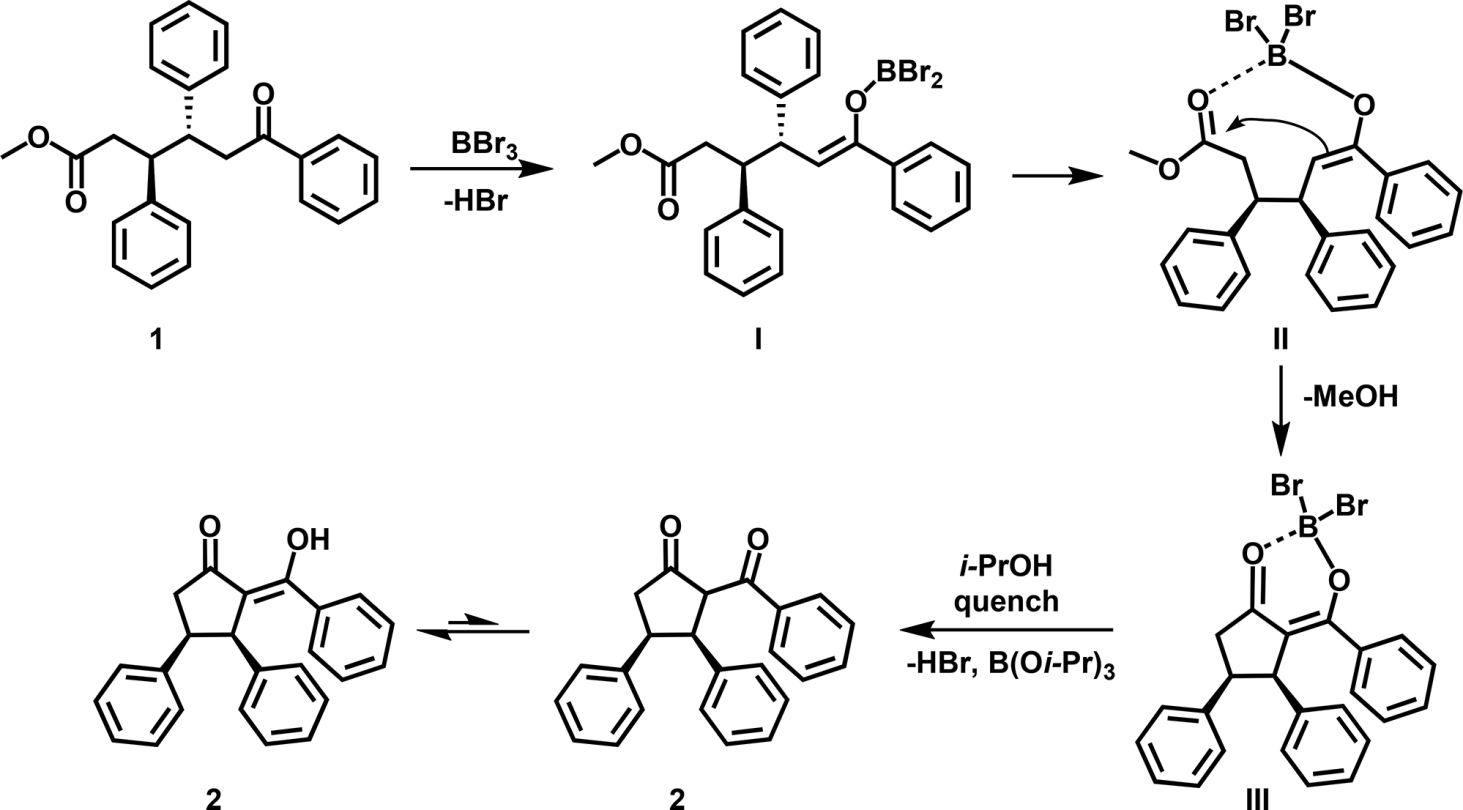
Proposed Mechanism of Intramolecular Cyclization of OTHOs (**1**) to Diketo Compounds **2**

Mechanistically, we propose that the reaction starts with enolization of OTHO **1** promoted by BBr_3_ leading to intermediate **I** (Scheme 1). In the next step, the ester carbonyl is intramolecularly activated by boron leading to the boron-tethered species (**II**). In intermediate **II**, the enol reacts with the ester in an intramolecular fashion, resulting in boron-containing cyclic transition intermediate **III**.^59^ At the end of the reaction, an excess of isopropanol is added to quench intermediate **III**, generating compound **2**, which is stabilized in the enol form.

**Scheme 2:**
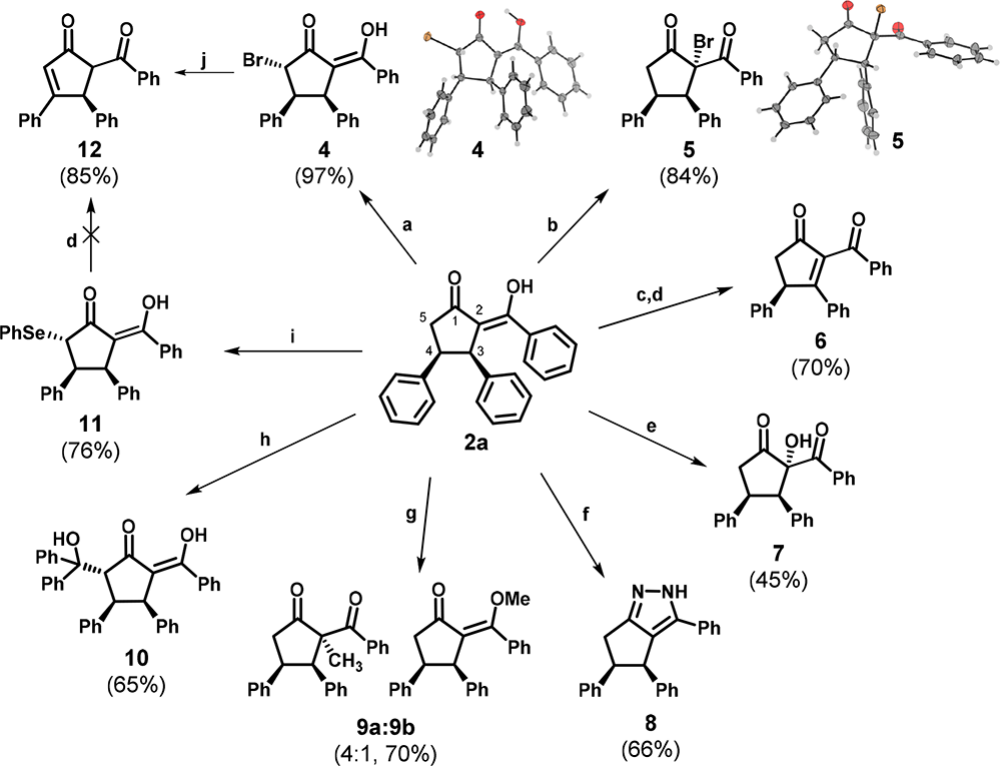
Synthetic Transformation of **2a** into Different Derivatives **4–12** Conditions: (a) pyridinium tribromide, DCM, rt; (b) NBS, DCM, rt; c) *N*-(phenylseleno)phthalimide; DCM, rt; (d) H_2_O_2_, EtAOc, rt; (e) *m*-CPBA, NaHCO_3_, DCM, rt; (f) N_2_H_4_, EtOH, reflux; (g) K_2_CO_3_, MeI, DMF, 60 °C; (h) DIPA, *n*-BuLi, benzophenone, 0 °C to rt; (i) DIPA, *n*-BuLi, *N*-(phenylseleno)phthalimide, DCM, 0 °C to rt; (j) DABCO, THF, rt.

To demonstrate the versatility of this methodology, we used **2a** as a building block in various transformations (Scheme 2). At first, we focused on bromination reactions. When **2a** was subjected to *N*-bromosuccinimide,^60^ α-bromo derivative **5** was isolated in 84% yield as a single diastereoisomer. Interestingly, when **2a** was treated with pyridinium tribromide, δ-brominated regioisomer **4** was isolated in high yield (97%). This bromination strategy offers a route toward compounds that can be used as building blocks in, for example, the Reformatsky type cyanation reaction mediated by samarium salts^61^ or in organocatalytic cyclopropanation reactions.^62^ Remarkably, compound **4** was like derivative **5** isolated as a single diastereoisomer with an *anti* orientation of bromine and both aromatic rings. This relative configuration of substituents on the cyclopentanone ring was confirmed by X-ray diffraction of both derivatives **4** and **5** (see the Supporting Information).

Apart from bromination, we also showed that **2a** can be regioselectively substituted with phenylselenium on either C-2 or C-5 of cyclopentanone based on the used conditions. When **2a** was subjected to *N*-(phenylseleno)phthalimide in the presence of 2 equiv of LDA, prepared in situ, selenylated product **11** was isolated in 76% yield as a single diastereoisomer, whereas selenylation with *N*-(phenylseleno)phthalimide without a base led to the selenylation on C-2 of **2a**. The corresponding intermediate was unstable upon isolation, but after the crude mixture had been treated with hydrogen peroxide, the elimination reaction took place and unsaturated derivative **6** was isolated in 70% overall yield. The same reaction conditions were also used on compound **11**. Unfortunately, elimination did not proceed, and decomposition of the starting material was observed. On the contrary, corresponding unsaturated derivative **12** was isolated in good yield (85%) after treatment of **4** with 1,4-diazabicyclo[2.2.2]octane (DABCO) in THF at room temperature. Furthermore, diastereoselective hydroxylation of **2a** was developed in the presence of *m*-CPBA and hydroxylated diketone **7** could be isolated in 45% yield. Next, the possibility of C–C bond formation on C-2 and C-5 of **2a** was investigated. When **2a** was treated with iodomethane in the presence of potassium carbonate, methylated product **9** was isolated as a single diastereoisomer in a good yield of 70%, however, as a mixture of regioisomers (4:1 keto:enol). On the contrary, when 2 equiv of in situ-generated LDA was used in the presence of **2a** and benzophenone as an electrophile,^63^ the corresponding product **10** was isolated in 65% yield as a single diastereomer. These examples nicely illustrate the influence of both *cis*-oriented aromatic rings on stereoselective addition of electrophiles to both C-2 and C-5 to the unhindered side of the cyclic keto ester. We were also able to show that **2a** can be transformed into pyrazole derivative **8** upon being treated with hydrazine hydrate in ethanol under reflux conditions.^64^

**Figure 2:**
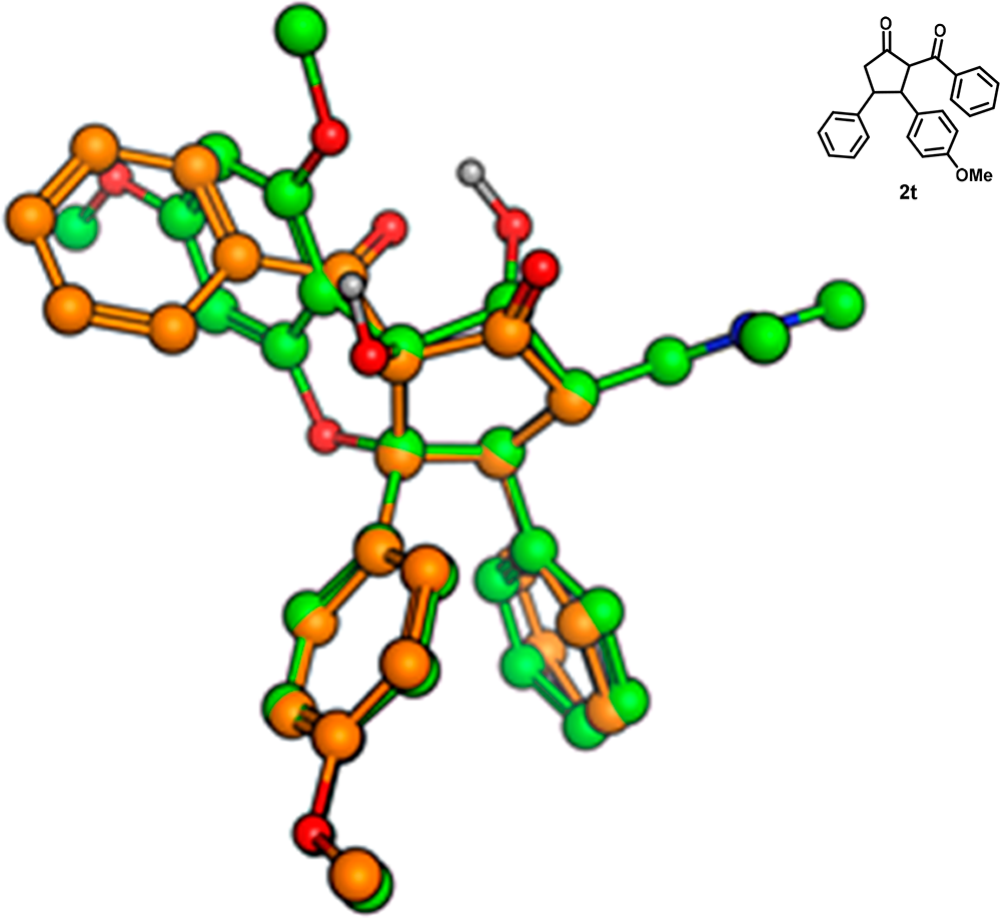
Superimposition of the global minima conformation of rocaglamide (green) and a low-energy conformation (Δ*E* = 0.7 kcal mol^–1^) of **2t** (orange).

To validate whether our diastereoselective synthesis of *cis*-*vic*-diphenylethylene dicarbonyl compounds could be useful for the synthesis of compounds that are structurally similar to rocaglamide or silvestrol, a superimposition was made between compound **2t** and rocaglamide. As it turns out, the superimposition shows an unambiguous match between both *cis*-oriented neighboring Ar of **2t** and the rocaglamide aromatic rings (Figure 2). This provides evidence that the geometry of the diphenylethylene moiety found in the series of compound **2** mimics the diphenylethylene found in natural products such as rocaglamide and silvestrol.

## Conclusion

We have developed an intramolecular cyclization reaction of 1,6-ketoesters using boron tribromide as a Lewis acid promoter. This methodology provides straightforward access to 1,3-diketo compounds with a cyclopentane moiety having a *cis*-oriented vicinal diphenylethylene that is unique and difficult to obtain by known synthetic methods. We have also shown that the defined orientation of both vicinal aromatic rings has a directing effect on stereoselectivity in subsequent substitution reactions with bromine, selenium, or a carbon electrophile on C-2 or C-5 of the 1,3-diketones (Scheme 2). Also, regioselective elimination procedures were developed on the basis of the substitution at C-2 or C-5 providing access to two types of α,β-unsaturated carbonyl compounds. Moreover, successful transformation of a 1,3-diketo compound to a heterocyclic pyrazole derivative has been demonstrated showing the importance of 1,3-diketones as potential building blocks for druglike architectures. Additionally, the matching superimposition of **2t** and rocaglamide indicates that the substructure found in compound **2** is a good starting point for the synthesis of a molecular library for probing biological activity. Compounds including *cis*-*vic*-diphenylethylene-oriented motifs such as rocaglamide and silverstrol have attracted considerable pharmaceutical interest, e.g., in cancer treatment and as antiviral agents.^21,36−38^ The synthesis protocol presented here can easily be fine-tuned to achieve a wanted pharmacological profile and thus opens up new opportunities to further explore new analogues for the therapies mentioned above.

## Experimental Section

### Molecular Modeling

To verify the hypothesis that the aromatic rings in **2t** can mimic the corresponding rings in rocaglamide, molecular mechanics calculations were used. A conformational search was performed on both compounds using the Amber10:EHT force field, the Born aqueous solvation model, and the LowModeMD search methodology. The standard settings were applied, and the energy cutoff was set to 5 kcal/mol from their global minima. An ensemble of conformations of the two compounds (24 of rocaglamide and 14 of **2t**) was achieved, and these were crosswise superimposed (336 combinations). The conclusion was that superimposition of **2t** mimics rocaglamide very well, and among the top-scoring combinations were the global minima of rocaglamide and a low-energy conformation of **2t**, i.e., Δ*E* = 0 and 0.7 kcal mol^–1^ (Figure 2). The superimposition queries were aromatic rings 2 and 3, the methoxy oxygen, and C-3 and C-4 in the five-membered ring (see Scheme 2 for numbering). All molecular calculations were performed using tools implemented in MOE modeling software.^65^

### General Experimental Information

Column chromatography was performed on automated column chromatography Biotage Isolera Spektra One with Biotage SNAP-10g KP-sil columns. Thin layer chromatography (TLC) was performed on Merck TLC plates precoated with silica gel 60 F254 (Art 5715, 0.25 mm) and visualized with ultraviolet light (254 nm). The ^1^H NMR (400 MHz) and ^13^C NMR (101 MHz) spectra were recorded on a Varian 400 instrument, and ^19^F NMR (470 MHz) spectra were recorded on a Varian 500 spectrometer. The chemical shifts are reported in parts per million (δ) relative to the residual solvent peak CDCl_3_: ^1^H NMR at δ 7.26 and ^13^C NMR at δ 77.16. Coupling constants (*J*) are reported in hertz. Infrared (IR) spectra were recorded on a PerkinElmer series FT-IR spectrometer and are reported in wavenumbers (cm^–1^). Melting points were recorded on a Stuart Scientific Melting Point SMP1 instrument. Gas chromatographic studies were performed using an Agilent 7820A instrument equipped with a flame ionization detector and an Agilent HP-5 19091J-413 column. Crystallographic data were obtained using a Bruker D8 VENTURE Kappa Duo PHOTONIII instrument. The 1-ethyl-3-methylimidazolium acetate (EMIMAc) was purchased from Sigma-Aldrich (Stockholm, Sweden), produced by BASF ≥95%, and dried in vacuo with heating prior to use. All other solvents and reagents were purchased from commercial sources and used without further treatments.

#### Synthesis of the Starting Material, OTHOs (**1**)

OTHOs **1a–d**, **1g**, **1i–l**, **1n**, **1o**, **1e**, **1f**, **1h**, **1m**, and **1p–s** were synthesized according to previously published procedures.^52,58^

#### Methyl 4-(4-Cyanophenyl)-6-oxo-3,4-diphenylhexanoate (**1n**)

Isolated as a white solid (450 mg, 53% yield); mp 167–169 °C (from MeOH); ^1^H NMR (400 MHz, CDCl_3_) δ 7.67–7.57 (m, 4H), 7.52–7.42 (m, 3H), 7.39–7.22 (m, 7H), 3.70 (td, *J* = 10.8 Hz, *J*′ = 3.2 Hz, 1H), 3.48–3.39 (s, 4H), 3.28 (dd, *J* = 17.2 Hz, *J*′ = 10.5 Hz, 1H), 2.96 (dd, *J* = 17.3 Hz, *J*′ = 3.2 Hz, 1H), 2.50 (dd, *J* = 15.6 Hz, *J*′ = 10.2 Hz, 1H), 2.34 (dd, *J* = 15.6 Hz, *J*′ = 4.5 Hz, 1H); ^13^C{^1^H} NMR (101 MHz, CDCl_3_) δ 197.8, 172.2, 148.4, 141.3, 136.7, 133.3, 132.6 (2C), 129.4 (2C), 129.1 (2C), 128.7 (2C), 128.2 (2C), 127.9 (2C), 127.6, 118.9, 111.0, 51.7, 47.4, 46.7, 43.4, 39.8; ATR-FTIR ν 2951, 2228, 1726, 1718, 1679, 1609, 1597, 1436, 1237, 1157 cm^–1^; HRMS (ESI) *m*/*z* calcd for C_26_H_24_NO_3_ [M + H]^+^ 398.1751, found 398.1751.

#### General Procedure for the BBr_3_-Promoted Synthesis of **2**

To a flask equipped with a magnetic stir bar were added OTHO **1a–s** (0.25 mmol, 1 equiv) and DCM (3 mL). The solution was stirred at room temperature under nitrogen and syringed with a solution of BBr_3_ (375 μL, 1 M in DCM, 1.5 equiv). The mixture was stirred until full conversion was reached (monitored by TLC), then the reaction quenched with *i*-PrOH (2 mL), and the mixture evaporated to dryness. The crude material was separated by flash chromatography with a petroleum ether/EtOAc mixture, yielding the corresponding compound **2a–s**.

#### Procedure for the BBr_3_-Promoted Synthesis of **2a** on a 1 mmol Scale

To a flask equipped with a magnetic stir bar were added OTHO **1a** (0.372 mmol, 1 equiv) and DCM (12 mL). The solution was stirred at room temperature under nitrogen and syringed with a solution of BBr_3_ (1.5 mL, 1 M in DCM, 1.5 equiv). The mixture was stirred until full conversion was reached (monitored by TLC), then the reaction quenched with *i*-PrOH (8 mL), and the mixture evaporated to dryness. The crude material was separated by flash chromatography in a petroleum ether/EtOAc mixture yielding corresponding compound **2a** as a yellow solid (204 mg, 76%).

#### (2*Z*)-2-[Hydroxy(phenyl)methylidene]-3,4-diphenylcyclopentan-1-one (**2a**)

Purified in 92:8 petroleum ether/EtOAc; 68 mg yield as a yellow solid (80%); mp 121–123 °C (from MeOH); 1:10 keto:enol. Enol: ^1^H NMR (400 MHz, CDCl_3_) δ 7.55–7.49 (m, 2H), 7.40–7.34 (m, 1H), 7.28–7.24 (m, 3H), 7.12–7.06 (m, 6H), 6.78–6.74 (m, 3H), 4.36 (d, *J* = 7.4 Hz, 1H), 3.87 (dt, *J* = 14.0 Hz, *J*′ = 7.2 Hz, 1H), 3.09 (dd, *J* = 17.4 Hz, *J*′ = 13.5 Hz, 1H), 2.62 (dd, *J* = 17.3 Hz, *J*′ = 7.1 Hz, 1H); ^13^C{^1^H} NMR (101 MHz, CDCl_3_) δ 209.3, 170.8, 140.5, 138.9, 133.9, 131.2, 128.6 (2C), 128.4 (2C), 128.3 (2C), 128.2 (4C), 127.9 (2C), 126.8, 126.7, 113.3, 52.0, 48.3, 39.8. Keto: ^1^H NMR (400 MHz, CDCl_3_) δ 8.04–7.96 (m, 2H), 7.62–7.56 (m, 1H), 7.50–7.48 (m, 2H), 7.17–7.13 (m, 3H), 7.11–7.06 (m, 3H), 6.82–6.76 (m, 4H), 4.83 (d, *J* = 8.3 Hz, 1H), 4.57–4.44 (m, 1H), 4.07 (q, *J* = 7.0 Hz, 1H), 3.01–2.92 (m, 2H); ^13^C{^1^H} NMR (101 MHz, CDCl_3_) δ 212.3, 194.5, 139.6, 138.6, 136.6, 133.7, 129.6, 128.7 (2C), 128.3 (2C), 128.2 (3C), 128.1 (4C), 127.0, 126.9, 61.2, 51.1, 45.5, 44.8; ATR-FTIR ν 1758, 1642, 1608, 1593, 1570, 1357, 1268, 1222, 1146, 1099 cm^–1^; HRMS (ESI) *m*/*z* calcd for C_24_H_21_O_2_ [M + H]^+^ 341.1537, found 341.1536.

#### (2*Z*)-4-(4-Fluorophenyl)-2-[hydroxy(phenyl)methylidene]-3-phenylcyclopentan-1-one (**2b**)

Purified in 99:1 to 97:3 petroleum ether/EtOAc; 81 mg yield as a thick orange oil (90%); 1:10 keto:enol. Enol: ^1^H NMR (400 MHz, CDCl_3_) δ 7.55–7.51 (m, 2H), 7.41–7.34 (m, 1H), 7.26 (s, 2H), 7.17–7.09 (m, 3H), 6.83–6.66 (m, 6H), 4.33 (d, *J* = 7.4 Hz, 1H), 3.85 (dt, *J* = 14.0 Hz, *J*′ = 7.2 Hz, 1H), 3.02 (dd, *J* = 17.3 Hz, *J*′ = 13.5 Hz, 1H), 2.62 (dd, *J* = 17.3 Hz, *J*′ = 7.1 Hz, 1H); ^13^C{^1^H} NMR (101 MHz, CDCl_3_) δ 208.9, 171.1, 161.7 (d, *J* = 245.2 Hz), 140.4, 134.7 (d, *J* = 3.1 Hz), 133.9, 131.3, 129.6 (d, *J* = 8.0 Hz, 2C), 128.6 (2C), 128.4 (6C), 126.9, 114.8 (d, *J* = 21.2 Hz, 2C), 113.1, 52.0, 47.6, 40.1; ^19^F NMR (470 MHz, CDCl_3_) δ −115.9 (ddd, *J* = 13.7 Hz, *J*′ = 8.3 Hz, *J*″ = 5.4 Hz). Keto: ^1^H NMR (400 MHz, CDCl_3_) δ 8.07–7.96 (m, 2H), 7.62–7.57 (m, 1H), 7.50–7.45 (m, 2H), 7.28–7.24 (m, 1H), 7.17–7.09 (m, 2H), 6.88–6.74 (m, 6H), 4.82 (d, *J* = 7.7 Hz, 1H), 4.46 (t, *J* = 7.3 Hz, 1H), 4.08 (q, *J* = 7.2 Hz, 1H), 2.93 (d, *J* = 7.8 Hz, 2H); ^13^C{^1^H} NMR (101 MHz, CDCl_3_) δ 212.0, 194.4, 161.8 (d, *J* = 245.6 Hz), 138.6, 136.4, 135.3 (d, *J* = 3.3 Hz), 133.9, 133.9, 129.7 (2C), 129.5 (d, *J* = 8.0 Hz, 2C), 128.8 (2C), 128.2 (2C), 127.2, 115.1 (d, *J* = 21.2 Hz, 2C), 61.4, 51.1, 44.9, 44.6; ^19^F NMR (470 MHz, CDCl_3_) δ −115.8 (tt, *J* = 8.5 Hz, *J*′ = 5.5 Hz); ATR-FTIR ν 2905, 1746, 1636, 1592, 1565, 1510, 1492, 1448, 1352, 1219, 1150, 1096 cm^–1^; HRMS (ESI) *m*/*z* calcd for C_24_H_20_FO_2_ [M + H]^+^ 359.1442, found 359.1437.

#### (2*Z*)-4-(4-Chlorophenyl)-2-[hydroxy(phenyl)methylidene]-3-phenylcyclopentan-1-one (**2c**)

Purified in 98:2 to 97:3 petroleum ether/EtOAc; 74 mg yield as a thick orange oil (80%); 1:10 keto:enol. Enol: ^1^H NMR (400 MHz, CDCl_3_) δ 7.57–7.47 (m, 2H), 7.43–7.34 (m, 1H), 7.26 (s, 2H), 7.18–7.11 (m, 3H), 7.09–7.02 (m, 2H), 6.82–6.76 (m, 2H), 6.67 (d, *J* = 8.4 Hz, 2H), 4.34 (d, *J* = 7.4 Hz, 1H), 3.83 (dt, *J* = 14.0 Hz, *J*′ = 7.2 Hz, 1H), 3.03 (dd, *J* = 17.3 Hz, *J*′ = 13.4 Hz, 1H), 2.61 (dd, *J* = 17.3 Hz, *J*′ = 7.1 Hz, 1H); ^13^C{^1^H} NMR (101 MHz, CDCl_3_) δ 208.7, 171.1, 140.3, 137.6, 133.9, 132.5, 131.4, 129.5 (2C), 128.6 (2C), 128.5 (2C), 128.4 (4C), 128.1 (2C), 127.0, 113.1, 51.8, 47.7, 39.9. Keto: ^1^H NMR (400 MHz, CDCl_3_) δ 8.02 (dd, *J* = 8.4 Hz, *J*′ = 1.3 Hz, 2H), 7.64–7.55 (m, 1H), 7.50–7.46 (m, 2H), 7.28–7.24 (m, 3H), 7.16–7.10 (m, 4H), 6.82–6.76 (m, 2H), 6.76–6.71 (m, 2H), 4.82 (d, *J* = 7.6 Hz, 1H), 4.47 (t, *J* = 7.3 Hz, 1H), 4.08 (q, *J* = 7.3 Hz, 1H), 2.92 (d, *J* = 7.6 Hz, 2H); ^13^C{^1^H} NMR (101 MHz, CDCl_3_) δ 211.8, 194.3, 138.4, 138.1, 136.4, 132.7, 131.4, 129.7 (2C), 129.4 (2C), 128.8 (2C), 128.5 (2C), 128.3 (2C), 128.2 (2C), 127.3, 61.5, 51.0, 45.0, 44.4; ATR-FTIR ν 3026, 1743, 1595, 1570, 1493, 1447, 1362, 1266, 1226, 1091, 1016 cm^–1^; HRMS (ESI) *m*/*z* calcd for C_24_H_20_ClO_2_ [M + H]^+^ 375.1146, found 375.1160.

#### (2*Z*)-4-(4-Bromophenyl)-2-[hydroxy(phenyl)methylidene]-3-phenylcyclopentan-1-one (**2d**)

Purified in 98:2 to 97:3 petroleum ether/EtOAc; 85 mg yield as a thick orange oil (81%); 1:10 keto:enol. Enol: ^1^H NMR (400 MHz, CDCl_3_) δ 7.55–7.49 (m, 2H), 7.42–7.35 (m, 1H), 7.29–7.19 (m, 4H), 7.17–7.10 (m, 3H), 6.81–6.77 (m, 2H), 6.65–6.57 (m, 2H), 4.35 (d, *J* = 7.4 Hz, 1H), 3.81 (dt, *J* = 14.0 Hz, *J*′ = 7.2 Hz, 1H), 3.02 (dd, *J* = 17.3 Hz, *J*′ = 13.5 Hz, 1H), 2.61 (dd, *J* = 17.3 Hz, *J*′ = 7.1 Hz, 1H); ^13^C{^1^H} NMR (101 MHz, CDCl_3_) δ 208.7, 171.1, 140.2, 138.1, 133.9, 131.3, 131.0 (2C), 129.9 (2C), 128.6 (2C), 128.5 (2C), 128.4 (4C), 127.0, 120.7, 113.1, 51.8, 47.8, 39.9. Keto: ^1^H NMR (400 MHz, CDCl_3_) δ 8.07–7.99 (m, 2H), 7.62–7.57 (m, 1H), 7.50–7.46 (m, 1H), 7.26 (s, 1H), 7.17–7.09 (m, 4H), 6.82–6.75 (m, 2H), 6.68 (d, *J* = 8.4 Hz, 2H), 4.82 (d, *J* = 7.6 Hz, 1H), 4.47 (t, *J* = 7.3 Hz, 1H), 4.06 (q, *J* = 7.3 Hz, 1H), 2.95–2.89 (m, 2H); ^13^C{^1^H} NMR (101 MHz, CDCl_3_) δ 211.7, 194.2, 138.6, 138.4, 136.4, 133.9, 131.3 (2C), 129.7 (4C), 128.8 (2C), 128.5 (2C), 128.1 (2C), 127.3, 120.8, 61.4, 50.9, 45.1, 44.3; ATR-FTIR ν 3028, 1746, 1596, 1567, 1490, 1365, 1263, 1225, 1077, 1011 cm^–1^; HRMS (ESI) *m*/*z* calcd for C_24_H_20_BrO_2_ [M + H]^+^ 419.0641, found 419.0635.

#### (2*Z*)-4-(3-Bromophenyl)-2-[hydroxy(phenyl)methylidene]-3-phenylcyclopentan-1-one (**2e**)

Purified in 97:3 to 95:5 petroleum ether/EtOAc; 72 mg yield as a thick orange oil (69%); 1:10 keto:enol. Enol: ^1^H NMR (400 MHz, CDCl_3_) δ 7.56–7.51 (m, 2H), 7.42–7.35 (m, 1H), 7.30–7.22 (m, 3H), 7.16–7.13 (m, 3H), 6.96–6.89 (m, 2H), 6.81–6.77 (m, 2H), 6.67–6.63 (m, 1H), 4.35 (d, *J* = 7.4 Hz, 1H), 3.81 (dt, *J* = 14.1 Hz, *J*′ = 7.3 Hz, 1H), 3.03 (dd, *J* = 17.3 Hz, *J*′ = 13.4 Hz, 1H), 2.62 (dd, *J* = 17.3 Hz, *J*′ = 7.2 Hz, 1H); ^13^C{^1^H} NMR (101 MHz, CDCl_3_) δ 208.5, 171.2, 141.5, 140.2, 133.8, 131.5, 131.4, 129.9, 129.5, 128.5 (2C), 128.4 (6C), 127.1, 126.7, 122.1, 113.0, 51.9, 48.0, 39.7. Keto: ^1^H NMR (400 MHz, CDCl_3_) δ 8.06–8.00 (m, 2H), 7.62–7.57 (m, 1H), 7.51–7.47 (m, 1H), 7.29–7.22 (m, 3H), 7.16–7.13 (m, 3H), 7.03–6.99 (m, 1H), 6.83–6.77 (m, 2H), 6.76–6.72 (m, 1H), 4.82 (d, *J* = 7.1 Hz, 1H), 4.46 (t, *J* = 7.1 Hz, 1H), 4.08 (q, *J* = 7.4 Hz, 1H), 2.92 (dd, *J* = 7.8 Hz, *J*′ = 1.1 Hz, 1H); ^13^C{^1^H} NMR (101 MHz, CDCl_3_) δ 211.6, 194.2, 141.9, 138.4, 136.3, 133.9, 131.3, 130.0, 129.7 (3C), 128.8 (2C), 128.4 (2C), 128.1 (2C), 127.4, 126.5, 122.4, 61.7, 51.0, 45.3, 43.9; ATR-FTIR ν 3024, 2917, 1744, 1637, 1592, 1569, 1494, 1475, 1357, 1265, 1225, 1152, 1098, 1075 cm^–1^; HRMS (ESI) *m*/*z* calcd for C_24_H_20_BrO_2_ [M + H]^+^ 419.0641, found 419.0631.

#### (2*Z*)-4-(2-Bromophenyl)-2-[hydroxy(phenyl)methylidene]-3-phenylcyclopentan-1-one (**2f**)

Purified in 97:3 to 95:5 petroleum ether/EtOAc; 70 mg yield as a thick orange oil (67%); 1:24 keto:enol; ^1^H NMR (400 MHz, CDCl_3_) δ 7.62–7.49 (m, 3H), 7.42–7.34 (m, 1H), 7.30–7.26 (m, 2H), 7.12–7.04 (m, 3H), 6.99–6.94 (m, 1H), 6.92–6.79 (m, 3H), 6.41 (dd, *J* = 7.9 Hz, *J*′ = 1.7 Hz, 1H), 4.73 (d, *J* = 7.4 Hz, 1H), 4.29 (dt, *J* = 14.1 Hz, *J*′ = 7.2 Hz, 1H), 3.13 (dd, *J* = 17.2 Hz, *J*′ = 13.7 Hz, 1H), 2.59 (dd, *J* = 17.2 Hz, *J*′ = 7.1 Hz, 1H); ^13^C{^1^H} NMR (101 MHz, CDCl_3_) δ 208.4, 171.5, 140.8, 138.0, 133.9, 132.7, 131.4, 128.5 (2C), 128.4 (5C), 128.3, 128.0 (2C), 126.9, 126.8, 125.5, 112.6, 48.4, 47.1, 39.1; ATR-FTIR ν 3067, 3024, 1596, 1567, 1494, 1471, 1367, 1230, 1023 cm^–1^; HRMS (ESI) *m*/*z* calcd for C_24_H_20_BrO_2_ [M + H]^+^ 419.0641, found 419.0630.

#### (2*Z*)-2-[Hydroxy(phenyl)methylidene]-4-(4-methylphenyl)-3-phenylcyclopentan-1-one (**2g**)

Purified in 98:2 to 97:3 petroleum ether/EtOAc; 80 mg yield as a thick yellow oil (90%); 1:7 keto:enol. Enol: ^1^H NMR (400 MHz, CDCl_3_) δ 7.57–7.54 (m, 2H), 7.40–7.35 (m, 1H), 7.29–7.26 (m, 2H), 7.14–7.11 (m, 3H), 6.93–6.91 (m, 2H), 6.85–6.78 (m, 2H), 6.67–6.65 (m, 2H), 4.36 (d, *J* = 7.4 Hz, 1H), 3.84 (dt, *J* = 14.1 Hz, *J*′ = 7.2 Hz, 1H), 3.08 (dd, *J* = 17.3 Hz, *J*′ = 13.6 Hz, 1H), 2.61 (dd, *J* = 17.3 Hz, *J*′ = 7.1 Hz, 1H), 2.27 (s, 3H); ^13^C{^1^H} NMR (101 MHz, CDCl_3_) δ 209.5, 170.7, 140.6, 136.3, 135.8, 134.0, 131.2, 128.7 (2C), 128.6 (2C), 128.4 (2C), 128.3 (2C), 128.2 (2C), 128.1 (2C), 126.7, 113.4, 51.9, 48.1, 40.1, 21.1. Keto: ^1^H NMR (400 MHz, CDCl_3_) δ 8.05–8.00 (m, 2H), 7.52–7.48 (m, 2H), 7.30–7.26 (m, 2H), 7.15–7.08 (m, 3H), 7.00–6.96 (m, 2H), 6.84–6.78 (m, 1H), 6.72 (d, *J* = 8.1 Hz, 2H), 4.86 (d, *J* = 8.6 Hz, 1H), 4.51 (dd, *J* = 8.6 Hz, *J*′ = 6.8 Hz, 1H), 4.05 (q, *J* = 6.9 Hz, 1H), 2.96 (d, *J* = 6.9 Hz, 1H), 2.27 (s, 3H); ^13^C{^1^H} NMR (101 MHz, CDCl_3_) δ 212.5, 194.6, 172.1, 142.2, 138.7, 136.4, 134.0, 133.7, 129.6, 128.9 (2C), 128.7 (2C), 128.6 (2C), 128.2 (2C), 128.0 (2C), 127.9 (2C), 61.1, 51.9, 45.1, 22.0; ATR-FTIR ν 3024, 1744, 1608, 1592, 1571, 1515, 1492, 1448, 1365, 1261, 1227, 1150, 1098, 1078, 1030 cm^–1^; HRMS (ESI) *m*/*z* calcd for C_25_H_23_O_2_ [M + H]^+^ 355.1693, found 355.1677.

#### (2*Z*)-2-[Hydroxy(phenyl)methylidene]-3,4-bis(4-methylphenyl)cyclopentan-1-one (**2h**)

Purified in 98:2 petroleum ether/EtOAc; 70 mg yield as a thick yellow oil (76%); 1:5 keto:enol. Enol: ^1^H NMR (400 MHz, CDCl_3_) δ 7.58–7.51 (m, 2H), 7.40–7.33 (m, 1H), 7.28–7.25 (m, 2H), 6.91 (d, *J* = 7.8 Hz, 4H), 6.71–6.61 (m, 4H), 4.29 (d, *J* = 7.2 Hz, 1H), 3.79 (dt, *J* = 14.0 Hz, *J*′ = 7.1 Hz, 1H), 3.04 (dd, *J* = 17.3 Hz, *J*′ = 13.7 Hz, 1H), 2.57 (dd, *J* = 17.3 Hz, *J*′ = 7.0 Hz, 1H), 2.26 (s, 6H); ^13^C{^1^H} NMR (101 MHz, CDCl_3_) δ 209.7, 170.4, 137.4, 136.3 (2C), 136.2, 136.0, 131.2, 129.0 (2C), 128.6 (2C), 128.6 (2C), 128.5 (2C), 128.3 (2C), 128.2 (2C), 113.8, 51.5, 48.2, 40.1, 21.2 (2C). Keto: ^1^H NMR (400 MHz, CDCl_3_) δ 8.02–7.96 (m, 2H), 7.59–7.55 (m, 1H), 7.51–7.42 (m, 3H), 7.29–7.24 (m, 2H), 6.97 (d, *J* = 7.8 Hz, 2H), 6.74–6.68 (m, 3H), 4.79 (d, *J* = 8.5 Hz, 1H), 4.43 (dd, *J* = 8.5 Hz, *J*′ = 6.8 Hz, 1H), 4.00 (q, *J* = 6.9 Hz, 1H), 2.93 (d, *J* = 6.9 Hz, 2H), 2.26 (s, 6H); ^13^C{^1^H} NMR (101 MHz, CDCl_3_) δ 212.8, 194.8, 136.7, 136.6, 136.5, 136.4, 135.6, 133.7, 129.6 (2C), 128.9 (4C), 128.7 (2C), 128.1 (4C), 61.4, 50.8, 45.2, 45.1, 21.1 (2C); ATR-FTIR ν 3020, 2913, 1744, 1573, 1513, 1494, 1361, 1223 cm^–1^; HRMS (ESI) *m*/*z* calcd for C_26_H_25_O_2_ [M + H]^+^ 369.1849, found 369.1862.

#### (2*Z*)-2-[Hydroxy(phenyl)methylidene]-4-(4-methoxyphenyl)-3-phenylcyclopentan-1-one (**2i**)

Purified in 98:2 petroleum ether/EtOAc; 46 mg yield as a thick yellow oil (50%); 1:5 keto:enol. Enol: ^1^H NMR (400 MHz, CDCl_3_) δ 7.61–7.51 (m, 2H), 7.39–7.33 (m, 1H), 7.28–7.24 (m, 2H), 7.15–7.06 (m, 3H), 6.82–6.75 (m, 2H), 6.65–6.63 (m, 3H), 4.32 (d, *J* = 7.4 Hz, 1H), 3.82 (dt, *J* = 14.0 Hz, *J*′ = 7.2 Hz, 1H), 3.73 (s, 3H), 3.02 (dd, *J* = 17.3 Hz, *J*′ = 13.6 Hz, 1H), 2.59 (dd, *J* = 17.3 Hz, *J*′ = 7.1 Hz, 1H); ^13^C{^1^H} NMR (101 MHz, CDCl_3_) δ 209.5, 170.7, 158.4, 140.7, 134.0, 131.2, 129.6, 129.2 (2C), 128.7 (2C), 128.4 (2C), 128.3 (4C), 126.7, 113.6, 113.4 (2C), 55.3, 52.0, 47.7, 40.2. Keto: ^1^H NMR (400 MHz, CDCl_3_) δ 8.04–7.96 (m, 2H), 7.61–7.56 (m, 1H), 7.50–7.45 (m, 2H), 7.14–7.09 (m, 3H), 6.82–6.75 (m, 2H), 6.70 (d, *J* = 2.8 Hz, 2H), 6.64 (d, *J* = 3.2 Hz, 2H), 4.81 (d, *J* = 8.5 Hz, 0H), 4.46 (dd, *J* = 8.4, 6.8 Hz, 1H), 4.02 (q, *J* = 6.9 Hz, 1H), 3.75 (s, 2H), 2.96–2.90 (m, 1H); ^13^C{^1^H} NMR (101 MHz, CDCl_3_) δ 212.6, 195.0, 138.8, 136.6, 133.8, 131.6, 131.0 (2C), 129.6, 129.1 (2C), 128.8 (2C), 128.3 (4C), 127.0, 113.4 (2C), 61.2, 56.3, 51.2, 45.1, 44.8; ATR-FTIR ν 2840, 1744, 1610, 1515, 1365, 1248, 1180, 1032 cm^–1^; HRMS (ESI) *m*/*z* calcd for C_25_H_23_O_3_ [M + H]^+^ 371.1642, found 371.1638.

### Characterization of Demethylated OTHO (**1i′**)

#### Methyl 4-(4-Hydroxyphenyl)-6-oxo-3,4-diphenylhexanoate (**1i′**)

Purified in 3:1 pentane/EtAOc; 25 mg yield (25%) as a white solid; mp 180–182 °C (from MeOH); ^1^H NMR (400 MHz, CDCl_3_) δ 7.64 (d, *J* = 7.6 Hz, 2H), 7.45 (t, *J* = 7.3 Hz, 1H), 7.34–7.27 (m, 6H), 7.21–7.12 (m, 3H), 6.72 (d, *J* = 7.7 Hz, 2H), 3.55 (td, *J* = 11.3 Hz, *J*′ = 10.2 Hz, *J*″ = 6.6 Hz, 1H), 3.47–3.21 (m, 5H), 2.93 (dd, *J* = 16.6 Hz, *J*′ = 3.2 Hz, 1H), 2.67–2.30 (m, 2H); ^13^C{^1^H} NMR (101 MHz, CDCl_3_) δ 199.3, 173.2, 155.0, 142.6, 137.1, 133.8, 133.0, 129.4 (2C), 128.8 (2C), 128.5 (2C), 128.4 (2C), 128.0 (2C), 127.0, 115.8 (2C), 51.7, 47.3 (2C), 44.0, 40.3; ATR-FTIR ν 2204, 2160, 2190, 1726, 1680, 1514, 1231, 1153 cm^–1^; HRMS (ESI) *m*/*z* calcd for C_25_H_25_O_4_ [M + H]^+^ 389.1747, found 389.1748.

#### Methyl 4-{(3*Z*)-3-[Hydroxy(phenyl)methylidene]-4-oxo-2-phenylcyclopentyl}benzoate (**2j**)

Purified in 94:6 to 93:7 petroleum ether/EtOAc; 50 mg yield as a thick yellow oil (50%); 1:8 keto:enol 1:8. Enol: ^1^H NMR (400 MHz, CDCl_3_) δ 7.79–7.73 (m, 2H), 7.53–7.50 (m, 2H), 7.39–7.35 (m, 1H), 7.28–7.24 (s, 3H), 7.13–7.05 (m, 3H), 6.87–6.81 (m, 2H), 6.78–6.75 (m, 2H), 4.39 (d, *J* = 7.4 Hz, 1H), 3.96–3.86 (m, 4H), 3.11 (dd, *J* = 17.3 Hz, *J*′ = 13.4 Hz, 1H), 2.64 (dd, *J* = 17.4 Hz, *J*′ = 7.1 Hz, 1H); ^13^C{^1^H} NMR (101 MHz, CDCl_3_) δ 208.6, 171.3, 167.0, 144.6, 140.2, 133.9, 131.4, 129.3 (2C), 128.5 (4C), 128.4 (5C), 128.2 (2C), 127.0, 113.0, 52.1, 51.9, 48.3, 39.6. Keto: ^1^H NMR (400 MHz, CDCl_3_) δ 8.02 (dt, *J* = 9.0 Hz, *J*′ = 1.5 Hz, 2H), 8.00–7.94 (m, 2H), 7.82–7.79 (m, 2H), 7.62–7.57 (m, 1H), 7.51–7.47 (m, 2H), 7.13–7.03 (m, 2H), 6.94–6.86 (m, 2H), 6.78–6.75 (m, 2H), 4.83 (d, *J* = 7.5 Hz, 1H), 4.51 (t, *J* = 7.3 Hz, 1H), 4.16 (q, *J* = 7.3 Hz, 1H), 3.87 (s, 3H), 3.05–2.92 (m, 2H); ^13^C{^1^H} NMR (101 MHz, CDCl_3_) δ 211.6, 194.2, 145.0, 138.3, 136.3, 130.0, 129.7 (2C), 129.5 (2C), 128.8 (2C), 128.6 (3C), 128.1 (4C), 127.3, 61.6, 51.0, 45.6, 44.1, 35.3, 31.0; ATR-FTIR ν 3062, 2951, 1717, 1677, 1607, 1570, 1493, 1367, 1277, 1097, 1019 cm^–1^; HRMS (ESI) *m*/*z* calcd for C_26_H_23_O_4_ [M + H]^+^ 399.1591, found 399.1607.

#### (2*Z*)-3-(4-Chlorophenyl)-2-[hydroxy(phenyl)methylidene]-4-phenylcyclopentan-1-one (**2k**)

Purified in 99:1 to 95:5 petroleum ether/EtOAc; 55 mg yield as a thick transparent oil (59%); 1:4 keto:enol. Enol: ^1^H NMR (400 MHz, CDCl_3_) δ 7.52–7.44 (m, 2H), 7.42–7.35 (m, 1H), 7.30–7.26 (m, 1H), 7.22–7.16 (m, 1H), 7.16–7.09 (m, 3H), 7.09–7.02 (m, 2H), 6.85–6.74 (m, 2H), 6.74–6.64 (m, 2H), 4.35 (d, *J* = 7.4 Hz, 1H), 3.93–3.82 (m, 1H), 3.09–2.94 (m, 1H), 2.67–2.57 (m, 1H); ^13^C{^1^H} NMR (101 MHz, CDCl_3_) δ 212.1, 171.7, 139.5, 138.9, 131.8, 130.2 (2C), 130.0, 128.8 (2C), 128.7 (2C), 128.6 (2C), 128.5 (4C), 128.4, 127.4, 113.2, 51.7, 48.5, 39.9. Keto: ^1^H NMR (400 MHz, CDCl_3_) δ 8.01–7.96 (m, 2H), 7.62–7.56 (m, 1H), 7.52–7.44 (m, 1H), 7.30–7.26 (m, 1H), 7.22–7.16 (m, 2H), 7.16–7.09 (m, 3H), 6.85–6.74 (m, 2H), 6.74–6.64 (m, 2H), 4.76 (d, *J* = 9.1 Hz, 1H), 4.56–4.47 (m, 1H), 4.06–3.99 (q, *J* = 6.7 Hz, 1H), 3.09–2.94 (m, 1H); ^13^C{^1^H} NMR (101 MHz, CDCl_3_) δ 212.1, 194.5, 139.7, 137.5, 134.2, 134.2, 132.9, 130.0, 129.8, 129.1, 128.8, 128.8 (2C), 128.7(2C), 128.6 (2C), 128.4 (2C), 127.5, 61.3, 50.7, 45.6, 45.4; ATR-FTIR ν 2956, 2915, 1546, 1566, 1489, 1351, 1257, 1227, 1088, 1014 cm^–1^; HRMS (ESI) *m*/*z* calcd for C_24_H_20_ClO_2_ [M + H]^+^ 375.1146, found 375.1145.

#### (2*Z*)-3-(4-Bromophenyl)-2-[hydroxy(phenyl)methylidene]-4-phenylcyclopentan-1-one (**2l**)

Purified in 99:1 to 97:3 petroleum ether/EtOAc; 75 mg yield as a thick yellow oil (72%); 1:10 keto:enol. Enol: ^1^H NMR (400 MHz, CDCl_3_) δ 7.54–7.46 (m, 2H), 7.44–7.36 (m, 1H), 7.29–7.25 (m, 2H), 7.24–7.19 (m, 2H), 7.16–7.10 (m, 3H), 6.82–6.74 (m, 2H), 6.69–6.58 (m, 2H), 4.34 (d, *J* = 7.4 Hz, 1H), 3.94–3.81 (m, 1H), 3.04 (dd, *J* = 17.4 Hz, *J*′ = 13.5 Hz, 1H), 2.63 (dd, *J* = 17.4 Hz, *J*′ = 7.1 Hz, 1H); ^13^C{^1^H} NMR (101 MHz, CDCl_3_) δ 208.9, 171.3, 139.6, 138.5, 133.8, 131.4, 131.3 (2C), 130.2 (2C), 128.4 (2C), 128.3 (2C), 128.2 (2C), 128.1 (2C), 127.1, 120.7, 112.7, 51.4, 48.1, 39.5. Keto: ^1^H NMR (400 MHz, CDCl_3_) δ 8.04–7.97 (m, 2H), 7.64–7.56 (m, 1H), 7.53–7.45 (m, 1H), 7.30–7.24 (m, 1H), 7.24–7.18 (m, 2H), 7.16–7.11 (m, 3H), 6.84–6.79 (s, 2H), 6.68–6.62 (m, 2H), 4.77 (d, *J* = 9.2 Hz, 1H), 4.51 (dd, *J* = 9.2 Hz, *J*′ = 6.9 Hz, 1H), 4.03 (q, *J* = 6.7 Hz, 1H), 2.97 (d, *J* = 6.6 Hz, 1H); ^13^C{^1^H} NMR (101 MHz, CDCl_3_) δ 211.6, 194.1, 139.3, 137.7, 136.5, 133.9, 131.3, 129.8 (2C), 129.6 (2C), 128.8 (2C), 128.4 (2C), 128.1 (2C), 127.1 (2C), 121.0, 60.8, 50.4, 45.2, 45.0; ATR-FTIR ν 2959, 1742, 1644, 1592, 1565, 1486, 1351, 1230 cm^–1^; HRMS (ESI) *m*/*z* calcd for C_24_H_20_BrO_2_ [M + H]^+^ 419.0641, found 419.0641.

#### (2*Z*)-2-[Hydroxy(phenyl)methylidene]-3-(4-methylphenyl)-4-phenylcyclopentan-1-one (**2m**)

Purified in 98:2 to 97:3 petroleum ether/EtOAc; 62 mg yield as a thick yellow oil (70%); 1:10 keto:enol. Enol: ^1^H NMR (400 MHz, CDCl_3_) δ 7.61–7.54 (m, 2H), 7.43–7.34 (m, 1H), 7.30–7.26 (s, 2H), 7.16–7.07 (m, 3H), 6.95–6.89 (m, 2H), 6.84–6.76 (m, 2H), 6.71–6.64 (m, 2H), 4.35 (d, *J* = 7.3 Hz, 1H), 3.85 (dt, *J* = 14.0 Hz, *J*′ = 7.1 Hz, 1H), 3.10 (dd, *J* = 17.3 Hz, *J*′ = 13.7 Hz, 1H), 2.62 (dd, *J* = 17.2 Hz, *J*′ = 7.0 Hz, 1H), 2.26 (s, 3H); ^13^C{^1^H} NMR (101 MHz, CDCl_3_) δ 209.5, 170.5, 139.0, 137.3, 136.2, 134.0, 131.2, 128.9 (2C), 128.5 (2C), 128.4 (2C), 128.3 (2C), 128.2 (2C), 127.9 (2C), 126.7, 113.6, 51.6, 48.4, 39.8, 21.1. Keto: ^1^H NMR (400 MHz, CDCl_3_) δ 8.07–7.99 (m, 2H), 7.52–7.45 (m, 2H), 7.30–7.26 (s, 2H), 7.20–7.17 (m, 3H), 6.95–6.89 (m, 1H), 6.88–6.82 (m, 2H), 6.72–6.62 (m, 2H), 4.83 (d, *J* = 8.3 Hz, 1H), 4.48 (dd, *J* = 8.3 Hz, *J*′ = 6.9 Hz, 1H), 4.07 (q, *J* = 7.0 Hz, 1H), 3.02–2.91 (m, 2H), 2.24 (s, 3H); ^13^C{^1^H} NMR (101 MHz, CDCl_3_) δ 212.5, 194.7, 139.7, 136.6, 136.5, 135.5, 133.7, 129.6 (2C), 128.9 (2C), 128.7 (2C), 128.2 (2C), 128.1 (2C), 128.0 (2C), 126.8, 61.4, 50.8, 45.5, 44.8, 21.1; ATR-FTIR ν 2917, 1740, 1606, 1569, 1494, 1369, 1265 cm^–1^; HRMS (ESI) *m*/*z* calcd for C_25_H_23_O_2_ [M + H]^+^ 355.1693, found 355.1701.

#### 4-{(2*Z*)-2-[Hydroxy(phenyl)methylidene]-3-methylidene-5-phenylcyclopentyl}benzonitrile (**2n**)

Purified in 9:1 to 4:1 pentane/EtOAc; 72 mg yield as a thick pink oil (79%); >1:7 keto:enol. Enol: ^1^H NMR (400 MHz, CDCl_3_) δ 7.48–7.41 (m, 2H), 7.36 (dd, *J* = 9.5 Hz, *J*′ = 7.8 Hz, 3H), 7.28–7.24 (m, 2H), 7.12 (dt, *J* = 5.4 Hz, *J*′ = 2.7 Hz, 3H), 6.87 (d, *J* = 8.1 Hz, 2H), 6.76 (dd, *J* = 7.1 Hz, *J*′ = 2.4 Hz, 2H), 4.48 (d, *J* = 7.6 Hz, 1H), 3.95 (dt, *J* = 12.9 Hz, *J*′ = 7.4 Hz, 1H), 3.03 (dd, *J* = 17.5 Hz, *J*′ = 13.0 Hz, 1H), 2.69 (dd, *J* = 17.5 Hz, *J*′ = 7.2 Hz, 1H); ^13^C{^1^H} NMR (101 MHz, CDCl_3_) δ 208.2, 172.2, 146.4, 138.1, 133.6, 131.9 (2C), 131.6, 129.1 (2C), 128.6, 128.4 (2C), 128.3 (2C), 128.1 (2C), 127.9 (2C), 127.3, 111.9, 110.6, 52.0, 47.9, 39.4. Keto: ^1^H NMR (400 MHz, CDCl_3_) δ 8.06–7.97 (m, 2H), 7.61 (t, *J* = 7.4 Hz, 1H), 7.50 (t, *J* = 7.7 Hz, 2H), 7.21–7.17 (m, 3H), 6.89–6.86 (m, 3H), 6.81–6.78 (m, 3H), 4.82 (d, *J* = 9.6 Hz, 1H), 4.64 (dd, *J* = 9.6 Hz, *J*′ = 6.9 Hz, 1H), 4.06 (td, *J* = 7.1 Hz, *J*′ = 5.1 Hz, 1H), 3.06–2.96 (m, 2H); ^13^C{^1^H} NMR (101 MHz, CDCl_3_) δ 210.7, 193.5, 144.3, 139.0, 136.3, 134.0, 132.0 (2C), 129.7 (2C), 128.9, 128.8 (2C), 128.6 (2C), 128.4 (2C), 128.3 (2C), 127.4, 118.8, 110.9, 60.2, 50.7, 45.1; ATR-FTIR ν 3029, 2228, 1743, 1607, 1570, 1493, 1361, 1227, 1151 cm^–1^; HRMS (ESI) *m*/*z* calcd for C_25_H_20_NO_2_ [M + H]^+^ 366.1489, found 366.1489.

#### (2*Z*)-2-[Hydroxy(4-methylphenyl)methylidene]-3,4-diphenylcyclopentan-1-one (**2o**)

Purified in 99:1 to 95:5 petroleum ether/EtOAc; 52 mg yield as a thick transparent oil (56%); >1:20 keto:enol. Enol: ^1^H NMR (400 MHz, CDCl_3_) δ 7.51–7.45 (m, 2H), 7.27–7.21 (m, 2H), 7.17–7.04 (m, 6H), 6.80–6.73 (m, 4H), 4.33 (d, *J* = 7.4 Hz, 1H), 3.94–3.83 (m, 1H), 3.59–3.03 (m, 1H), 2.68–2.59 (m, 1H); ^13^C{^1^H} NMR (101 MHz, CDCl_3_) δ 212.6, 212.3, 209.7, 193.5, 169.9, 140.7, 140.6, 139.8, 139.1, 138.8, 137.8, 135.2, 132.7, 131.4, 130.1, 129.4, 129.0, 128.9, 128.7, 128.5, 128.3, 127.4, 127.2, 113.4, 77.2, 61.6, 52.3, 48.6, 45.8, 45.2, 40.1; ATR-FTIR ν 3027, 1591, 1487, 1360, 1259, 1090, 1011 cm^–1^; HRMS (ESI) *m*/*z* calcd for C_24_H_20_ClO_2_ [M + H]^+^ 375.1139, found 375.1146.

#### (2*Z*)-2-[(4-Bromophenyl)(hydroxy)methylidene]-3,4-diphenylcyclopentan-1-one (**2p**)

Purified in 98:2 to 96:4 petroleum ether/EtOAc; 79 mg yield as a thick yellow oil (75%); 1:18 keto:enol. Enol: ^1^H NMR (400 MHz, CDCl_3_) δ 7.42–7.38 (m, 4H), 7.14–7.06 (m, 6H), 6.81–6.72 (m, 4H), 4.33 (d, *J* = 7.4 Hz, 1H), 3.88 (dt, *J* = 14.1 Hz, *J*′ = 7.3 Hz, 1H), 3.10 (dd, *J* = 17.4 Hz, *J*′ = 13.6 Hz, 1H), 2.64 (dd, *J* = 17.4 Hz, *J*′ = 7.1 Hz, 1H); ^13^C{^1^H} NMR (101 MHz, CDCl_3_) δ 209.4, 169.6, 140.2, 138.7, 132.8, 131.6 (2C), 129.9 (2C), 128.5 (2C), 128.4 (2C), 128.2 (2C), 128.0 (2C), 126.9, 126.9, 126.0, 113.5, 51.9, 48.2, 39.8. Keto: ^1^H NMR (400 MHz, CDCl_3_) δ 7.89 (d, *J* = 8.7 Hz, 2H), 7.62 (d, *J* = 8.5 Hz, 2H), 7.56–7.52 (m, 1H), 7.35–7.29 (m, 1H), 7.18–7.07 (m, 6H), 6.84–6.73 (m, 2H), 4.77 (d, *J* = 8.4 Hz, 1H), 4.52 (dd, *J* = 8.6 Hz, *J*′ = 6.9 Hz, 1H), 4.06 (q, *J* = 7.0 Hz, 1H), 3.01–2.94 (m, 2H); ^13^C{^1^H} NMR (101 MHz, CDCl_3_) δ 211.9, 193.3, 139.4, 138.5, 135.3, 132.1 (2C), 131.2 (2C), 128.3 (2C), 128.2 (2C), 128.1 (4C), 127.1, 127.0, 126.0, 61.2, 50.9, 45.4, 44.8; ATR-FTIR ν 1588, 1485, 1359, 1257, 1234, 1071, 1007 cm^–1^; HRMS (ESI) *m*/*z* calcd for C_24_H_20_BrO_2_ [M + H]^+^ 419.0641, found 419.0638.

#### (2*Z*)-2-[Hydroxy(4-methylphenyl)methylidene]-3,4-diphenylcyclopentan-1-one (**2q**)

Purified in 99:1 to 95:5 petroleum ether/EtOAc; 51 mg yield as a thick transparent oil (58%); 1:10 keto:enol. Enol: ^1^H NMR (400 MHz, CDCl_3_) δ 7.49–7.44 (m, 2H), 7.14–7.05 (m, 8H), 6.85–6.69 (m, 4H), 4.38 (d, *J* = 7.4 Hz, 1H), 3.87 (dt, *J* = 13.6 Hz, *J*′ = 7.2 Hz, 1H), 3.09 (dd, *J* = 17.3 Hz, *J*′ = 13.6 Hz, 1H), 2.62 (dd, *J* = 17.3 Hz, *J*′ = 7.1 Hz, 1H), 2.32 (s, 3H); ^13^C{^1^H} NMR (101 MHz, CDCl_3_) δ 209.0, 171.2, 141.9, 140.6, 139.0, 131.1, 129.1 (2C), 128.6 (2C), 128.5 (2C), 128.2 (4C), 128.0 (2C), 126.8, 126.7, 112.8, 52.1, 48.4, 39.7, 21.6. Keto: ^1^H NMR (400 MHz, CDCl_3_) δ 7.93 (d, *J* = 8.3 Hz, 2H), 7.28 (dt, *J* = 8.0 Hz, *J*′ = 0.8 Hz, 3H), 7.18–7.14 (m, 3H), 7.11–7.04 (m, 2H), 6.90–6.88 (m, 2H), 6.83–6.81 (m, 4H), 4.82 (d, *J* = 8.2 Hz, 1H), 4.51 (dd, *J* = 8.2 Hz, *J*′ = 7.0 Hz, 1H), 4.08 (q, *J* = 7.0 Hz, 1H), 3.01–2.91 (m, 1H), 2.42 (s, 3H); ^13^C{^1^H} NMR (101 MHz, CDCl_3_) δ 212.5, 194.0, 144.8, 139.7, 138.8, 134.1, 129.8 (2C), 129.5 (2C), 128.2 (6C), 128.1 (2C), 127.0, 126.9, 61.2, 51.1, 45.5, 44.7 21.8; ATR-FTIR ν 3022, 1744, 1632, 1595, 1495, 1364, 1225, 1149, 1098 cm^–1^; HRMS (ESI) *m*/*z* calcd for C_25_H_23_O_2_ [M + H]^+^ 355.1693, found 355.1698.

#### Methyl 4-{Hydroxy[(1*Z*)-5-oxo-2,3-diphenylcyclopentylidene]methyl}benzoate (**2r**)

Purified in 99:1 to 95:5 petroleum ether/EtOAc; 41 mg yield as a thick transparent oil (41%); >1:20 keto:enol. Enol: ^1^H NMR (400 MHz, CDCl_3_) δ 8.06–7.77 (m, 2H), 7.68–7.48 (m, 2H), 7.16–7.00 (m, 6H), 6.82–6.69 (m, 4H), 4.34 (d, *J* = 7.4 Hz, 1H), 3.88 (s, 3H), 3.10 (dd, *J* = 17.5 Hz, *J*′ = 13.5 Hz, 1H), 2.65 (dd, *J* = 17.4 Hz, *J*′ = 7.1 Hz, 1H); ^13^C{^1^H} NMR (101 MHz, CDCl_3_) δ 209.9, 169.2, 166.4, 140.3, 138.7, 138.0, 132.1, 129.5 (2C), 128.6 (2C), 128.4 (2C), 128.3 (2C), 128.2 (2C), 128.0 (2C), 126.9, 114.3, 77.2, 52.5, 51.9, 48.2, 39.9; ATR-FTIR ν 2957, 1712, 1636, 1593, 1426, 1371, 1283, 1112, 1018 cm^–1^; HRMS (ESI) *m*/*z* calcd for C_26_H_23_O_4_ [M + H]^+^ 399.1591, found 399.1597.

#### 4-{Hydroxy[(1*Z*)-5-oxo-2,3-diphenylcyclopentylidene]methyl}benzonitrile (**2s**)

Purified in 9:1 to 4:1 pentane/EtOAc; 55 mg yield as an orange solid (60%); mp 122–125 °C; >1:10 keto:enol. Enol: ^1^H NMR (400 MHz, CDCl_3_) δ 7.61 (d, *J* = 8.5 Hz, 2H), 7.53 (d, *J* = 8.5 Hz, 2H), 7.15–7.05 (m, 6H), 6.75 (td, *J* = 7.5 Hz, *J*′ = 2.0 Hz, 4H), 4.32 (d, *J* = 7.4 Hz, 1H), 3.89 (dt, *J* = 13.0 Hz, *J*′ = 7.2 Hz, 1H), 3.10 (dd, *J* = 17.5 Hz, *J*′ = 13.4 Hz, 1H), 2.67 (dd, *J* = 17.5 Hz, *J*′ = 7.1 Hz, 1H); ^13^C{^1^H} NMR (101 MHz, CDCl_3_) δ 210.1, 167.7, 139.9, 138.4, 138.1, 132.0 (2C), 128.8 (2C), 128.5 (2C), 128.4 (2C), 128.1 (4C), 127.1, 127.0, 114.7, 114.4 (2C), 51.7, 48.1, 39.9. Keto: ^1^H NMR (400 MHz, CDCl_3_) δ 8.10 (d, *J* = 8.2 Hz, 2H), 7.78 (d, *J* = 8.2 Hz, 2H), 7.18–7.13 (m, 6H), 6.84–6.74 (m, 4H), 4.79 (d, *J* = 8.9 Hz, 1H), 4.53 (dd, *J* = 8.9 Hz, *J*′ = 6.9 Hz, 1H), 4.05 (q, *J* = 6.8 Hz, 1H), 2.99 (dd, *J* = 6.7 Hz, *J*′ = 4.6 Hz, 2H); ^13^C{^1^H} NMR (101 MHz, CDCl_3_) δ 211.4, 193.1, 139.5, 139.2, 138.2, 132.5 (2C), 130.0 (2C), 128.3 (4C), 128.0 (4C), 127.2, 127.1, 116.9, 114.4, 61.5, 50.8, 45.4, 45.0; ATR-FTIR ν 3030, 1637, 1585, 1495, 1345, 1261, 1224, 1148, cm^–1^; HRMS (ESI) *m*/*z* calcd for C_25_H_20_NO_2_ [M + H]^+^ 366.1489, found 366.1494.

#### (*Z*)-2-[Hydroxy(phenyl)methylene]-3-(4-methoxyphenyl)-4-phenylcyclopentan-1-one (**2t**)

Purified in 8:1 to 4:1 pentane/EtOAc; 43 mg yield as a yellow semisolid (46%); >1:10 keto:enol. Enol: ^1^H NMR (400 MHz, CDCl_3_) δ 7.55–7.53 (m, 2H), 7.41–7.33 (m, 1H), 7.31–7.22 (m, 2H), 7.14–7.05 (m, 3H), 6.79–6.76 (m, 2H), 6.72–6.59 (m, 4H), 4.31 (d, *J* = 7.2 Hz, 1H), 3.89–3.78 (m, 1H), 3.73 (s, 3H), 3.06 (dd, *J* = 17.3 Hz, *J*′ = 13.6 Hz, 1H), 2.60 (dd, *J* = 17.3 Hz, *J*′ = 7.0 Hz, 1H); ^13^C{^1^H} (101 MHz, CDCl_3_) δ 209.5, 170.7, 158.4, 139.1, 132.6, 131.3, 129.6 (3C), 128.5 (2C), 128.4 (2C), 128.3 (2C), 128.0 (2C), 126.8, 113.7 (2C), 113.6, 55.3, 51.2, 48.4, 39.8. Keto: ^1^H NMR (400 MHz, CDCl_3_) δ 8.00–7.98 (m, 1H), 7.49–7.46 (m, 3H), 7.26–7.24 (m, 2H), 7.18–7.15 (m, 3H), 6.84–6.80 (m, 2H), 6.72–6.62 (m, 4H), 4.76 (d, *J* = 8.4 Hz, 1H), 4.44 (dd, *J* = 8.4 Hz, *J*′ = 6.9 Hz, 1H), 4.02 (q, *J* = 6.8 Hz, 1H), 3.71 (s, 3H), 2.98–2.91 (m, 2H); ^13^C{^1^H} NMR (101 MHz, CDCl_3_) δ 212.6, 194.8, 139.8, 136.7, 133.8, 130.7, 129.6 (2C), 129.2 (2C), 128.8 (2C), 128.4, 128.3 (2C), 128.2 (2C), 128.0, 126.9, 113.7 (2C), 61.6, 50.5, 45.5, 44.9; ATR-FTIR ν 2934, 1741, 1608, 1570, 1509, 1246 cm^–1^; HRMS (ESI) *m*/*z* calcd for C_25_H_22_O_3_Na [M + Na]^+^ 393.1461, found 393.1467.

#### Bromination of **2a** with Pyridinium Tribromide

Diketo compound **2a** (210 mg, 0.62 mmol, 1 equiv) was dissolved in DCM (18 mL) and cooled to 0 °C. Then PyrHBr·Br_2_ (220 mg, 90%, 0.62 mmol, 1 equiv) was added at once while the reaction mixture was vigorously stirred. The reaction mixture was then stirred overnight at room temperature. A saturated solution of NaHCO_3_ was added; the organic phase was extracted and washed with water and dried, and the solvent was removed in vacuo. The crude mixture was purified by automated column chromatography with a 98:2 petroleum ether/EtOAc eluent, yielding product **4** as a pale orange solid.

#### (5*Z*)-2-Bromo-5-[hydroxy(phenyl)methylidene]-3,4-diphenylcyclopentan-1-one (**4**)

Yield 250 mg (97%); mp 120–122 °C (from MeOH); >1:20 keto:enol; ^1^H NMR (400 MHz, CDCl_3_) δ 7.58–7.52 (m, 2H), 7.45–7.38 (m, 1H), 7.30–7.26 (m, 2H), 7.20–7.09 (m, 6H), 6.77–6.74 (m, 4H), 5.22 (dd, *J* = 12.3 Hz, *J*′ = 1.2 Hz, 1H), 4.43 (d, *J* = 7.6 Hz, 1H), 3.99 (dd, *J* = 12.3 Hz, *J*′ = 7.6 Hz, 1H); ^13^C{^1^H} NMR (101 MHz, CDCl_3_) δ 200.8, 173.2, 139.5, 136.0, 133.1, 131.9, 128.6 (2C), 128.5 (4C), 128.3 (2C), 128.2 (4C), 127.4, 127.2, 110.3, 58.4, 52.3, 51.2; ATR-FTIR ν 3058, 1637, 1591, 1567, 1491, 1447, 1356, 1142, 1076 cm^–1^; HRMS (ESI) *m*/*z* calcd for C_24_H_20_BrO_2_ [M + H]^+^ 419.0641, found 419.0635.

#### Bromination of **2a** with *N*-Bromosuccinimide

According to the published procedure,^52^ a solution of NBS (26 mg, 0.15 mmol, 1 equiv) in DCM (2 mL) was added to a solution of 1,3-diketone **2a** (50 mg, 0.15 mmol, 1 equiv) at rt. The reaction mixture was allowed to stir at room temperature for 6 h until full conversion was achieved. Then the reaction mixture was directly purified by automated column chromatography with a petroleum ether/EtOAc gradient (98:2 to 97:3), yielding the desired compound **5** as a single diastereoisomer as an off-white oil that was crystallized from methanol to give a pale off-white solid.

#### 2-Benzoyl-2-bromo-3,4-diphenylcyclopentan-1-one (**5**)

Yield 52 mg (84%); mp 112–115 °C (from MeOH); ^1^H NMR (400 MHz, CDCl_3_) δ 7.93–7.82 (m, 2H), 7.45–7.36 (m, 1H), 7.30–7.26 (s, 2H), 7.11–7.08 (m, 3H), 6.98–6.81 (m, 5H), 6.64–6.56 (m, 2H), 4.64–4.52 (m, 1H), 4.41 (dd, *J* = 6.5 Hz, *J*′ = 1.3 Hz, 1H), 3.11 (dd, *J* = 19.1 Hz, *J*′ = 13.3 Hz, 1H), 2.92 (ddd, *J* = 19.1 Hz, *J*′ = 7.9 Hz, *J*″ = 1.4 Hz, 1H); ^13^C{^1^H} NMR (101 MHz, CDCl_3_) δ 204.4, 190.9, 137.2, 135.5, 135.1, 132.9, 129.4, 129.1, 128.2, 128.0, 127.9, 127.4, 127.1, 77.2, 63.3, 61.6, 42.6, 39.1; ATR-FTIR ν 3029, 1757, 1664, 1596, 1447, 1252, 1234, 1142, 1072 cm^–1^; HRMS (ESI) *m*/*z* calcd for C_24_H_20_BrO_2_ [M + H]^+^ 419.0641, found 419.0631.

#### Synthesis of **6**

To diketo compound **2a** (60 mg, 0.176 mmol, 1 equiv) in DCM (3 mL) was added at rt a solution of *N*-(phenylseleno)phthalimide (90 mg, 90%, 0.26 mmol, 1.5 equiv). The reaction mixture stirred for 3 h until full conversion was achieved, and the solvent was removed in vacuo. The crude mixture was then dissolved in EtOAc (6 mL), and hydrogen peroxide (2 mL of 30% in water) was added. After 3 h, full conversion was achieved, the reaction mixture was diluted with water (1 mL), and the organic phase was collected and washed with saturated NaHCO_3_ and water and dried over MgSO_4_. After filtration and evaporation of the solvent, the crude product was separated by automated column chromatography with a petroleum ether/EtOAc gradient (93:7 to 85:15), yielding product **6** as a transparent oil.

#### 2-Benzoyl-3,4-diphenylcyclopent-2-en-1-one (**6**)

Yield 42 mg (70%); ^1^H NMR (400 MHz, CDCl_3_) δ 7.94–7.84 (m, 1H), 7.59–7.49 (m, 1H), 7.44–7.40 (m, 2H), 7.31–7.13 (m, 10H), 4.80 (dd, *J* = 7.4 Hz, *J*′ = 2.3 Hz, 1H), 3.30 (dd, *J* = 19.0 Hz, *J*′ = 7.4 Hz, 1H), 2.67 (dd, *J* = 19.0 Hz, *J*′ = 2.3 Hz, 1H); ^13^C{^1^H} NMR (101 MHz, CDCl_3_) δ 204.2, 194.6, 172.8, 141.5, 136.1, 134.4, 134.2, 130.8, 129.5 (2C), 129.3 (2C), 128.9 (2C), 128.7 (4C), 127.5 (2C), 127.3, 123.7, 47.3, 46.3; ATR-FTIR ν 3057, 1690, 1654, 1597, 1579, 1497, 1331, 1236, 1174, 1052 cm^–1^; HRMS (ESI) *m*/*z* calcd for C_24_H_19_O_2_ [M + H]^+^ 339.1385, found 339.1389.

#### Hydroxylation of **2a**

To the mixture of **2a** (120 mg, 0.35 mmol, 1 equiv) and 0.5 M aqueous NaHCO_3_ (0.7 mL, 1 equiv) in DCM (4 mL) was added *m*-CPBA (198 mg, 77%, 0.88 mmol, 2.5 equiv) portionwise at ambient temperature. After 3 h, full conversion was achieved according to TLC analysis. The excess of peracid was quenched by adding 15% aqueous Na_2_SO_3_ (3 mL), and the resulting mixture was stirred for an additional 1 h at room temperature. The organic layer was then separated, washed with brine (5 mL), dried over MgSO_4_, and filtered and concentrated in vacuo. The residue was purified by automated column chromatography with a petroleum ether/EtOAc gradient (98:2 to 85:15), yielding product **7** as a single diastereoisomer as a thick transparent oil.

#### 2-Benzoyl-2-hydroxy-3,4-diphenylcyclopentan-1-one (**7**)

Yield 56 mg (45%); ^1^H NMR (400 MHz, CDCl_3_) δ 7.94–7.63 (m, 2H), 7.42–7.34 (m, 1H), 7.27–7.19 (m, 2H), 7.15–7.04 (m, 3H), 7.00–6.97 (m, 2H), 6.87–6.84 (m, 3H), 6.76–6.73 (m, 2H), 4.32 (dt, *J* = 14.3 Hz, *J*′ = 7.6 Hz, 1H), 4.19 (dd, *J* = 8.0 Hz, *J*′ = 1.2 Hz, 1H), 3.50 (dd, *J* = 17.8 Hz, *J*′ = 13.7 Hz, 1H), 2.95 (ddd, *J* = 17.8 Hz, *J*′ = 7.3 Hz, *J*″ = 1.4 Hz, 1H); ^13^C{^1^H} NMR (101 MHz, CDCl_3_) δ 213.5, 198.4, 138.1, 135.1, 135.1, 133.1, 130.4 (2C), 129.9 (2C), 128.2 (2C), 128.0 (4C), 127.9 (2C), 127.0, 126.7, 87.8, 60.3, 42.8, 41.6; ATR-FTIR ν 3396, 3029, 1748, 1665, 1596, 1448, 1251, 1039 cm^–1^; HRMS (ESI) *m*/*z* calcd for C_24_H_21_O_3_ [M + H]^+^ 357.1485, found 357.1499.

#### Reaction of **2a** with Hydrazine Hydrate

According to the published procedure,^56^ to a sealed vial with **2a** (168 mg, 0.49 mmol, 1 equiv) in EtOH (4 mL) was added hydrazine hydrate (37 μL, 65% in water, 1 equiv). The reaction mixture was then heated on an oil bath to reflux until full conversion was achieved as indicated by TLC. The solvent was then removed in vacuo, and the reaction mixture was then purified by automated column chromatography with a petroleum ether/EtOAc gradient (80:20 to 67:33), yielding pyrazole derivative **8** as a yellow thick oil (110 mg, 66%).

#### 3,4,5-Triphenyl-2*H*,4*H*,5*H*,6*H*-cyclopenta[*c*]pyrazole (**8**)

Yield 110 mg (66%); ^1^H NMR (400 MHz, CDCl_3_) δ 10.19 (bs, 1H), 7.54–7.37 (m, 2H), 7.28–7.19 (m, 3H), 7.13–7.06 (m, 3H), 7.03–6.99 (m, 3H), 6.88–6.86 (m, 2H), 6.74–6.65 (m, 2H), 4.57 (d, *J* = 7.8 Hz, 1H), 4.47 (dtd, *J* = 10.2 Hz, *J*′ = 7.7 Hz, *J*″ = 2.1 Hz, 1H), 3.24 (dd, *J* = 15.3 Hz, *J*′ = 10.9 Hz, 1H), 3.07 (ddd, *J* = 15.4 Hz, *J*′ = 7.7, *J*″ = 2.0 Hz, 1H); ^13^C{^1^H} NMR (101 MHz, CDCl_3_) δ 140.2, 140.2, 130.3, 129.0 (4C), 128.7 (2C), 128.6 (2C), 128.0 (2C), 127.9 (2C), 127.8 (2C), 126.5, 126.4, 125.8, 125.8, 58.1, 49.4, 29.1; ATR-FTIR ν 3026, 2898, 1602, 1493, 1452, 1314, 1233, 1180, 1109 cm^–1^; HRMS (ESI) *m*/*z* calcd for C_24_H_21_N_2_ [M + H]^+^ 337.1699, found 337.1704.

#### Methylation of **2a**

To the starting **2a** (0.85 mmol, 290 mg, 1equiv) in DMF (5 mL) under argon was added K_2_CO_3_ (0.94 mmol, 180 mg, 1.5 equiv), and then methyl iodide (0.94 mmol, 60 μL, 1.1 equiv) was slowly added. The reaction mixture was then stirred overnight at 60 °C (oil bath). The next day, the reaction was quenched with water (5 mL) and the mixture washed with EtOAc (3 × 50 mL). The combined organic layer was washed with brine (2 × 30 mL) and dried over MgSO_4_. After filtration, the solvent was evaporated in vacuo and the crude material was separated by automated column chromatography with a pentane/EtOAc gradient (85:15 to 4:1), yielding product **9** as a thick transparent oil.

#### 2-Benzoyl-2-methyl-3,4-diphenylcyclopentan-1-one and 2-[Methoxy(phenyl)methylidene]-3,4-diphenylcyclopentan-1-one (**9a**:**9b**)

Yield 210 mg (70%); 4:1 keto:enol ether. Keto: ^1^H NMR (400 MHz, CDCl_3_) δ 7.68–7.56 (m, 2H), 7.35–7.31 (m, 1H), 7.29–7.19 (m, 2H), 7.13–7.05 (m, 4H), 6.94–6.87 (m, 4H), 6.77–6.75 (m, 2H), 4.15–4.11 (m, 1H), 4.08 (t, *J* = 6.9 Hz, 1H), 3.28 (dd, *J* = 18.0 Hz, *J*′ = 13.5 Hz, 1H), 2.73 (dd, *J* = 18.5 Hz, *J*′ = 6.2 Hz, 1H), 1.85 (s, 3H); ^13^C{^1^H} NMR (101 MHz, CDCl_3_) δ 214.1, 199.2, 137.9, 137.2, 136.2, 132.0, 129.5, 128.7 (2C), 128.0 (6C), 127.9 (2C), 127.6 (2C), 126.7, 65.8, 60.2, 43.2, 40.6, 24.7. Enol: ^1^H NMR (400 MHz, CDCl_3_) δ 7.77–7.69 (m, 2H), 7.47–7.44 (m, 1H), 7.40–7.37 (m, 2H), 7.03–6.97 (m, 3H), 6.87 (s, 4H), 4.58 (d, *J* = 8.2 Hz, 1H), 4.01 (q, *J* = 8.8 Hz, 1H), 3.77 (s, 3H), 3.22–3.15 (m, 1H), 3.01 (dd, *J* = 16.8 Hz, *J*′ = 8.1 Hz, 1H); ^13^C{^1^H} NMR (101 MHz, CDCl_3_) δ 192.4, 167.6, 140.3, 140.1, 139.7, 131.3, 128.6 (2C), 128.2 (2C), 127.7 (4C), 126.7 (4C), 126.3, 126.1, 116.2, 58.0, 54.4, 46.7, 35.3; ATR-FTIR ν 3026, 1745, 1669, 1596, 1454, 1246 cm^–1^; HRMS (ESI) *m*/*z* calcd for C_25_H_23_O_2_ [M + H]^+^ 355.1698, found 355.1700.

#### Aldol Reaction of **2a** with Benzophenone

According to the modified procedure,^55^ diisopropylamine (91 μL, 2.20 equiv) was dissolved in dry tetrahydrofuran (5 mL), and after the mixture had cooled to 0 °C, *n*-butyllithium (260 μL, 2.20 equiv, 2.5 M solution in *n*-hexane) was added. The reaction mixture was stirred at rt for 30 min followed by further cooling to 0 °C. Diketone **2a** (100 mg, 0.29 mmol, 1 equiv) was dissolved in dry tetrahydrofuran (3 mL) and slowly added to the solution of diisopropylamine and *n*-butyllithium, and the mixture was stirred. After 15 min, benzophenone (65 mg, 0.35 mmol, 1.20 equiv) was added and the reaction mixture stirred at 0 °C for 30 min and then at rt overnight. After 16 h, TLC revealed full conversion; the reaction was then quenched by the addition of a saturated aqueous solution of ammonium chloride (1 mL) and water (10 mL), and EtAOc was then added. The organic phase was separated, and the aqueous phase extracted with EtOAc (2 × 20 mL). The combined organic phases were washed with brine and dried over MgSO_4_, and the solvent was evaporated in vacuo. The crude material was purified by automated column chromatography with a petroleum ether/EtOAc gradient (95:5 to 92:8), yielding product **10** as a transparent oil.

#### (2*Z*)-2-[Hydroxy(phenyl)methylidene]-5-(hydroxydiphenylmethyl)-3,4-diphenylcyclopentan-1-one (**10**)

Yield 100 mg (65%); >1:20 keto/enol; ^1^H NMR (400 MHz, CDCl_3_) δ 7.50 (ddd, *J* = 15.3 Hz, *J*′ = 8.2 Hz, *J*″ = 1.2 Hz, 4H), 7.39–7.31 (m, 1H), 7.28–7.18 (m, 6H), 7.05–6.93 (m, 5H), 6.86–6.60 (m, 5H), 6.33 (bs, 2H), 4.36 (d, *J* = 10.6 Hz, 1H), 4.25 (d, *J* = 8.2 Hz, 1H), 4.04 (dd, *J* = 10.6 Hz, *J*′ = 8.1 Hz, 1H), 3.87 (s, 1H); ^13^C{^1^H} NMR (101 MHz, CDCl_3_) δ 208.7, 172.9, 146.1, 143.9, 141.4, 139.5, 133.8, 131.4, 128.6 (4C), 128.2 (4C), 128.1 (2C), 127.9 (2C), 127.5 (2C), 127.3 (2C), 127.2, 127.0 (4C), 126.8, 126.5, 125.4, 113.5, 79.7, 57.7, 51.0, 50.8; ATR-FTIR ν 3061, 3028, 2956, 1738, 1603, 1568, 1492, 1447, 1365, 1269, 1218, 1154 cm^–1^; HRMS (ESI) *m*/*z* calcd for C_37_H_29_O_3_ [M – H]^−^ 521.2117, found 521.2119.

#### Selenylation of **2a**

According to the modified procedure,^55^ diisopropylamine (91 μL, 2.20 equiv) was dissolved in dry tetrahydrofuran (5 mL). After the mixture had cooled to 0 °C, *n*-butyllithium (260 μL, 2.20 equiv, 2.5 M solution in *n*-hexane) was added and the reaction mixture was stirred at room temperature for 30 min. Then after the mixture had again cooled to 0 °C, diketone **2a** (100 mg, 0.29 mmol, 1 equiv) was dissolved in dry tetrahydrofuran (3 mL) and slowly added to the solution of diisopropylamine and *n*-butyllithium. The reaction mixture was stirred for 15 min followed by an addition of *N*-(phenylseleno)phthalimide (120 mg, 1.20 equiv); the subsequent mixture was stirred at 0 °C for 30 min and then at rt for an additional 60 min when, according to TLC, full conversion was achieved. The reaction was then quenched by the addition of a saturated aqueous solution of NH_4_Cl (1 mL) and water (10 mL), and EtAOc (20 mL) was then added. The organic phase was separated, and the aqueous phase extracted with EtOAc (2 × 20 mL). The combined organic phases were washed with brine and dried over MgSO_4_, and the solvent was evaporated in vacuo. The crude material was purified by automated column chromatography with a petroleum ether/EtOAc gradient (98:2 to 92:8), yielding product **11** as single diastereoisomer in the form of a thick yellow oil.

#### (2*Z*)-2-[Hydroxy(phenyl)methylidene]-3,4-diphenyl-5-(phenylselanyl)cyclopentan-1-one (**11**)

Yield 110 mg (76%) as a thick yellow oil; 1:20 keto/enol. Enol: ^1^H NMR (400 MHz, CDCl_3_) δ 7.62–7.53 (m, 2H), 7.52–7.49 (m, 2H), 7.39–7.26 (m, 2H), 7.28–7.18 (m, 4H), 7.14–7.01 (m, 6H), 6.75–6.68 (m, 2H), 6.67–6.59 (m, 2H), 4.42 (d, *J* = 12.1 Hz, 1H), 4.33 (d, *J* = 7.6 Hz, 1H), 3.66 (dd, *J* = 12.1 Hz, *J*′ = 7.6 Hz, 1H); ^13^C{^1^H} NMR (101 MHz, CDCl_3_) δ 205.2, 172.3, 140.2, 137.6, 136.50 (2C), 133.7, 131.5, 129.0 (2C), 128.6, 128.5 (6C), 128.3 (4C), 128.0 (2C), 126.9 (2C), 126.8, 111.6, 54.5, 51.3, 50.5. Keto: ^1^H NMR (400 MHz, CDCl_3_) δ 8.10–8.04 (m, 2H), 7.76 (dd, *J* = 8.2 Hz, *J*′ = 1.3 Hz, 1H), 7.68–7.61 (m, 4H), 7.35–7.28 (m, 2H), 7.26–7.20 (m, 2H), 7.18–7.12 (m, 3H), 7.08–7.04 (m, 2H), 6.80–6.75 (m, 4H), 4.86 (dd, *J* = 8.1 Hz, *J*′ = 1.2 Hz, 1H), 4.70 (t, *J* = 7.6 Hz, 1H), 4.29 (dd, *J* = 5.6 Hz, *J*′ = 1.3 Hz, 1H), 3.97–3.89 (m, 1H); ^13^C{^1^H} NMR (101 MHz, CDCl_3_) δ 206.9, 192.4, 136.5 (2C), 136.1 (2C), 133.7, 131.5, 130.0, 129.3, 129.0 (2C), 128.6 (2C), 128.5 (2C), 128.5 (4C), 128.3 (2C), 128.2 (2C), 127.1 (2C), 126.9, 60.6, 52.2, 48.91; ATR-FTIR ν 3059, 3028, 1719, 1604, 1592, 1568, 1493, 1360, 1260, 1221, 1150, 1071 cm^–1^; HRMS (ESI) *m*/*z* calcd for C_30_H_25_O_2_Se [M + H]^+^ 497.1014, found 497.1019.

#### Elimination of Brominated Diketo Compound **4**

1,4-Diazabicyclo[2.2.2]octane (DABCO) (0.49 mmol, 55 mg, 2 equiv) was added to a stirred solution of brominated diketone **4** (0.24 mmol, 100 mg, 1 equiv) in THF (5 mL) at rt. Full conversion was achieved after 2 h (TLC monitoring). The reaction mixture was evaporated and purified by automated column chromatography with a pentane/EtOAc gradient (9:1 to 5:1), yielding product **12** as a thick orange oil.

#### 5-Benzoyl-3,4-diphenylcyclopent-2-en-1-one (**12**)

Yield 70 mg (85%); >2:1 keto/enol. Keto: ^1^H NMR (400 MHz, CDCl_3_) δ 8.13–8.01 (m, 2H), 7.66–7.57 (m, 2H), 7.55–7.44 (m, 3H), 7.41–7.24 (m, 4H), 7.25–7.16 (m, 3H), 7.00–6.89 (m, 1H), 6.69 (d, *J* = 1.4 Hz, 1H), 5.34 (t, *J* = 1.7 Hz, 1H), 4.61 (d, *J* = 2.0 Hz, 1H); ^13^C{^1^H} NMR (101 MHz, CDCl_3_) δ 200.9, 193.2, 175.9, 141.3, 136.2, 133.7, 132.9, 131.4, 130.6, 130.2 (2C), 129.4 (2C), 128.9 (2C), 128.7 (2C), 128.4 (2C), 127.6 (2C), 126.5, 67.1, 50.4. Enol: ^1^H NMR (400 MHz, CDCl_3_) δ 8.12–8.08 (m, 2H), 7.60–7.57 (m, 2H), 7.55–7.50 (m, 2H), 7.41–7.24 (m, 5H), 7.24–7.18 (m, 2H), 7.00–6.91 (m, 2H), 6.87 (d, *J* = 1.2 Hz, 0H), 5.26 (d, *J* = 1.3 Hz, 1H); ^13^C{^1^H} NMR (101 MHz, CDCl_3_) δ 199.8, 169.9, 168.7, 138.8, 134.2, 133.2, 130.5, 128.8 (2C), 128.3 (4C), 128.1 (2C), 128.0 (2C), 127.9 (2C), 127.5 (2C), 126.8, 116.8, 50.8; ATR-FTIR ν 3060, 1690, 1666, 1596, 1570, 1493, 1446, 1176, 1148, 1000 cm^–1^; HRMS (ESI) *m*/*z* calcd for C_24_H_19_O_2_ [M + H]^+^ 339.1380, found 339.1380.

### General Procedure for Sample Preparation and Crystal Measurement

Single crystals of products **2a**, **4**, and **5** were prepared by volatilization using a mixture of methanol and dichloromethane as a solvent. Suitable crystals were selected and collected on a Bruker D8 VENTURE Kappa Duo PHOTONIII instrument by an IμS microfocus sealed tube with Mo Kα (λ = 0.71073) (**5**) or Cu Kα (λ = 1.54178 Å) (**2a** and **4**) radiation at a low temperature of 120 K.

## Supporting Information Available

NMR spectra for all new compounds (PDF)

## Accession Codes

CCDC 2063174–2063176 contain the supplementary crystallographic data for this paper. These data can be obtained free of charge via www.ccdc.cam.ac.uk/data_request/cif, or by emailing data_request@ccdc.cam.ac.uk, or by contacting The Cambridge Crystallographic Data Centre, 12 Union Road, Cambridge CB2 1EZ, UK; fax: +44 1223 336033.
