# Supplementary material for: Synthesis of cis-Oriented Vicinal Diphenylethylenes through a Lewis Acid-Promoted Annulation of Oxotriphenylhexanoates
Source: J Org Chem. 2021 Jun 17;86(13):8660–71. doi: 10.1021/acs.joc.1c00445 (PMC8279482; doi:10.1021/acs.joc.1c00445)
Supplement: Supplementary file 1 — jo1c00445_si_001.pdf [file jo1c00445_si_001.pdf]

Supplementary Information

Synthesis of *cis*-oriented *vicinal* diphenylethylenes through a Lewis acid promoted annulation of oxotriphenylhexanoates

Martin Kamlar,[a,b] Elin Henriksson,[a] Ivana Císařová,[c] Marcus Malo,[d] Henrik Sundén[d,*]

[a] Chalmers University of Technology, Department of Chemistry and Chemical Engineering, Kemivägen 10, 412 96, Gothenburg, Sweden.

[b] Department of Organic Chemistry, Faculty of Science, Charles University, Hlavova 2030/8, 128 43 Prague 2, Czech Republic.

[c] Department of Inorganic Chemistry, Faculty of Science, Charles University, Hlavova 2030/8, 128 43 Prague 2, Czech Republic, e-mail: cisarova@natur.cuni.cz.

[d] University of Gothenburg, Department of Chemistry and Molecular Biology, Kemivägen 10, SE-412 96, Gothenburg, Sweden, email:henrik.sunden@chem.gu.se.

Table of Contents

Suplementary Information 1

NMR data 2

X-ray data collection 34

NMR data

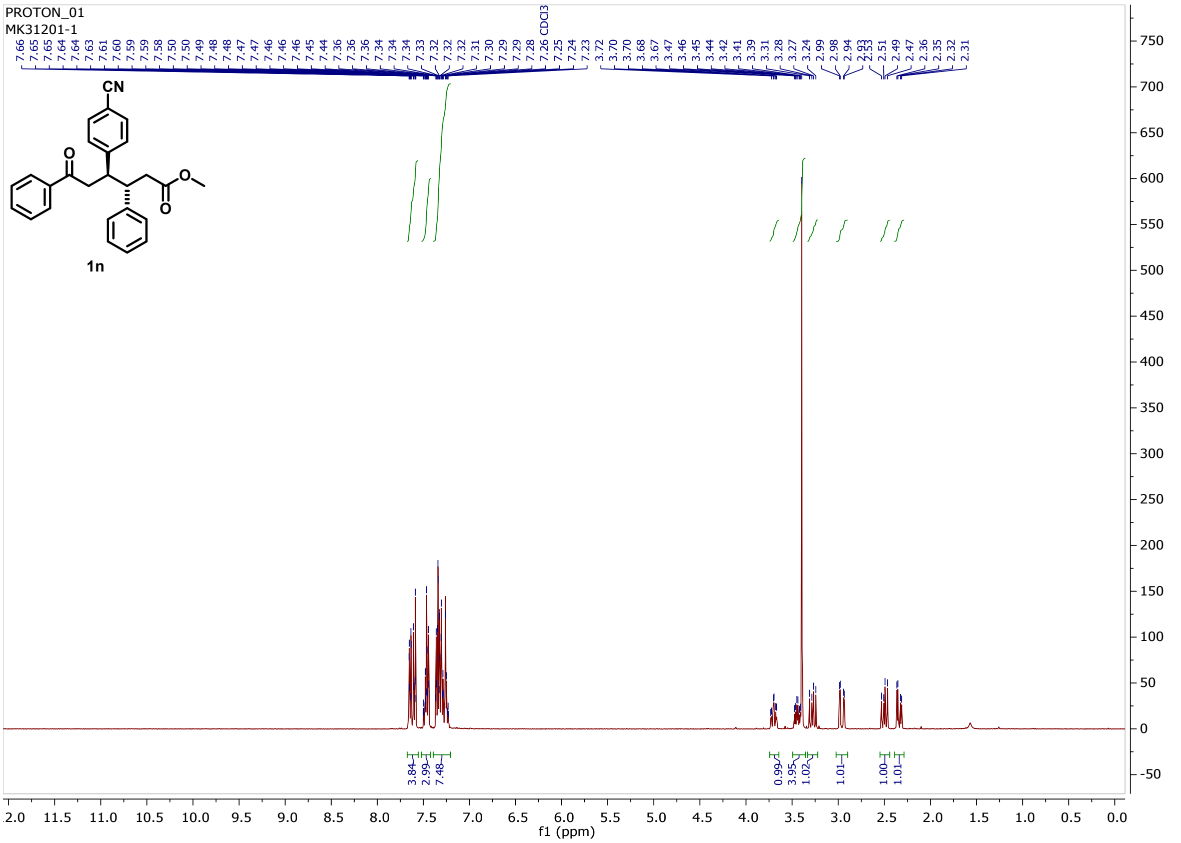

^{1}H NMR spectrum of **1n** ($CDCl_3$, 400 MHz).

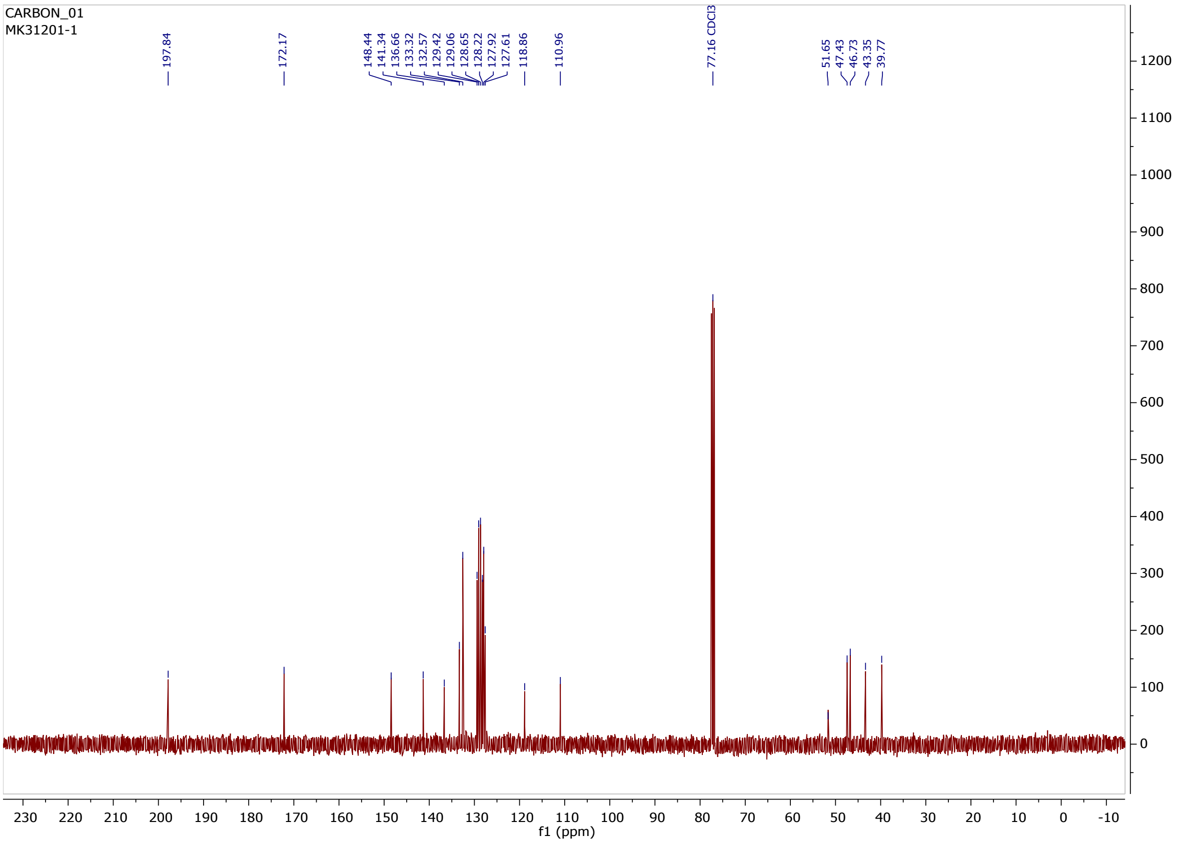

^{13}C {1H} NMR spectrum of **1n** ($CDCl_3$, 101 MHz).

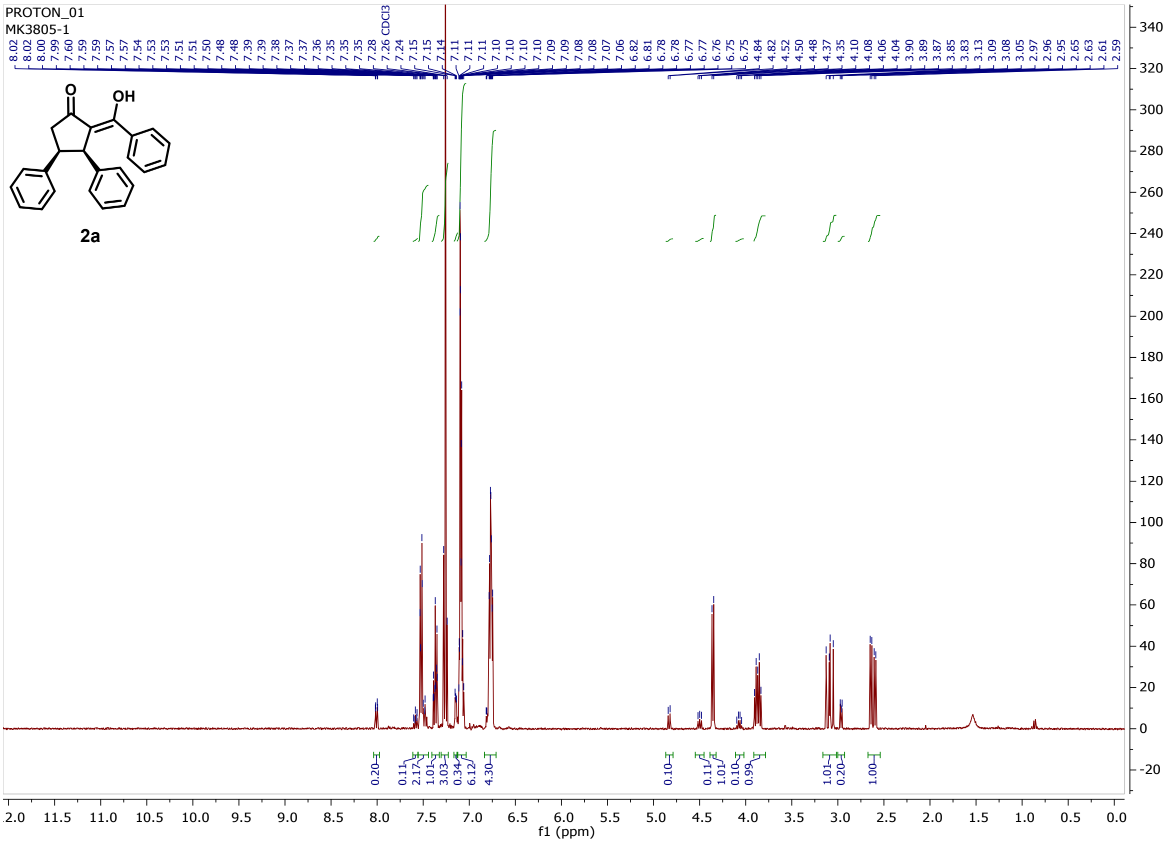

^{1}H NMR spectrum of **2a** ($CDCl_3$, 400 MHz).

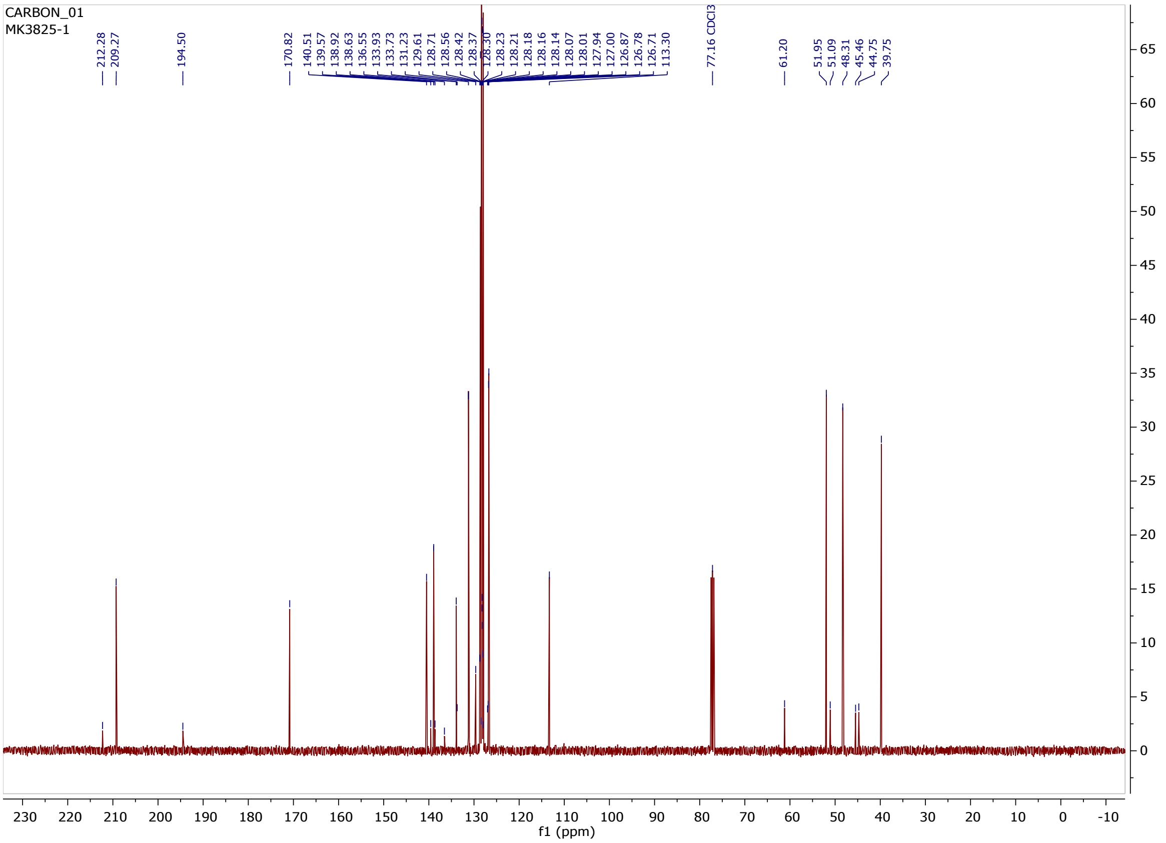

^{13}C {1H} NMR spectrum of **2a** ($CDCl_3$, 101 MHz).

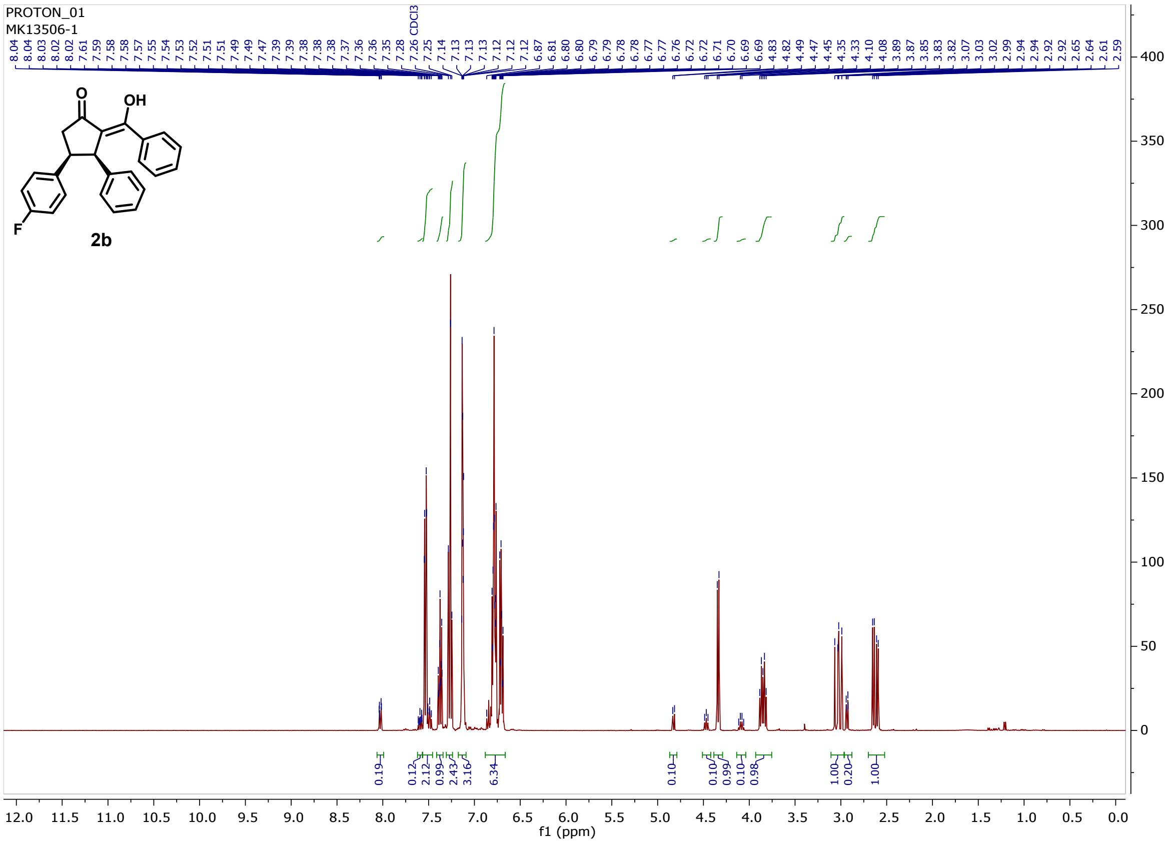

^{1}H NMR spectrum of **2b** ($CDCl_3$, 400 MHz).

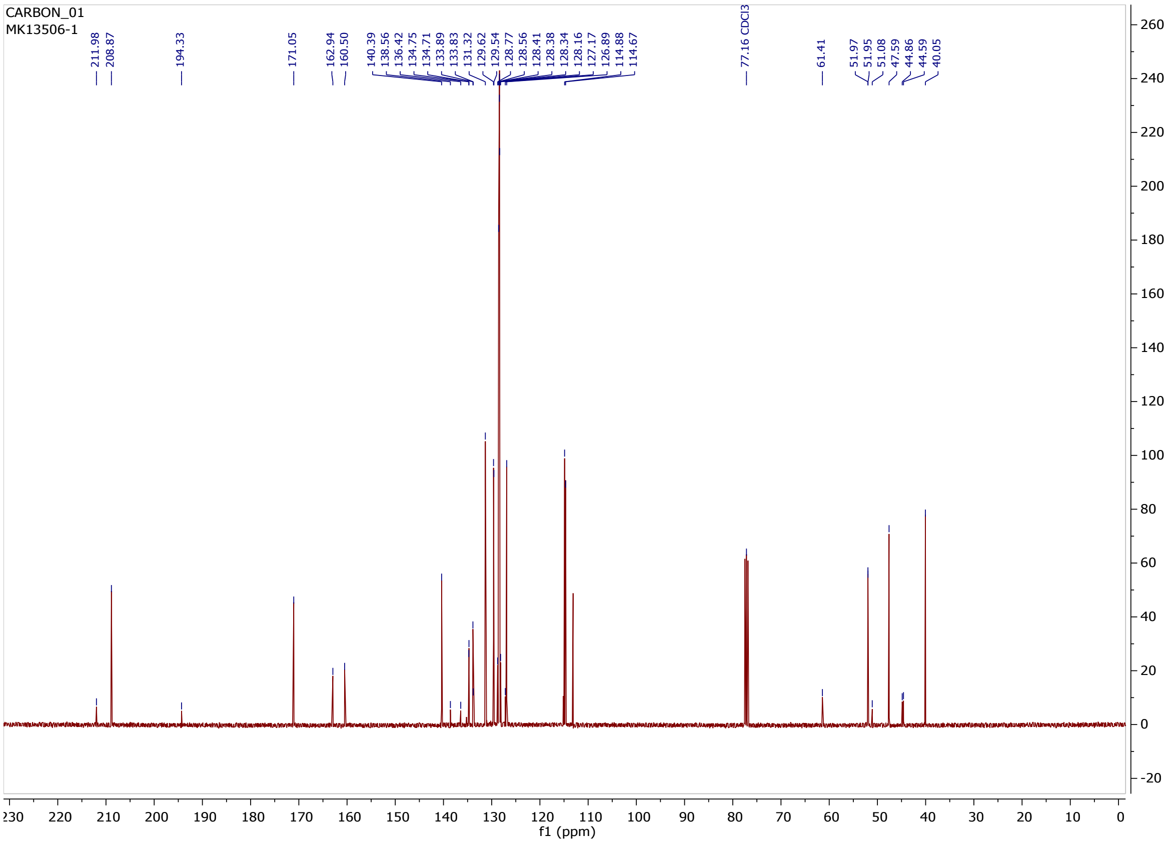

^{13}C {1H} NMR spectrum of **2a** ($CDCl_3$, 101 MHz).

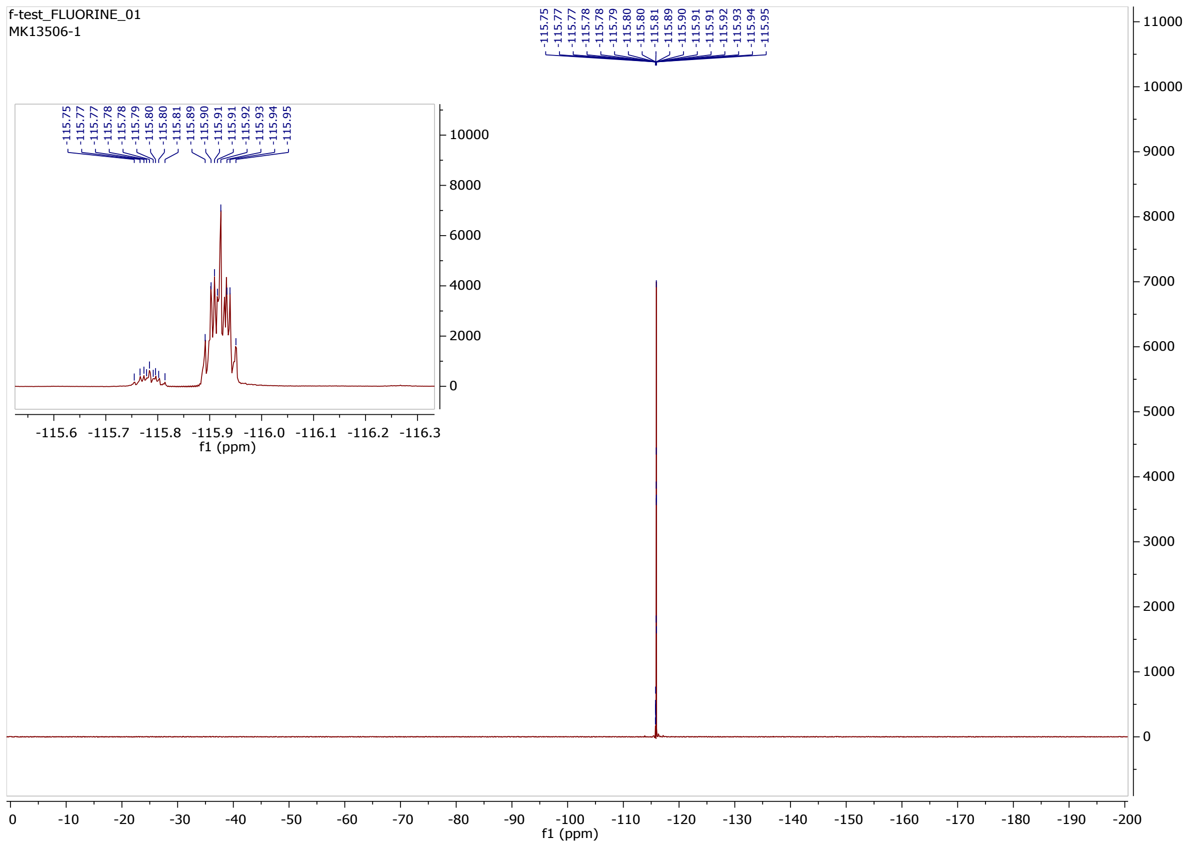

$^{19}F\{^{1}H\}$ NMR spectrum of **2b** ($CDCl_3$, 470 MHz).

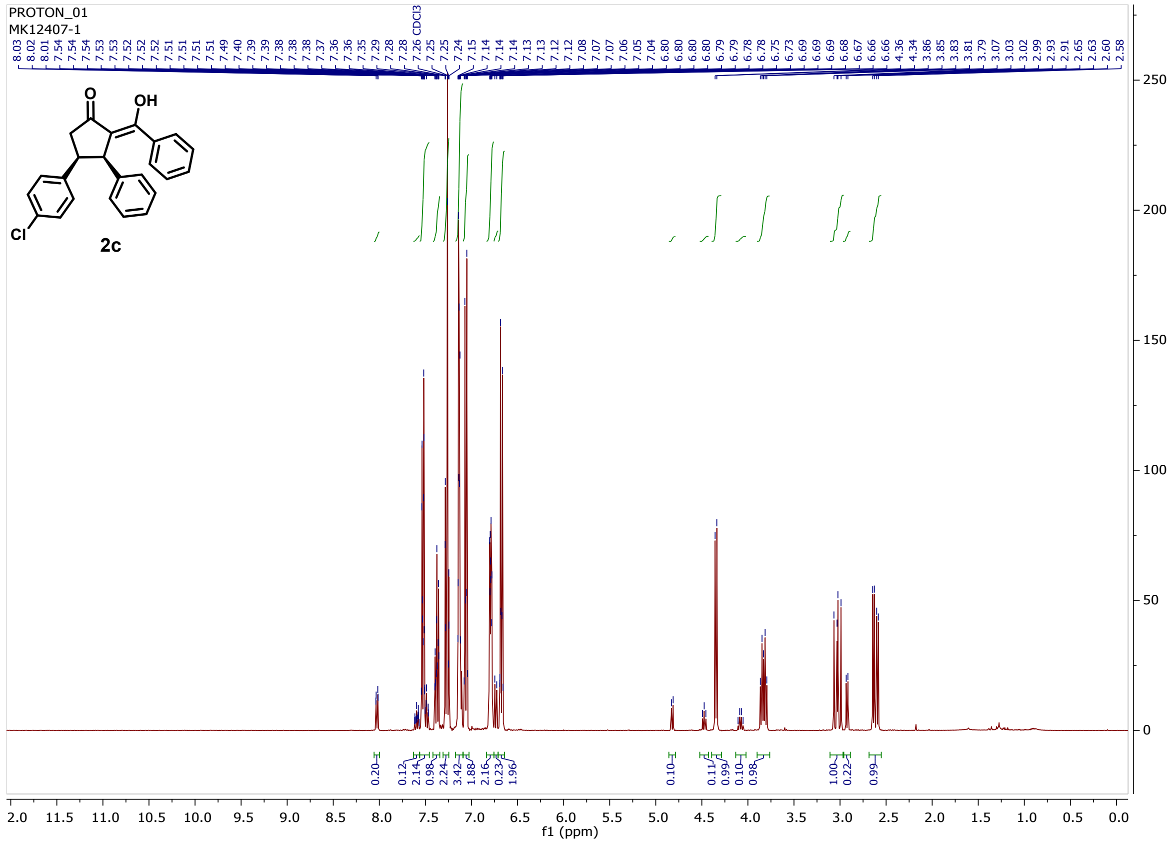

^{1}H NMR spectrum of **2c** ($CDCl_3$, 400 MHz).

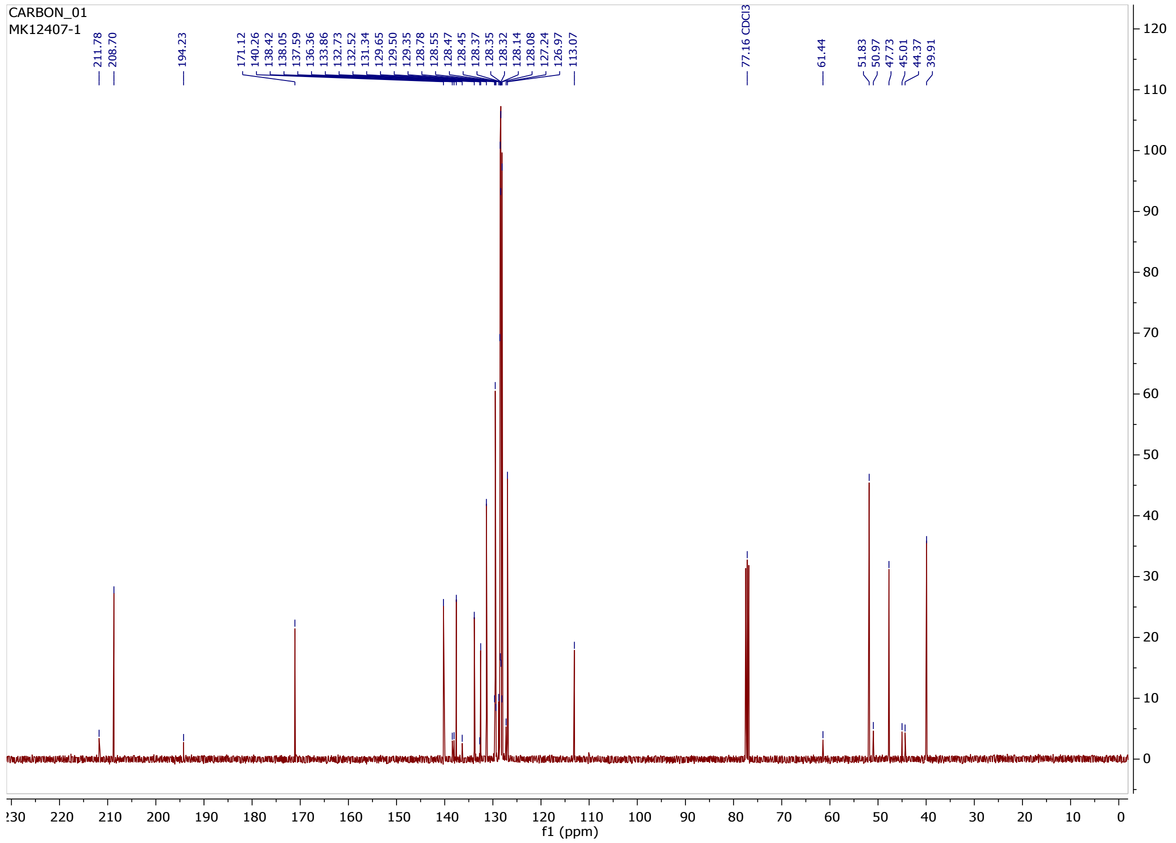

^{13}C {1H} NMR spectrum of **2c** ($CDCl_3$, 101 MHz).

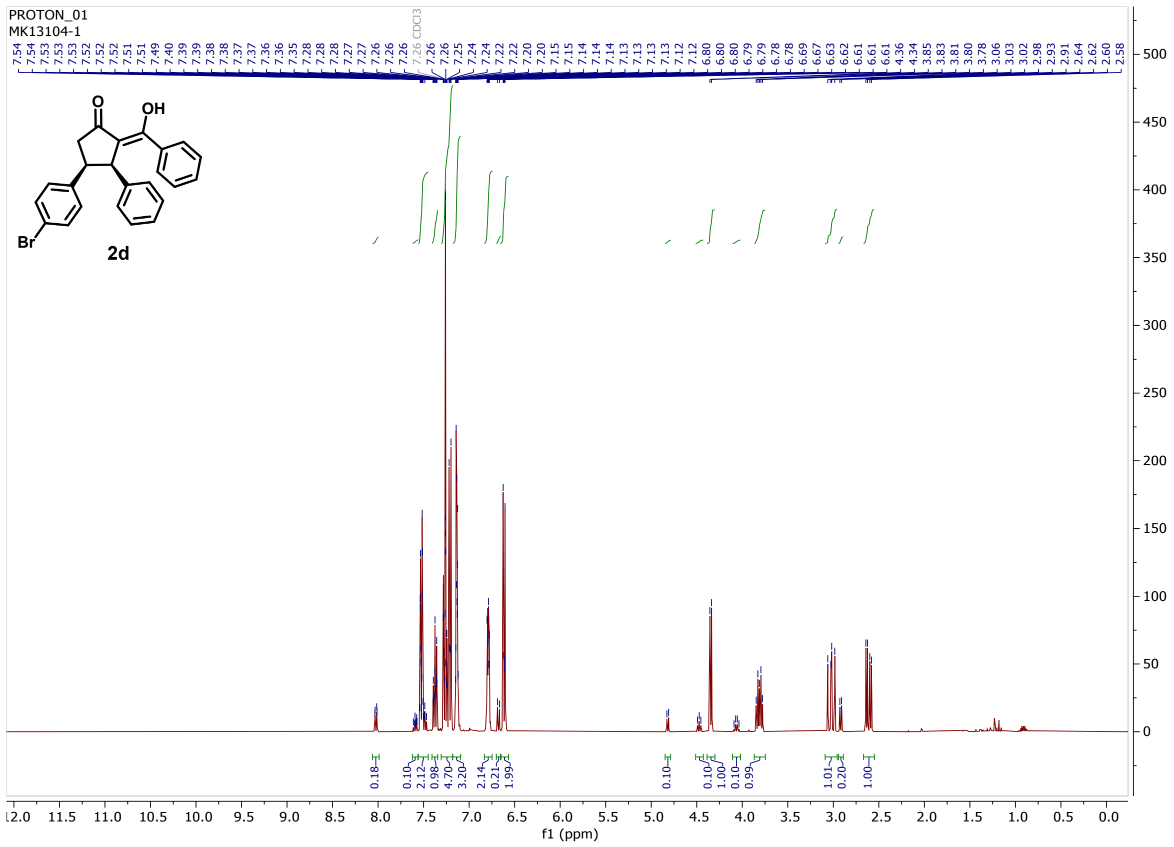

^{1}H NMR spectrum of **2d** ($CDCl_3$, 400 MHz).

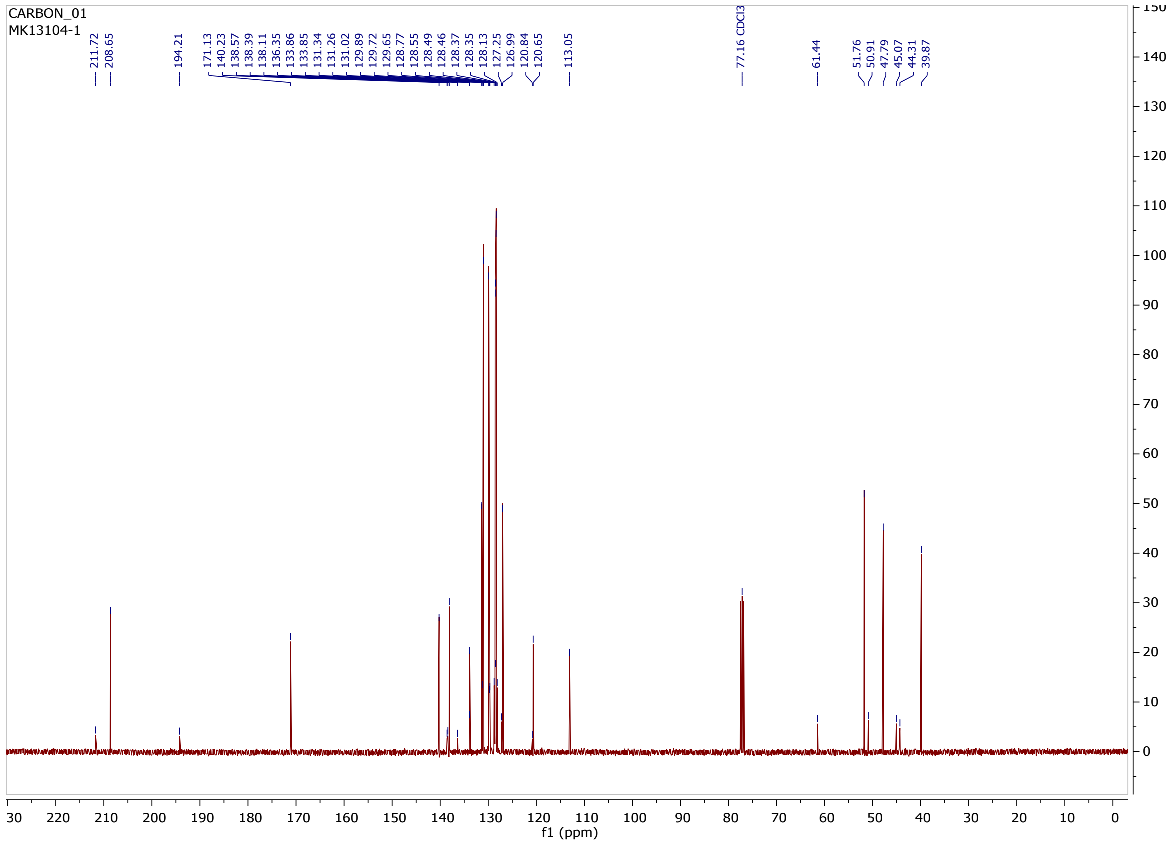

^{13}C {1H} NMR spectrum of **2d** ($CDCl_3$, 101 MHz).

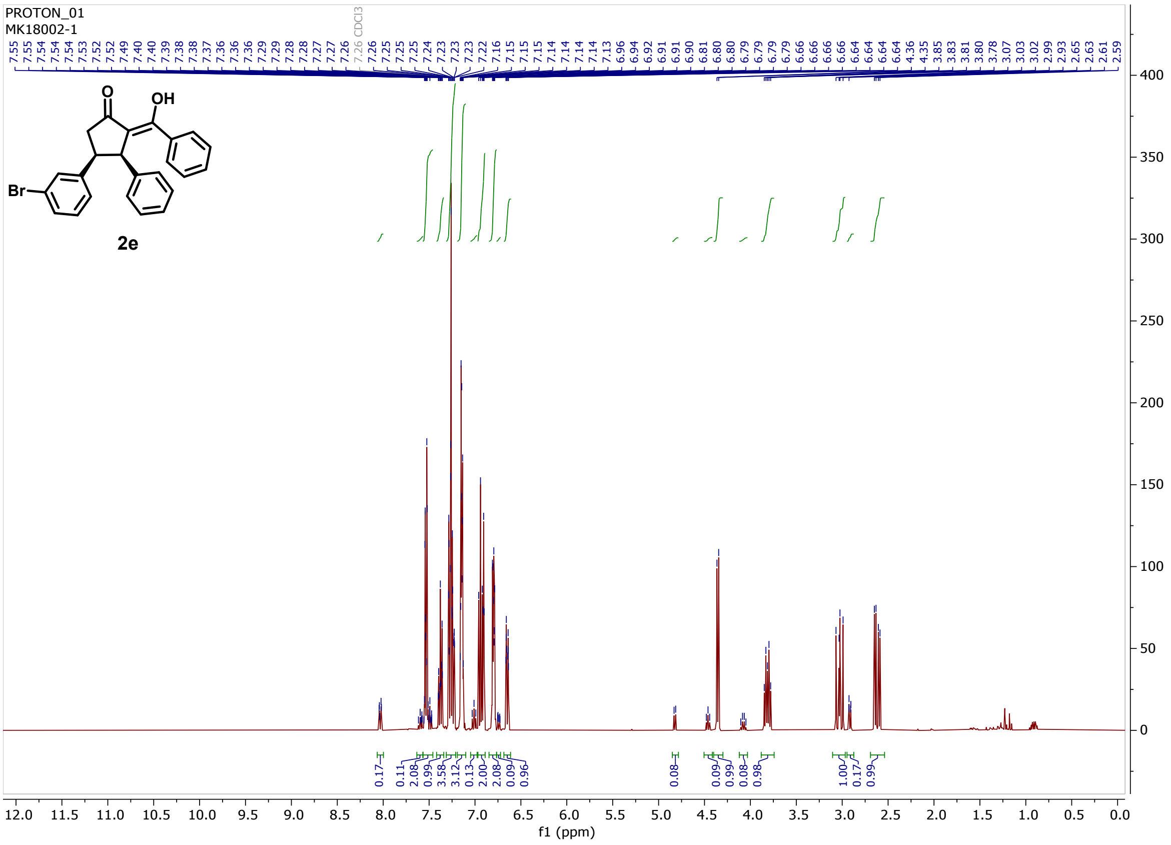

^{1}H NMR spectrum of **2e** ($CDCl_3$, 400 MHz).

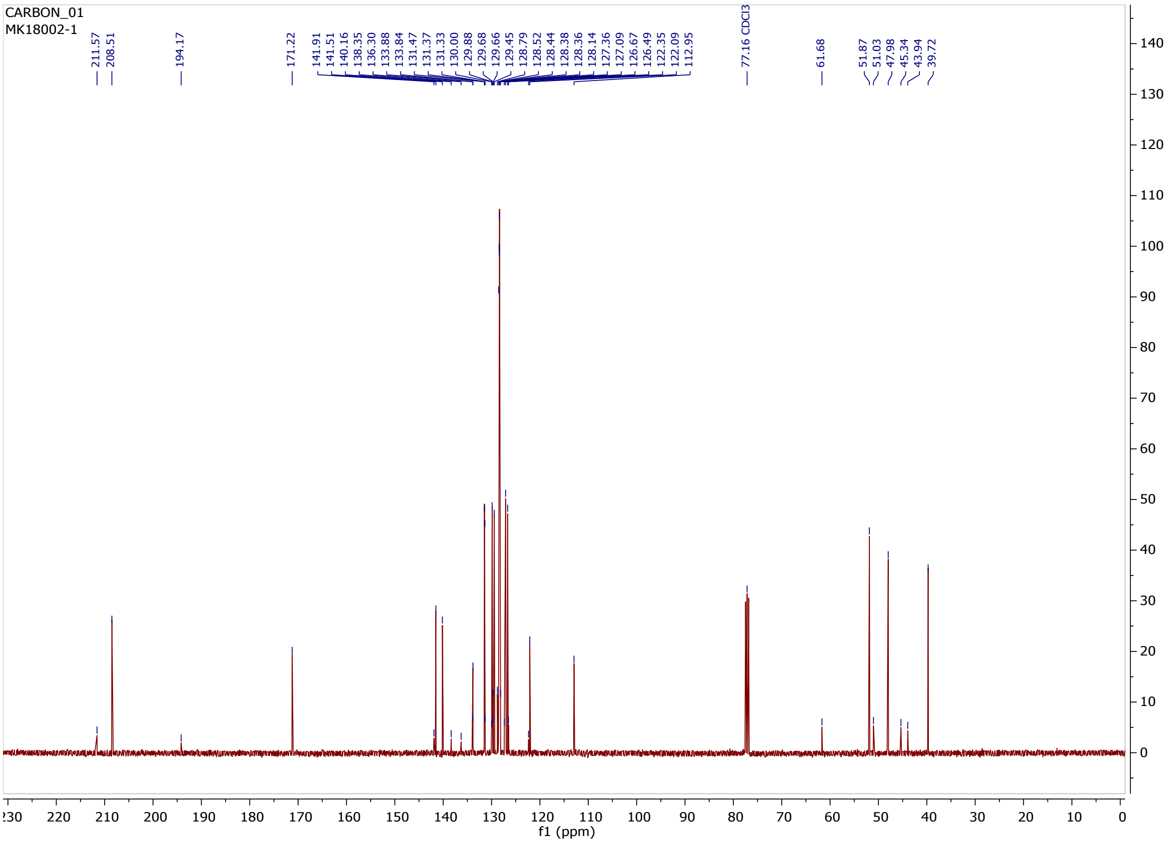

^{13}C {1H} NMR spectrum of **2e** ($CDCl_3$, 101 MHz).

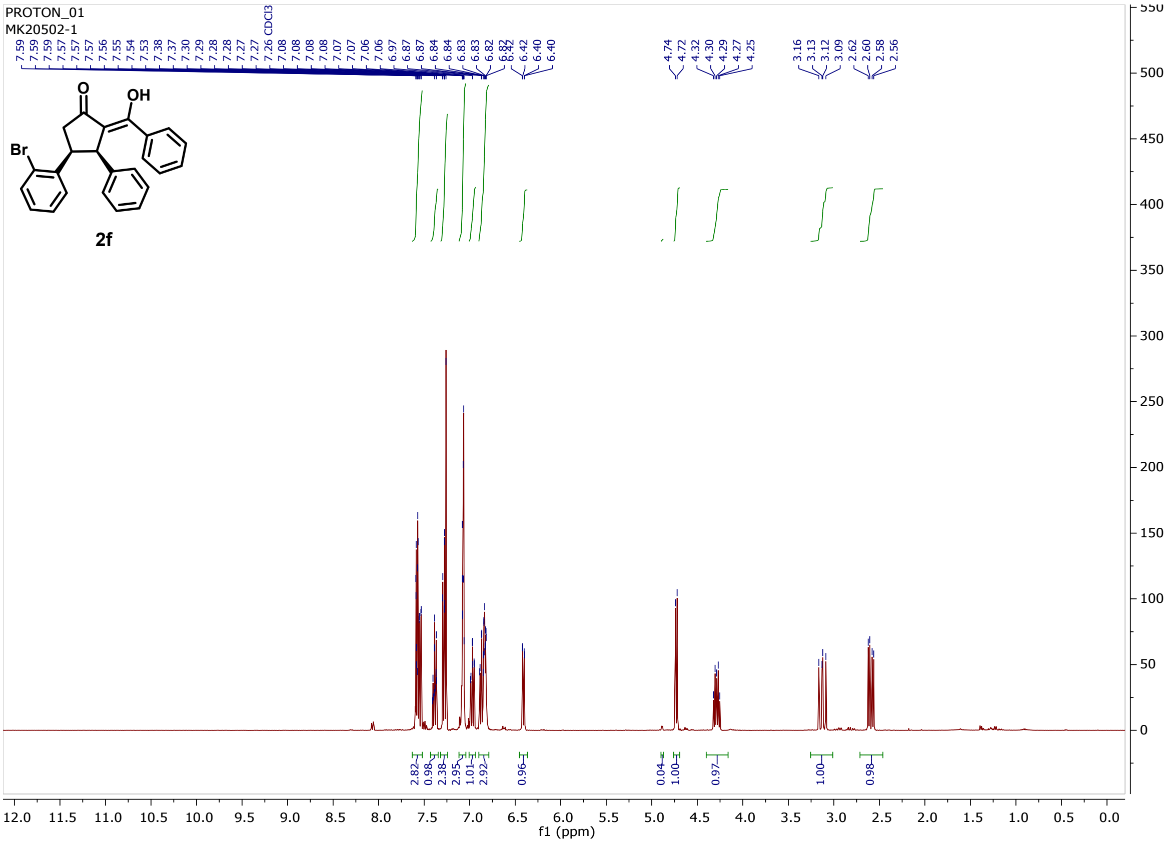

^{1}H NMR spectrum of **2f** ($CDCl_3$, 400 MHz).

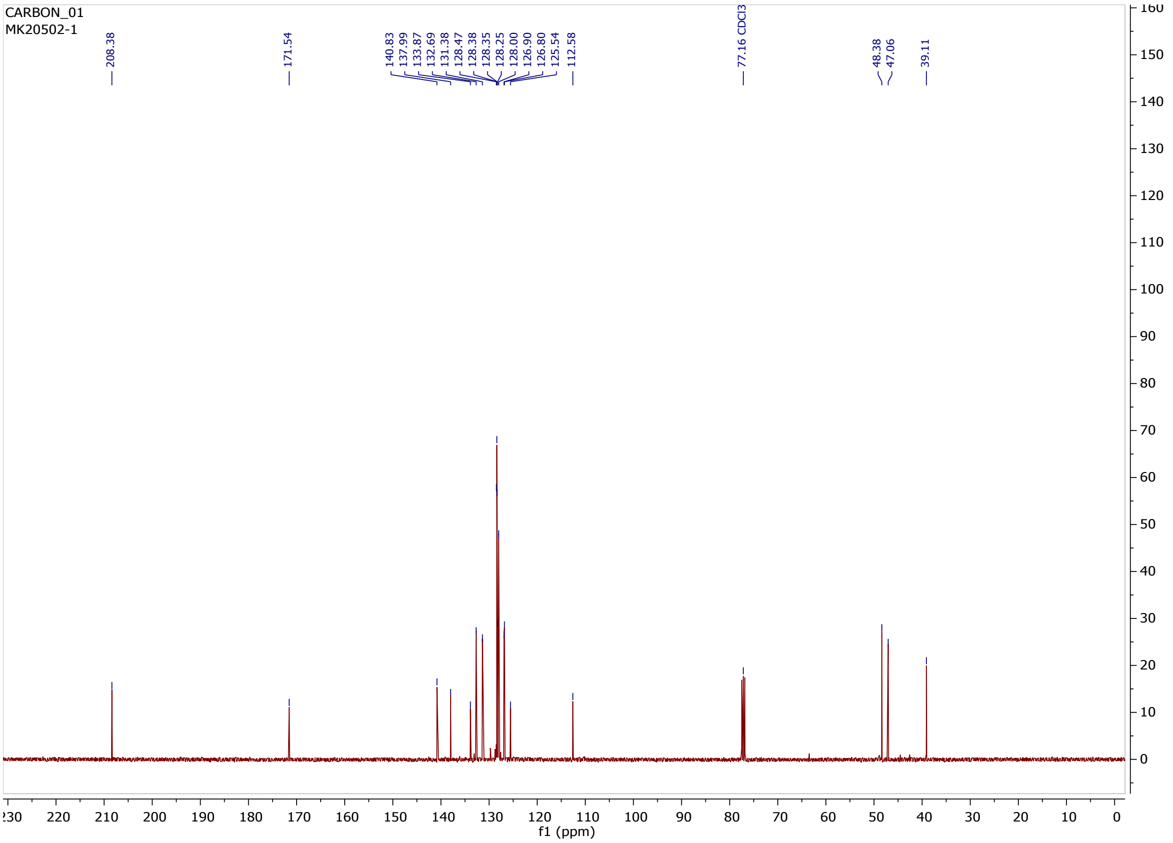

^{13}C {1H} NMR spectrum of **2f** ($CDCl_3$, 101 MHz).

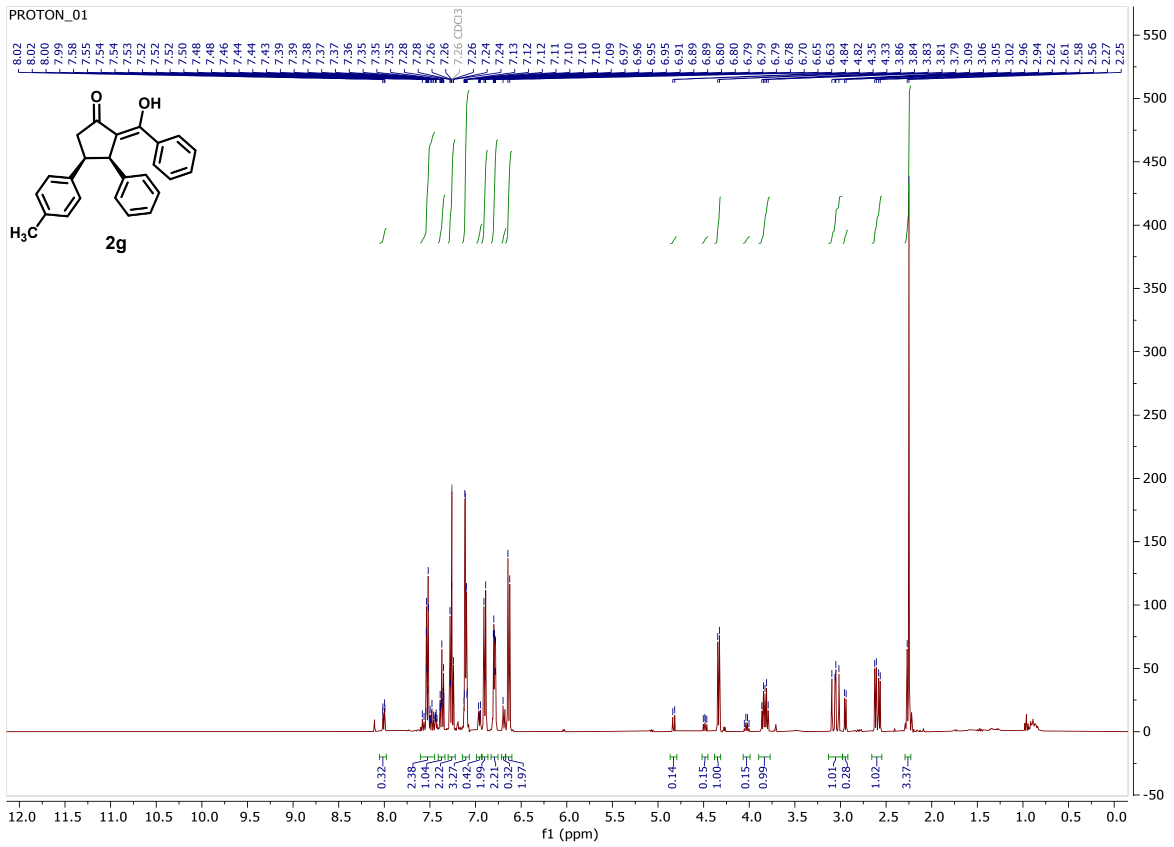

^{1}H NMR spectrum of **2g** ($CDCl_3$, 400 MHz).

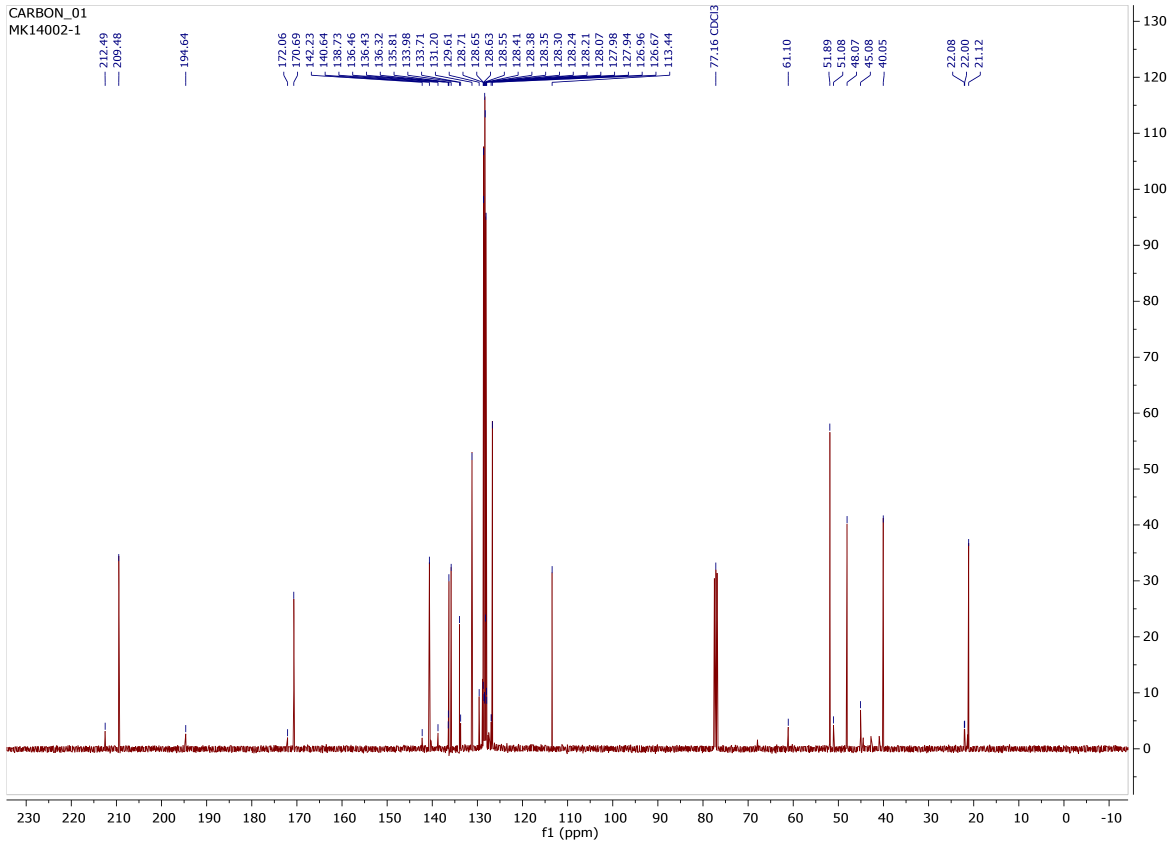

^{13}C {1H} NMR spectrum of **2g** ($CDCl_3$, 101 MHz).

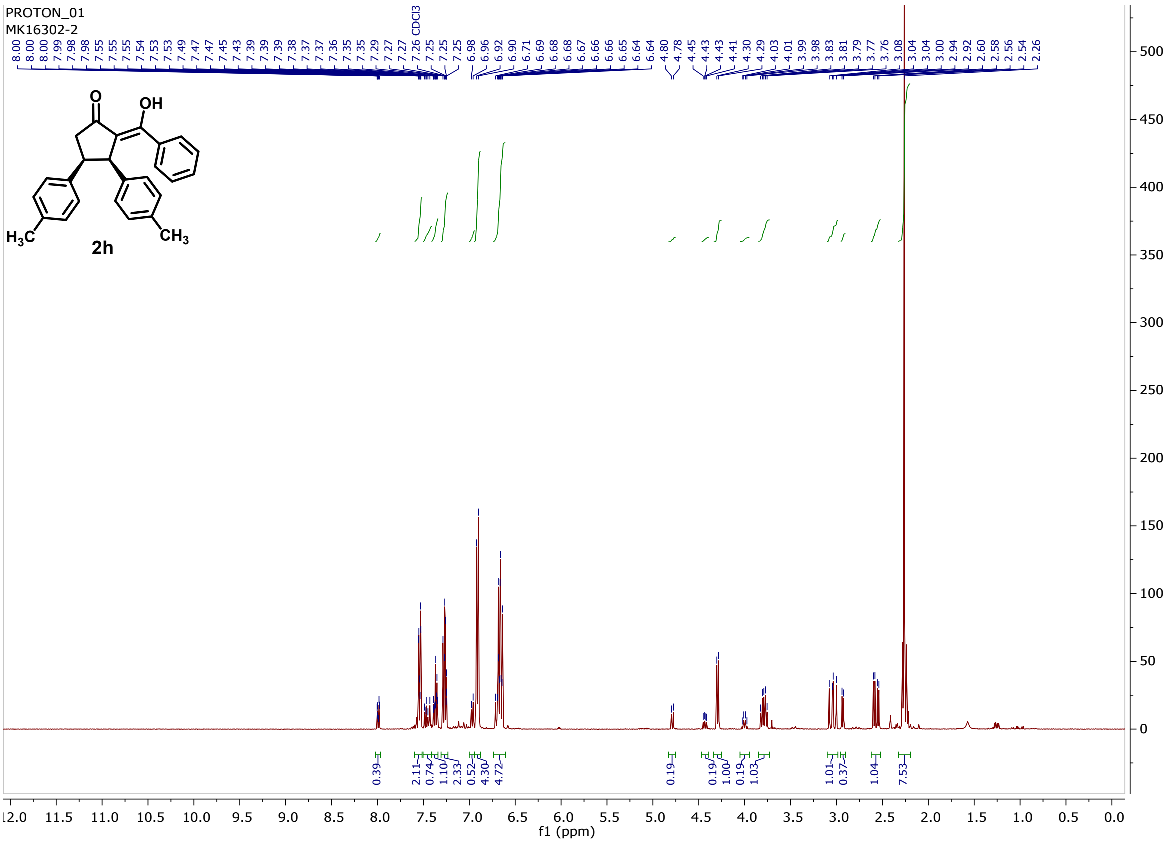

^{1}H NMR spectrum of **2h** ($CDCl_3$, 400 MHz).

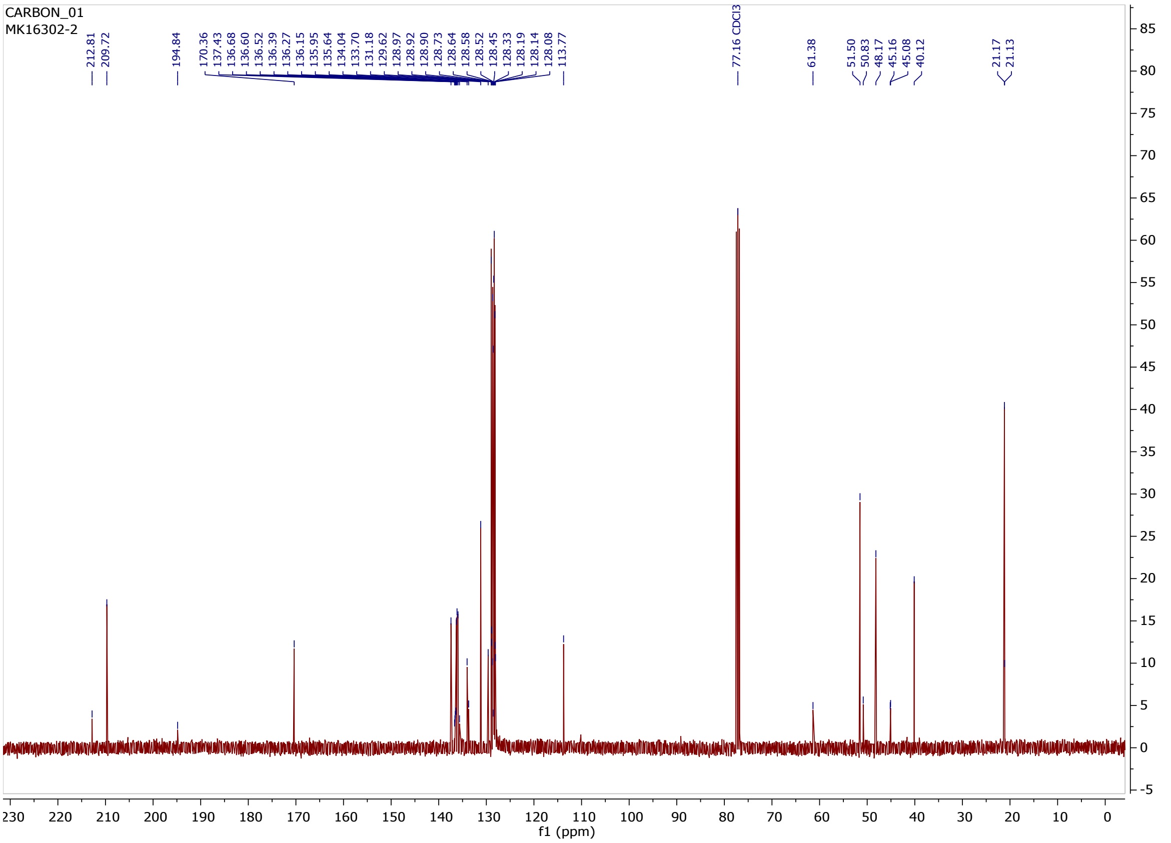

^{13}C {1H} NMR spectrum of **2h** ($CDCl_3$, 101 MHz).

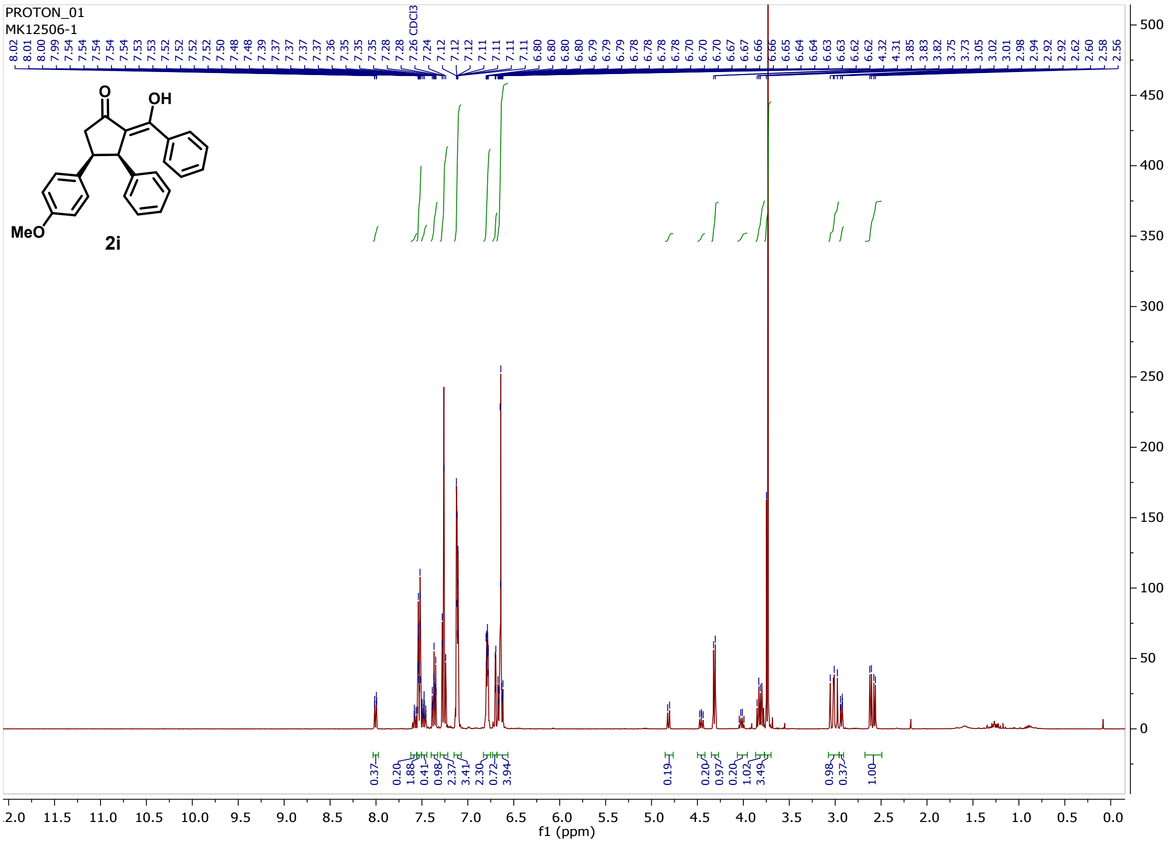

^{1}H NMR spectrum of **2i** ($CDCl_3$, 400 MHz).

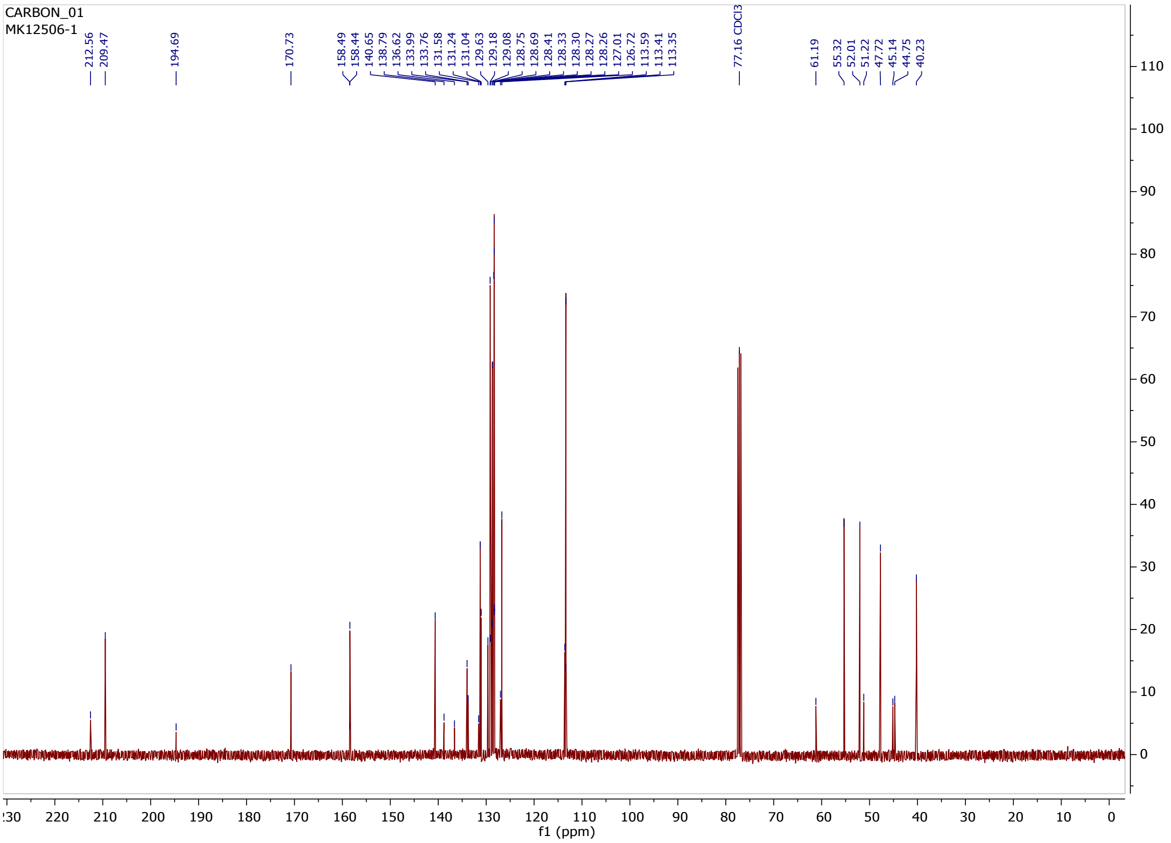

^{13}C {1H} NMR spectrum of **2i** ($CDCl_3$, 101 MHz).

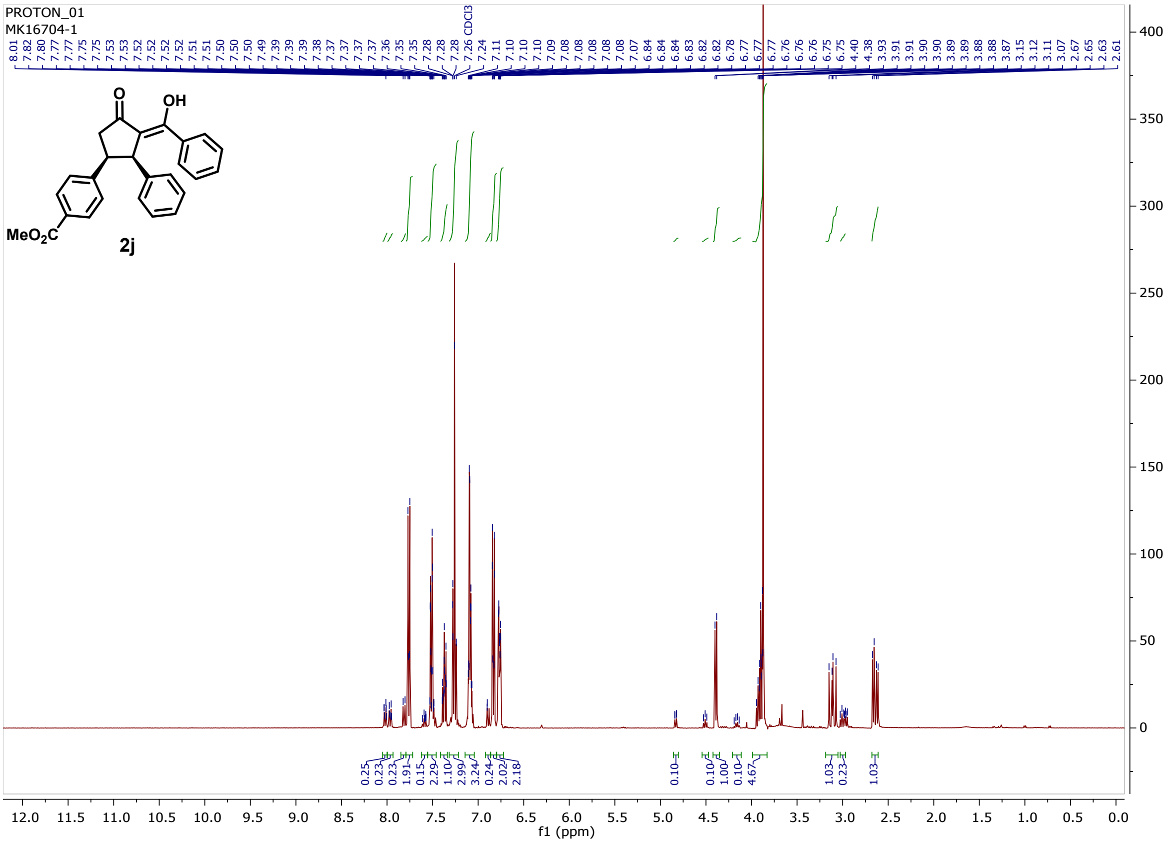

^{1}H NMR spectrum of **2j** ($CDCl_3$, 400 MHz).

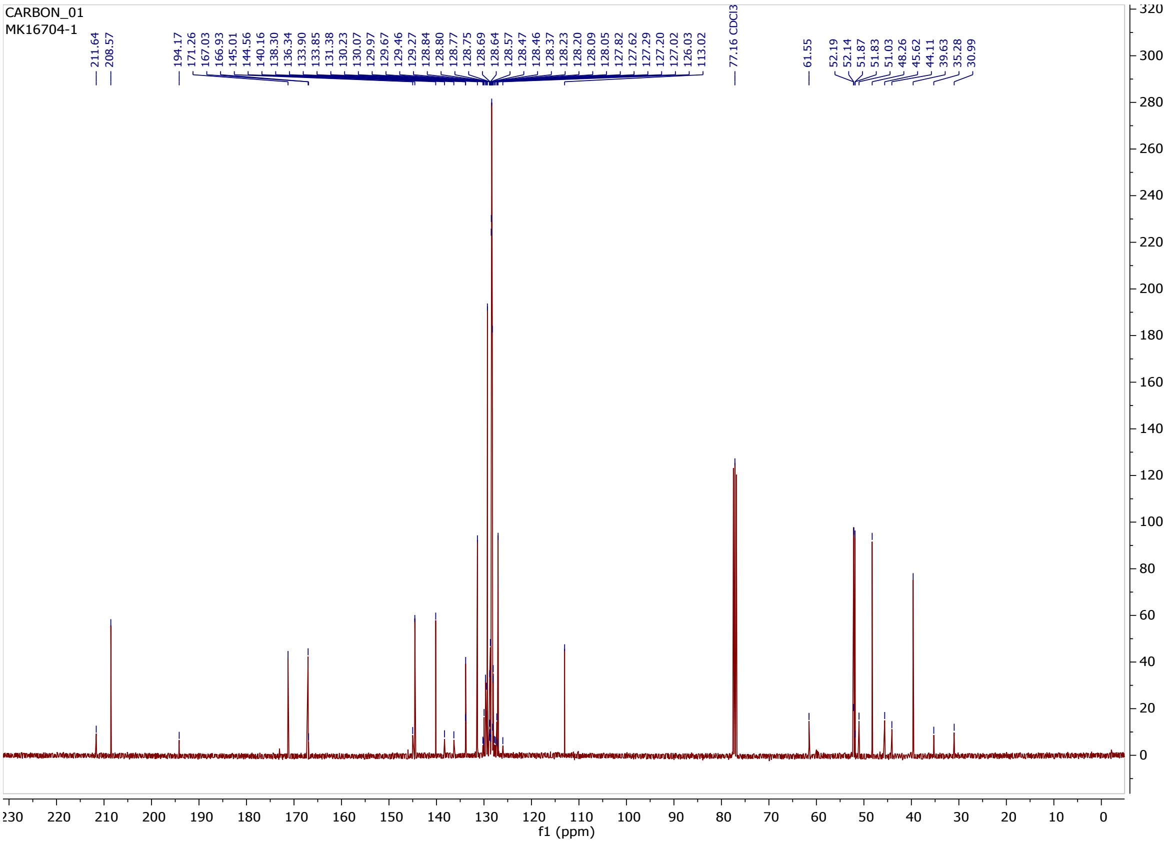

^{13}C {1H} NMR spectrum of **2j** ($CDCl_3$, 101 MHz).

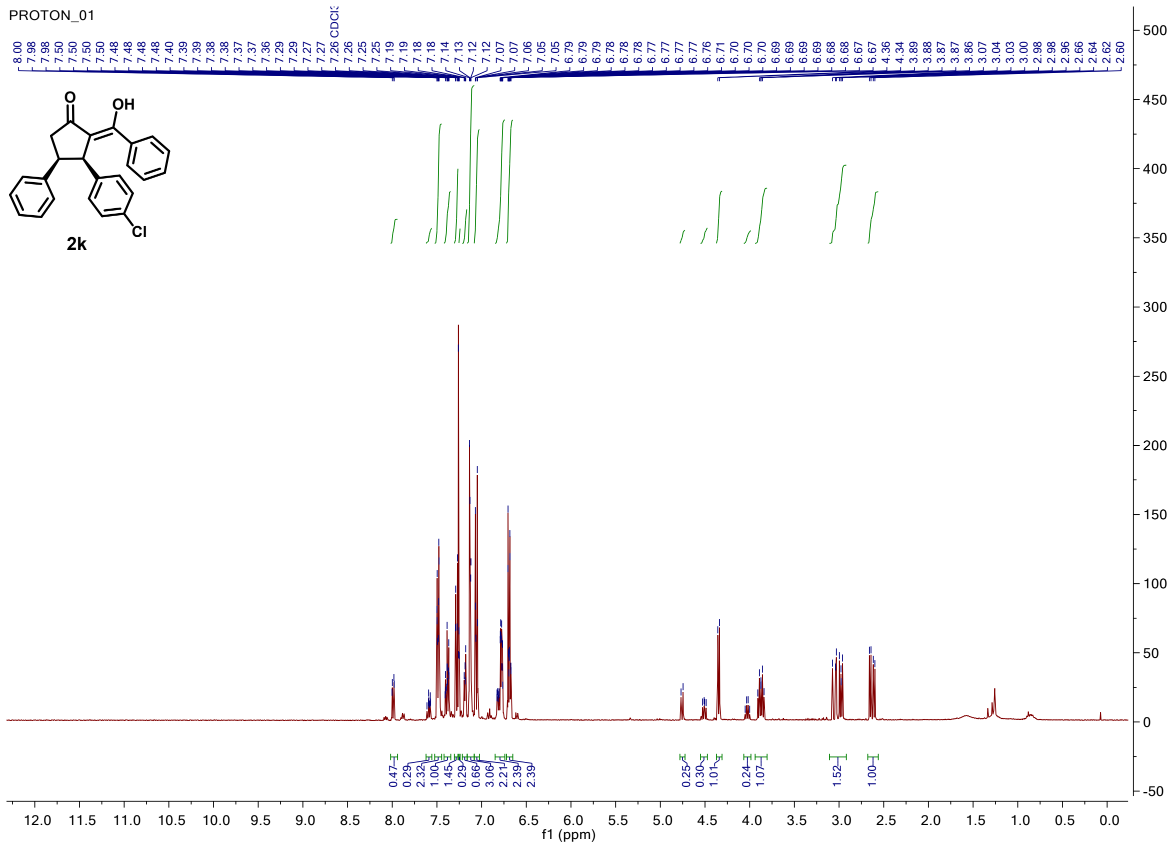

^{1}H NMR spectrum of **2k** ($CDCl_3$, 400 MHz).

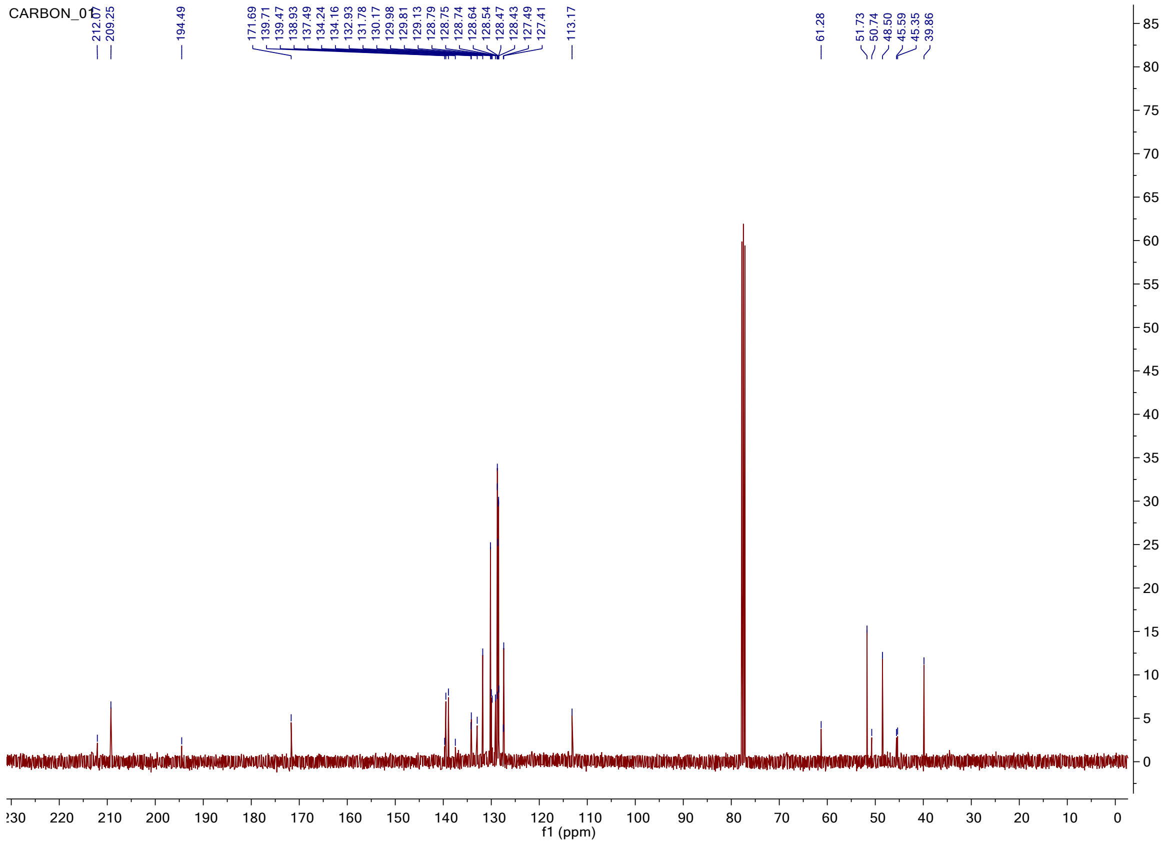

^{13}C {1H} NMR spectrum of **2k** ($CDCl_3$, 101 MHz).

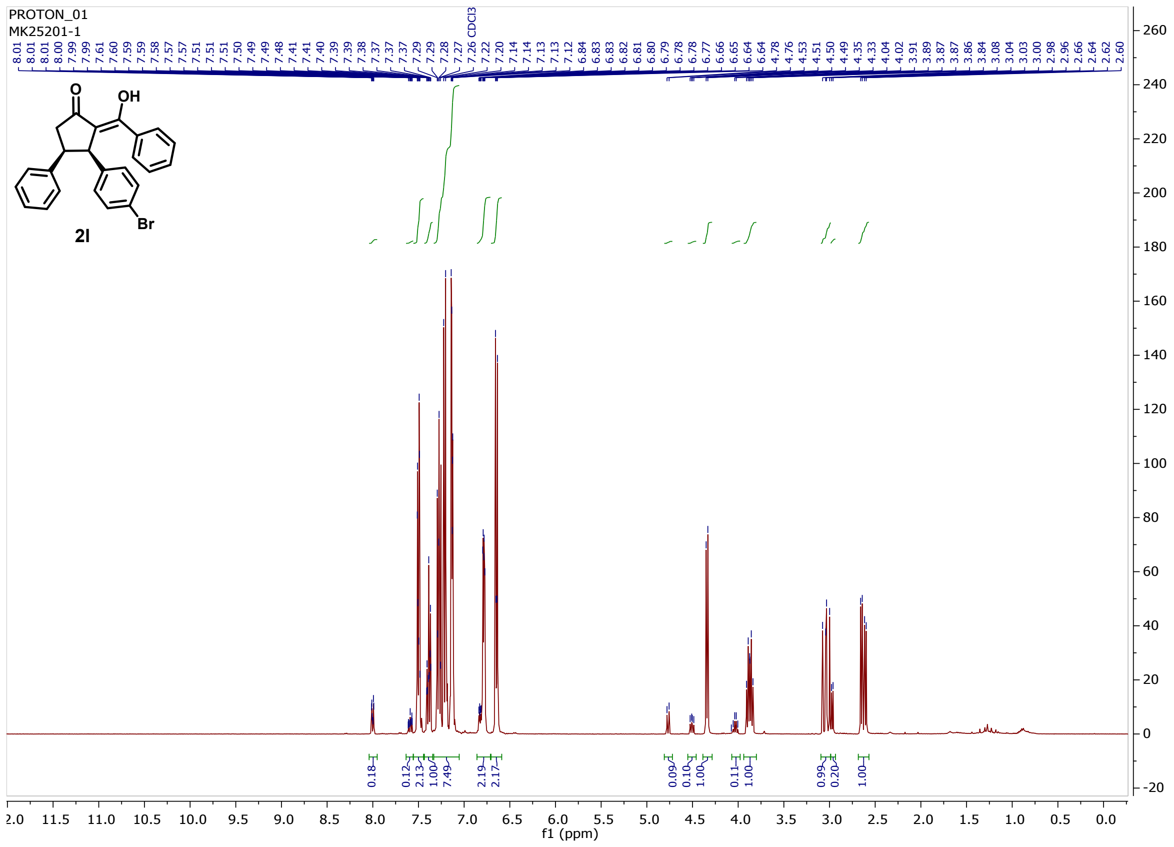

^{1}H NMR spectrum of **2l** ($CDCl_3$, 400 MHz).

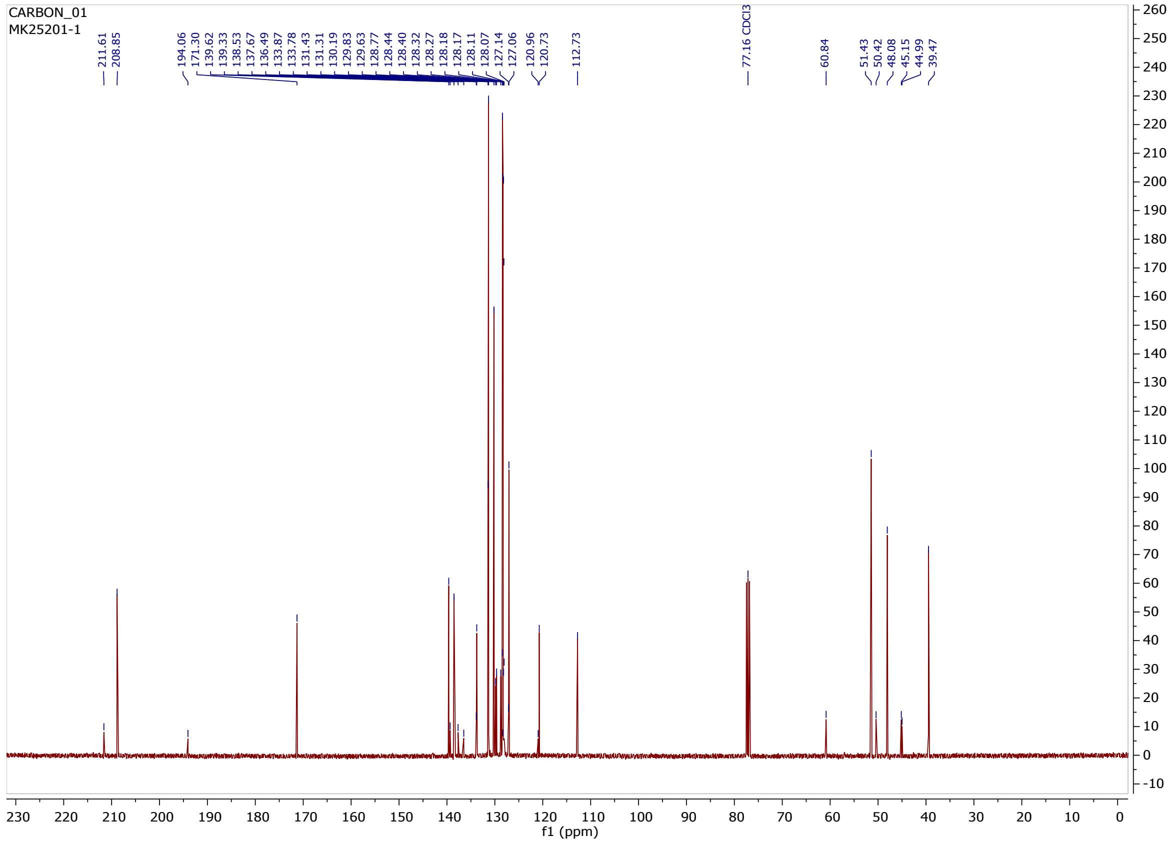

^{13}C {1H} NMR spectrum of **2l** ($CDCl_3$, 101 MHz).

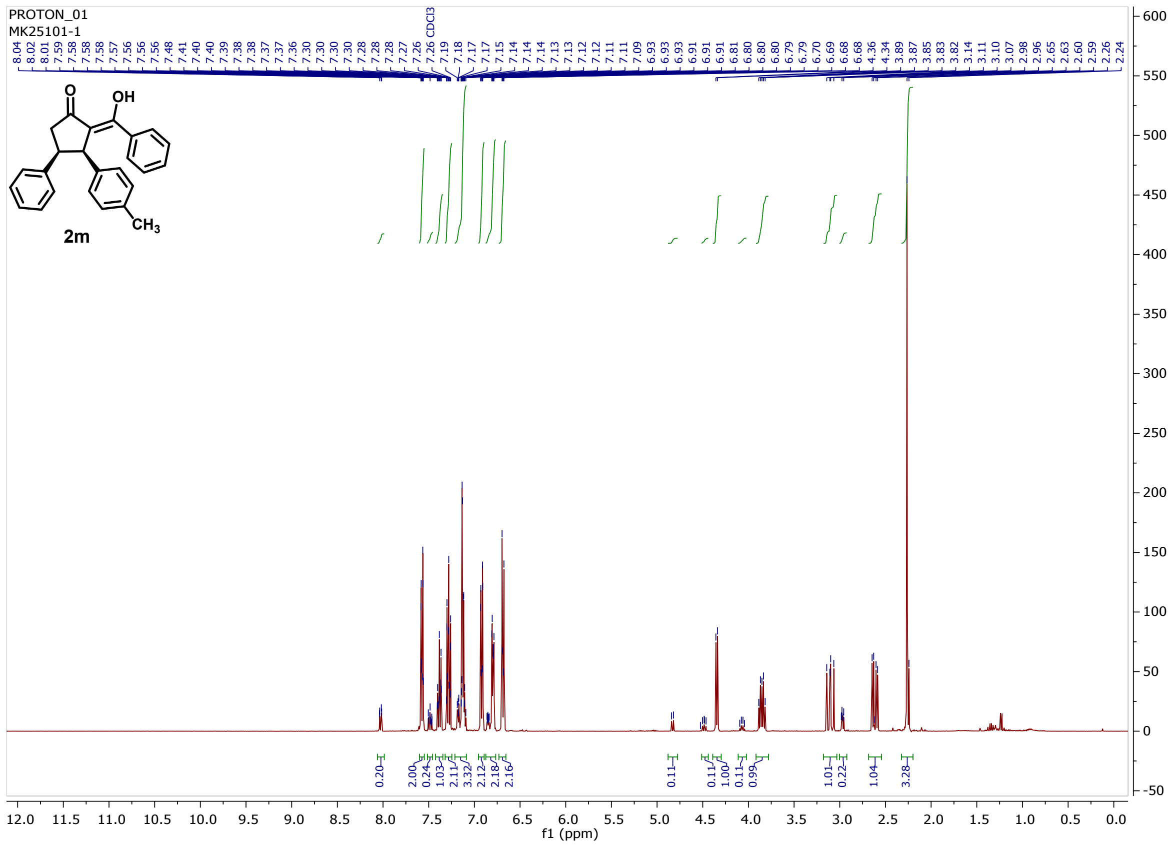

^{1}H NMR spectrum of **2m** ($CDCl_3$, 400 MHz).

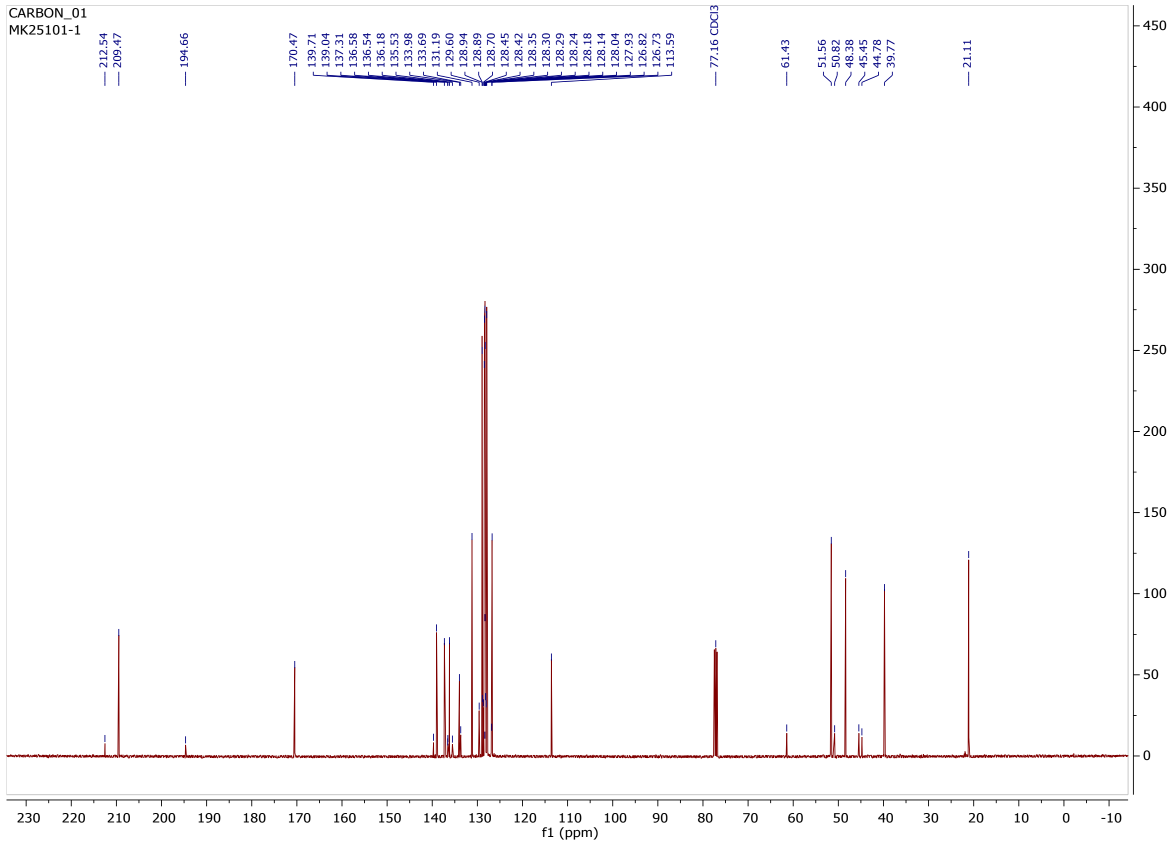

^{13}C {1H} NMR spectrum of **2m** ($CDCl_3$, 101 MHz).

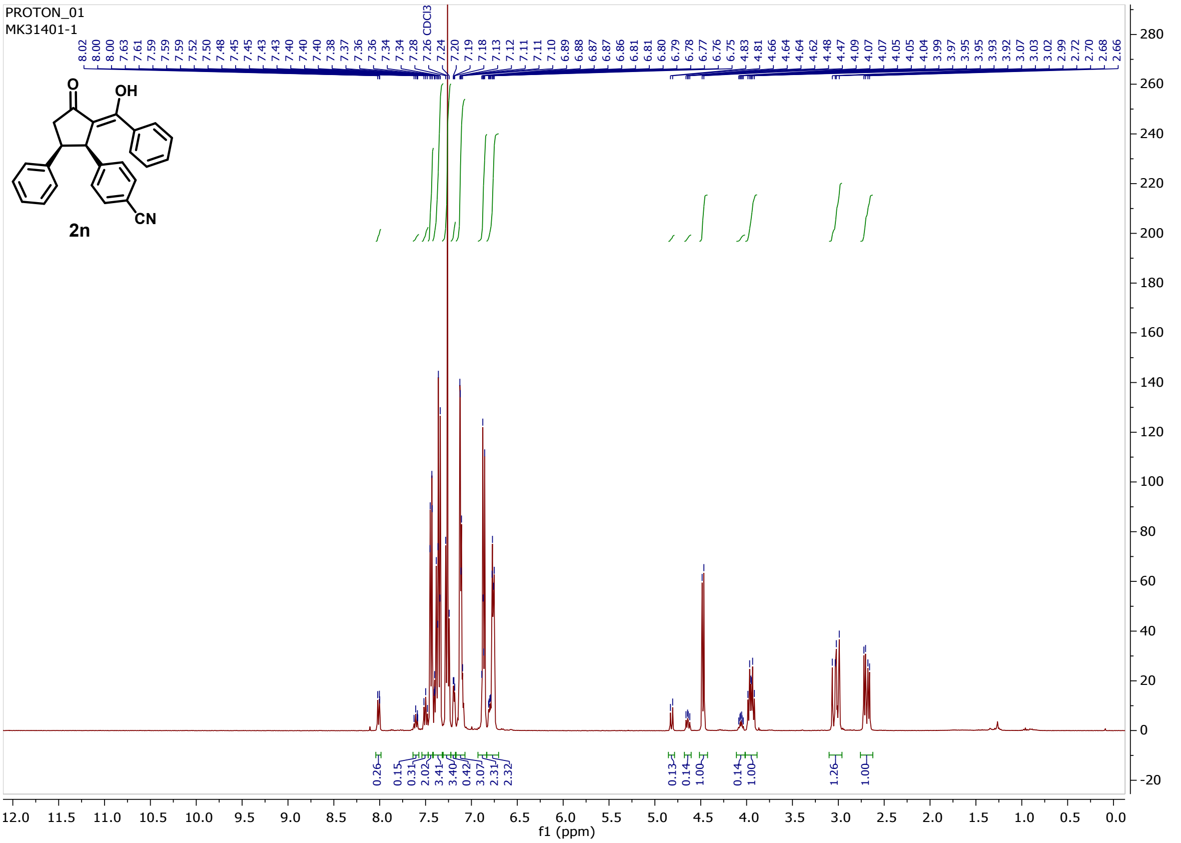

^{1}H NMR spectrum of **2n** ($CDCl_3$, 400 MHz).

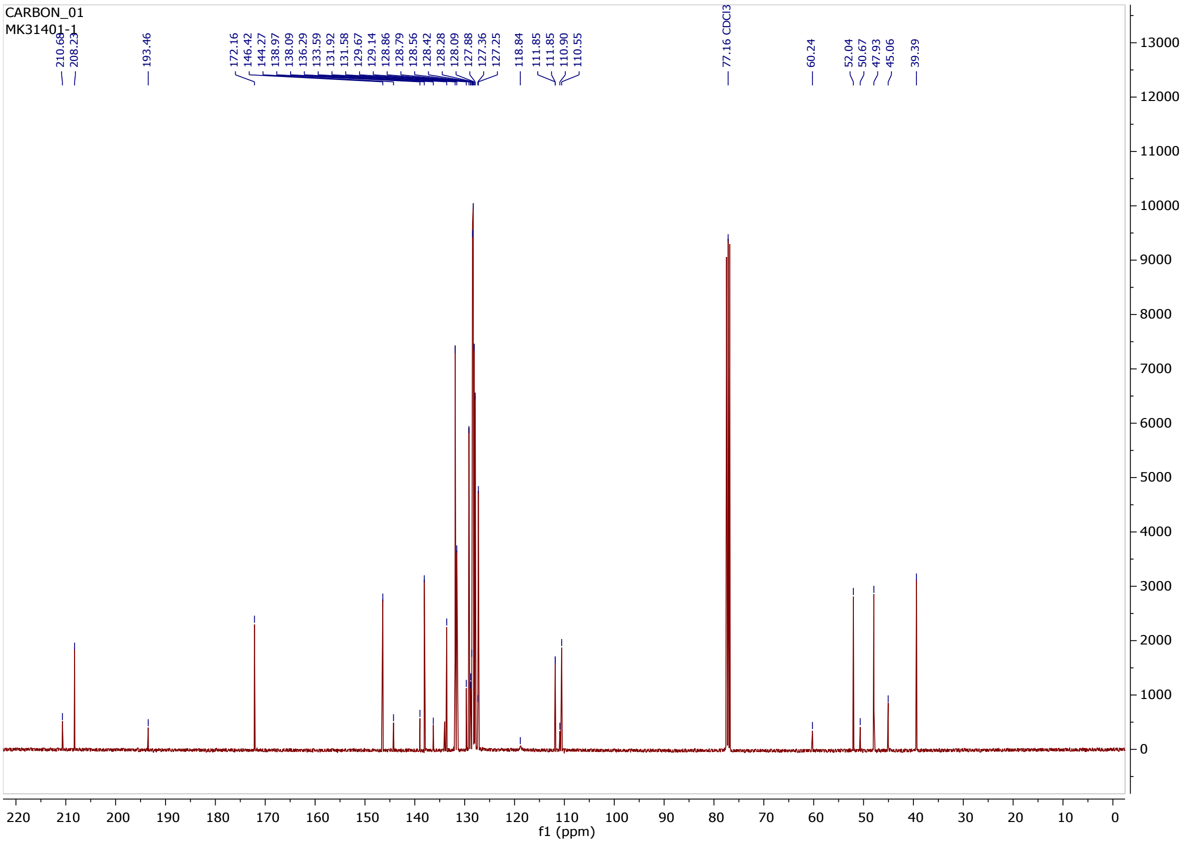

^{13}C {1H} NMR spectrum of **2n** ($CDCl_3$, 101 MHz).

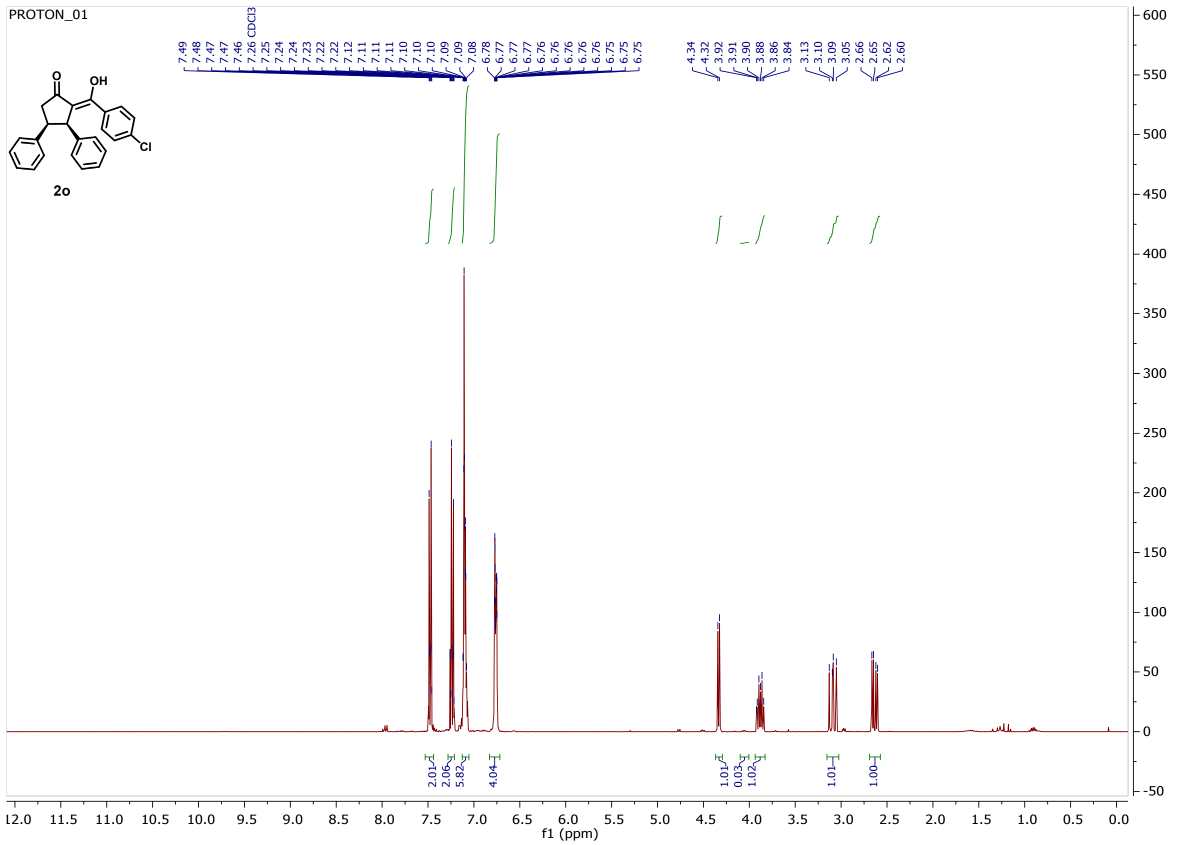

^{1}H NMR spectrum of **2o** ($CDCl_3$, 400 MHz).

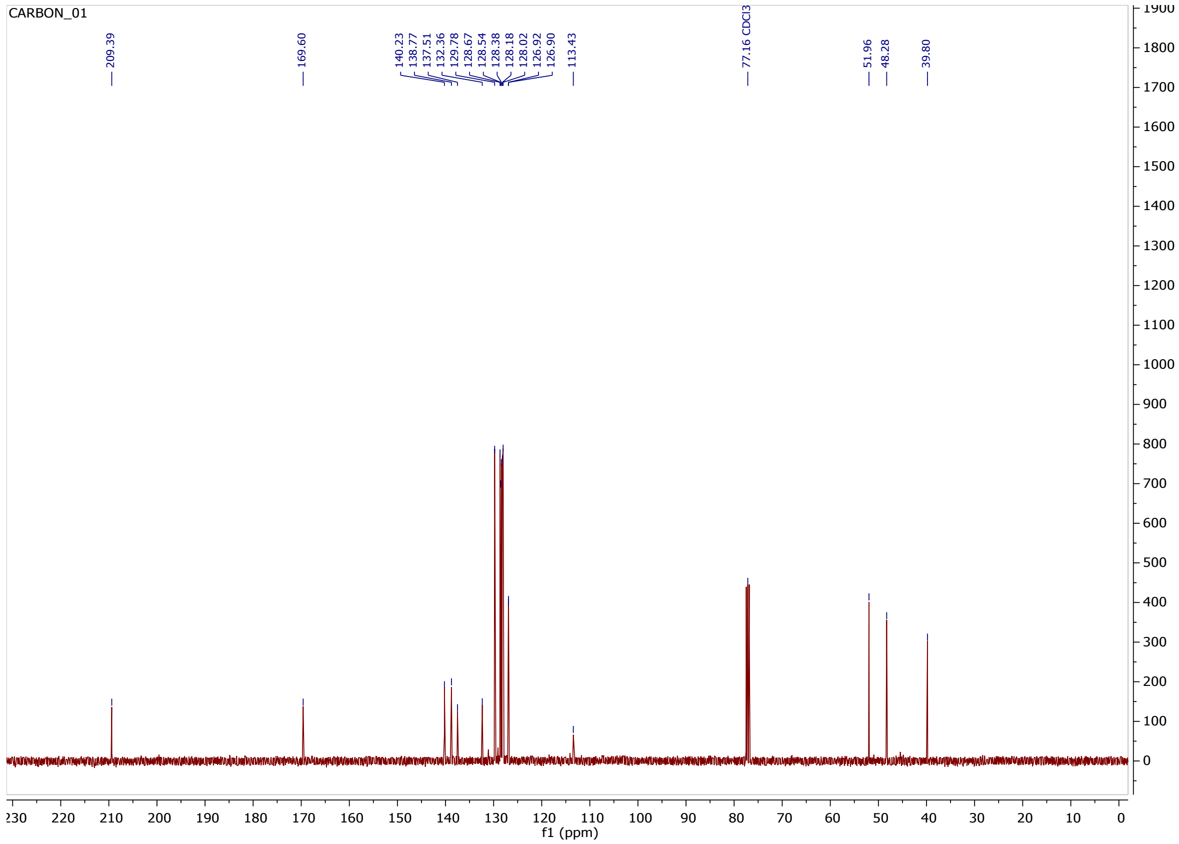

^{13}C {1H} NMR spectrum of **2o** ($CDCl_3$, 101 MHz).

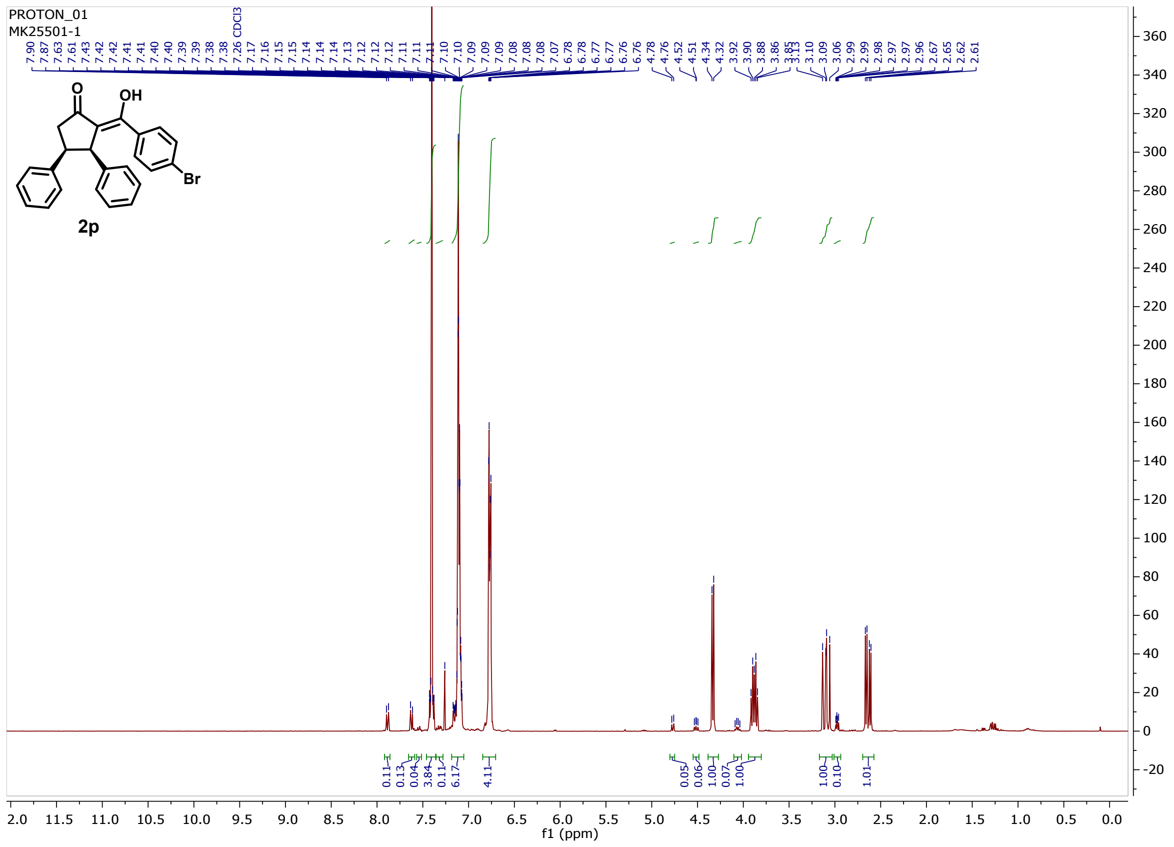

^{1}H NMR spectrum of **2p** ($CDCl_3$, 400 MHz).

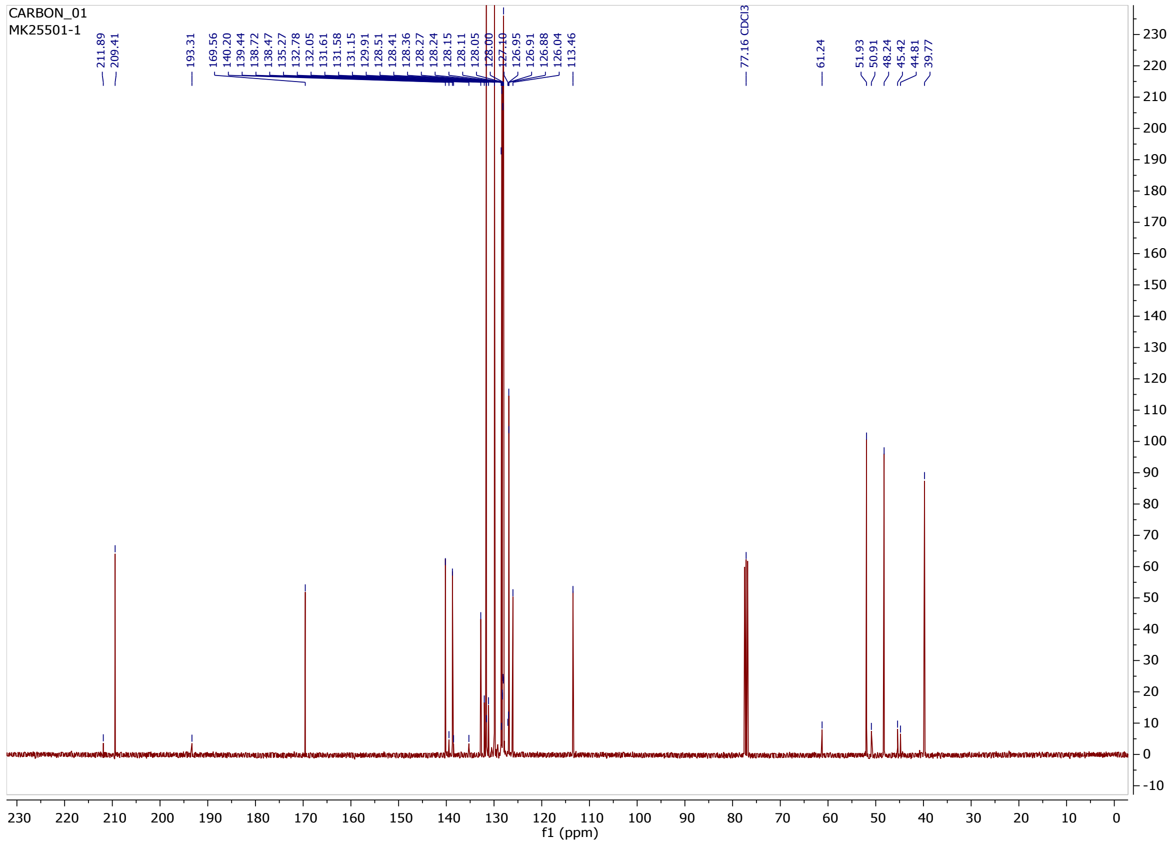

^{13}C {1H} NMR spectrum of **2p** ($CDCl_3$, 101 MHz).

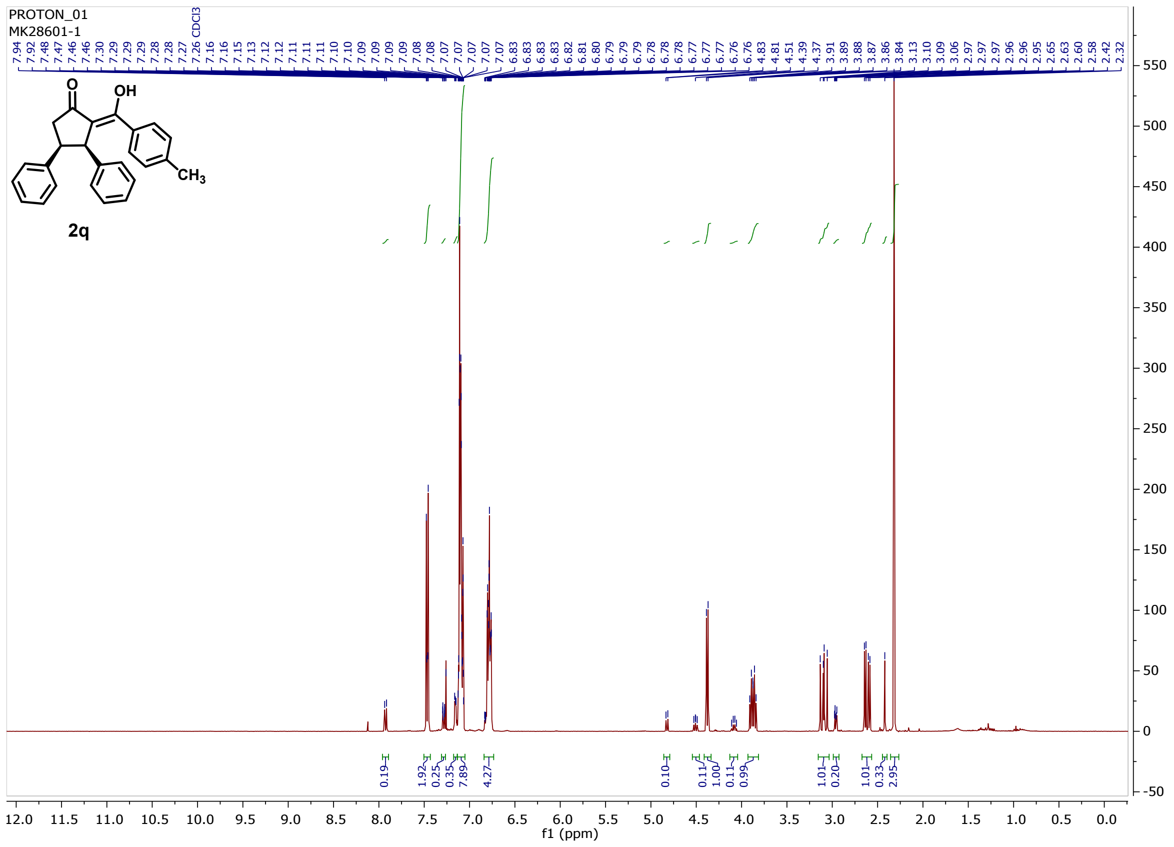

^{1}H NMR spectrum of **2q** ($CDCl_3$, 400 MHz).

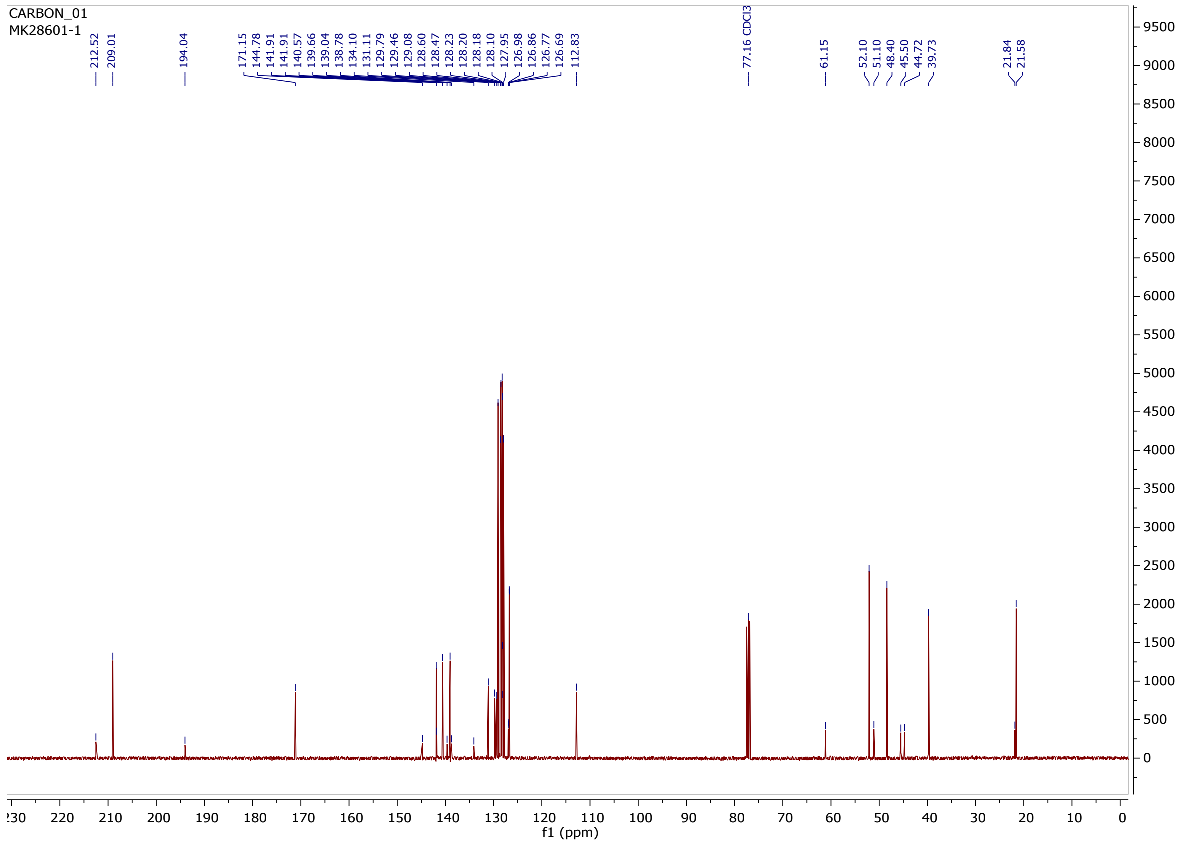

^{13}C {1H} NMR spectrum of **2q** ($CDCl_3$, 101 MHz).

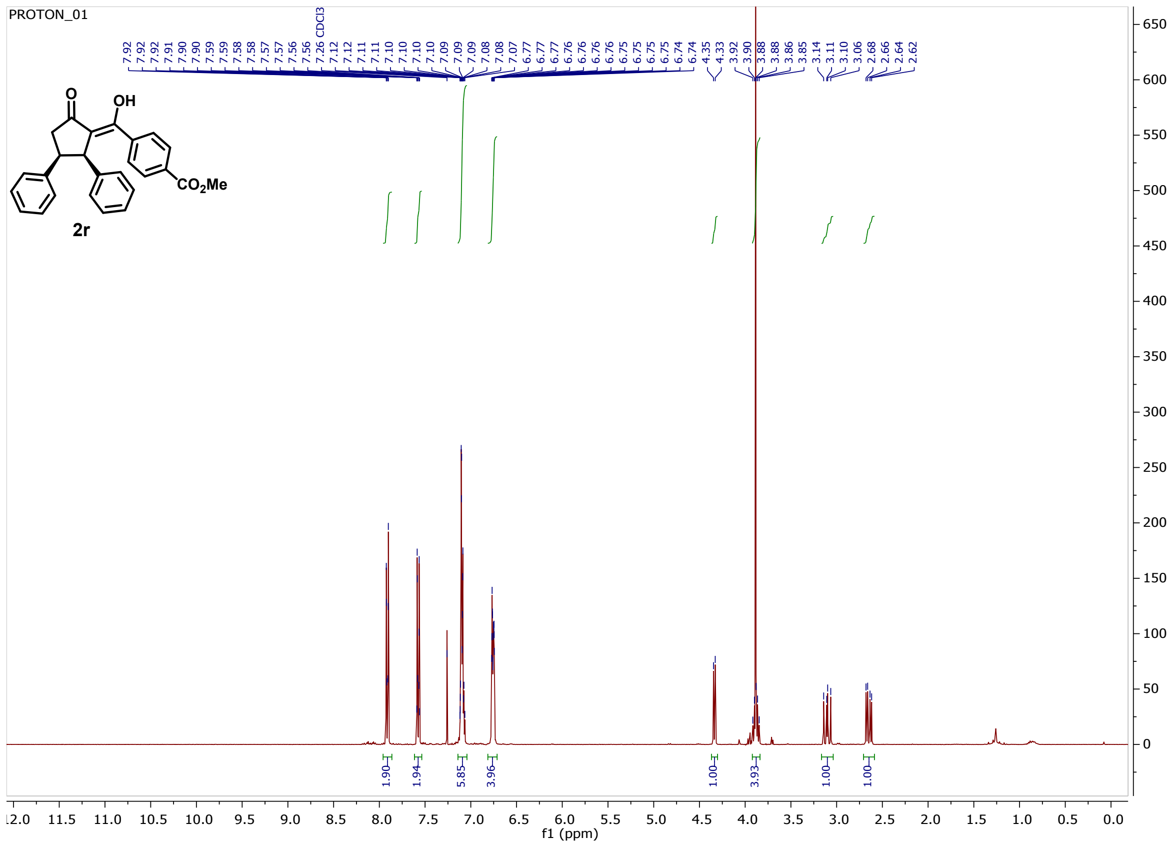

^{1}H NMR spectrum of **2r** ($CDCl_3$, 400 MHz).

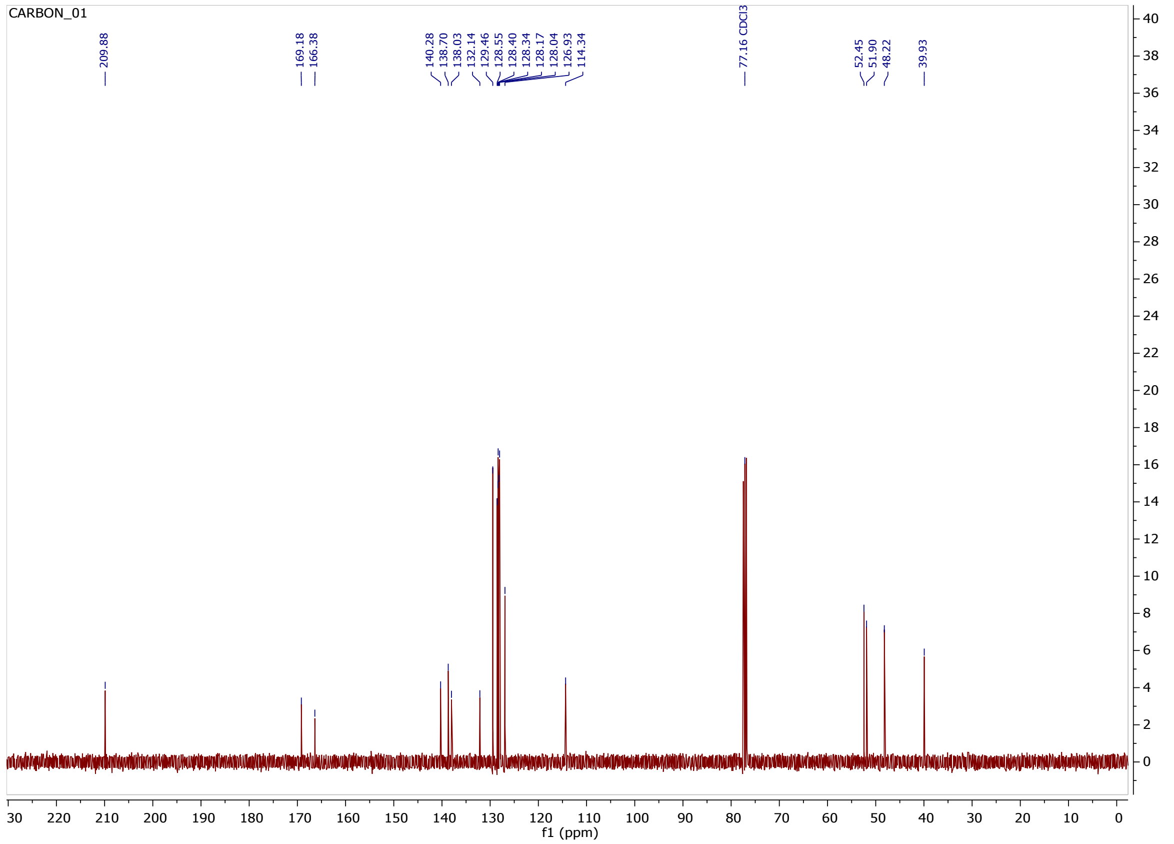

^{13}C {1H} NMR spectrum of **2r** ($CDCl_3$, 101 MHz).

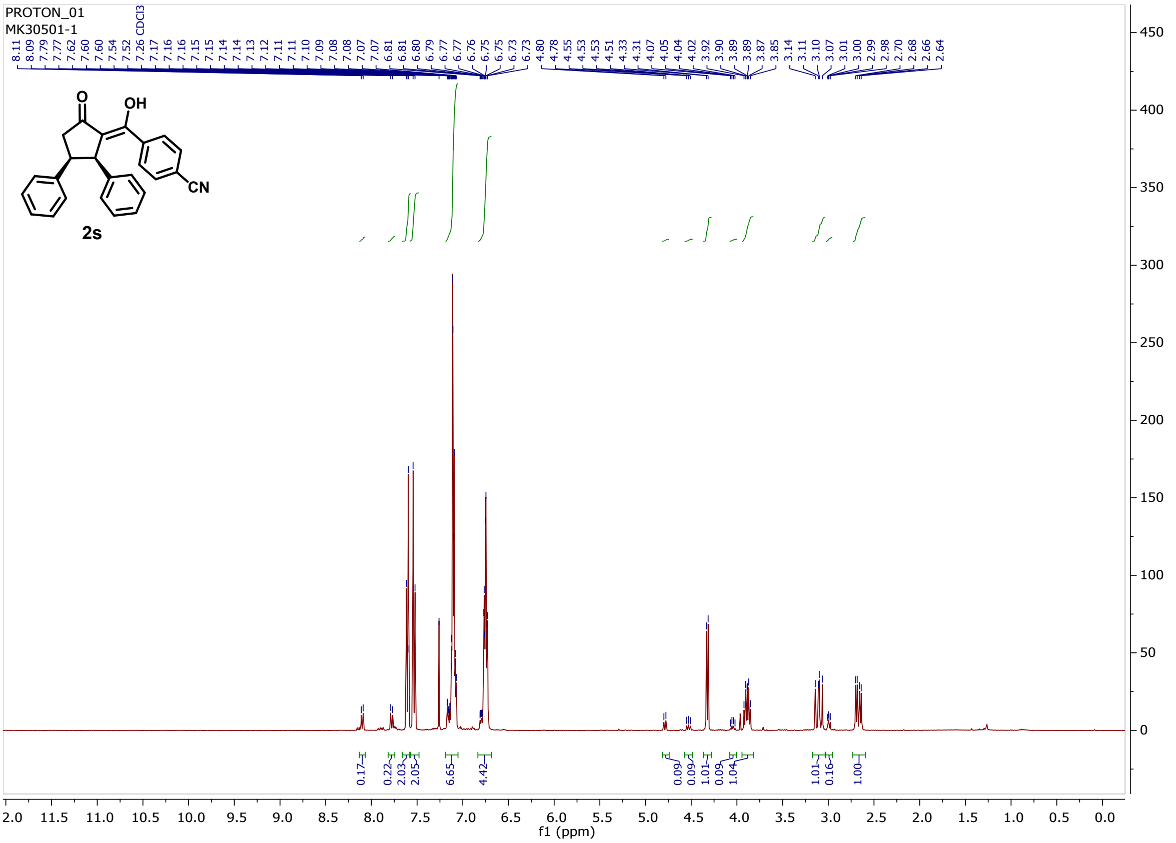

^{1}H NMR spectrum of **2s** ($CDCl_3$, 400 MHz).

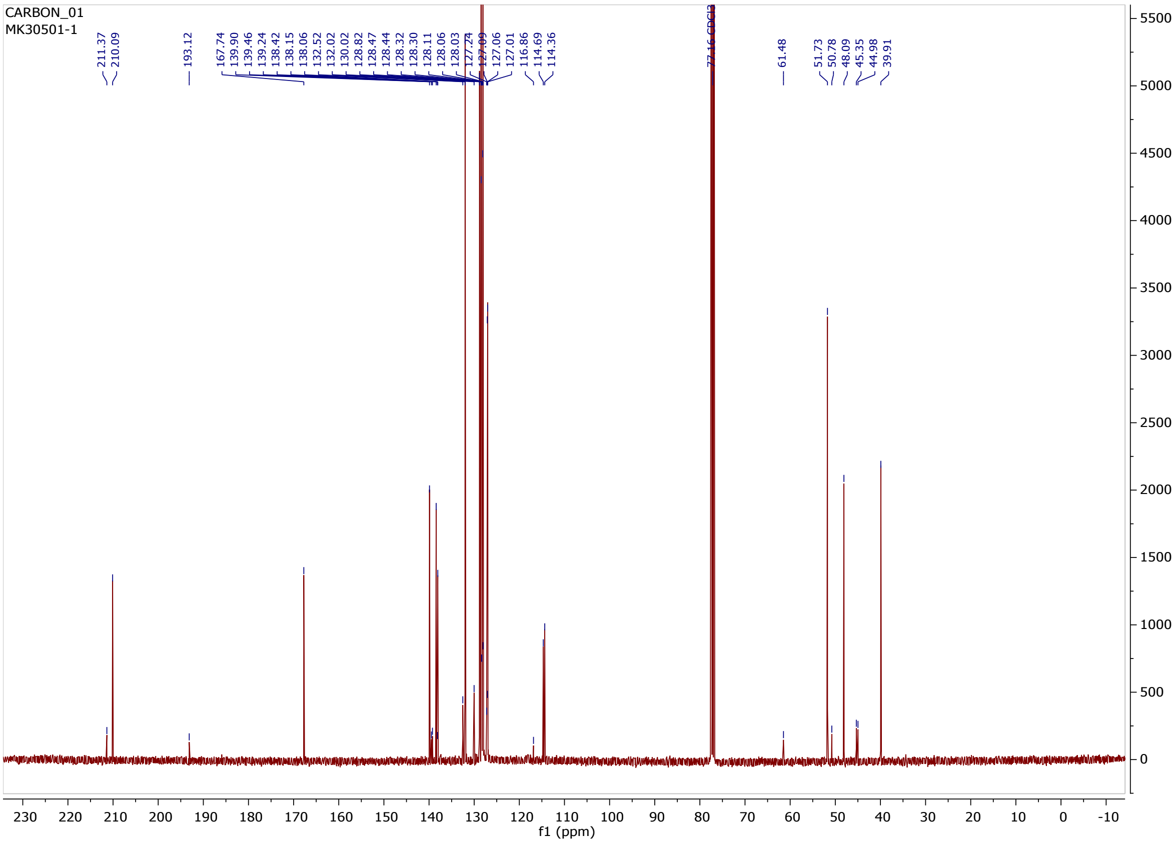

^{13}C {1H} NMR spectrum of **2s** ($CDCl_3$, 101 MHz).

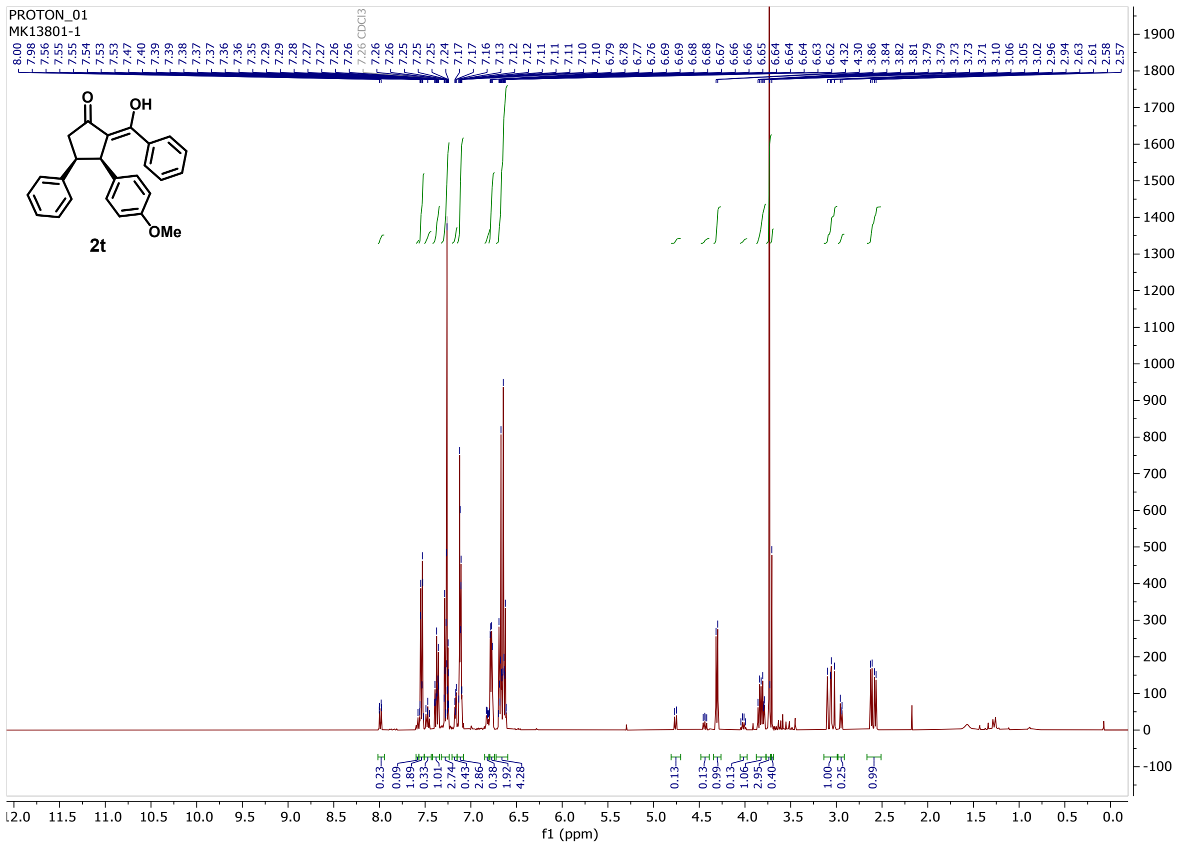

^{1}H NMR spectrum of **2t** ($CDCl_3$, 400 MHz).

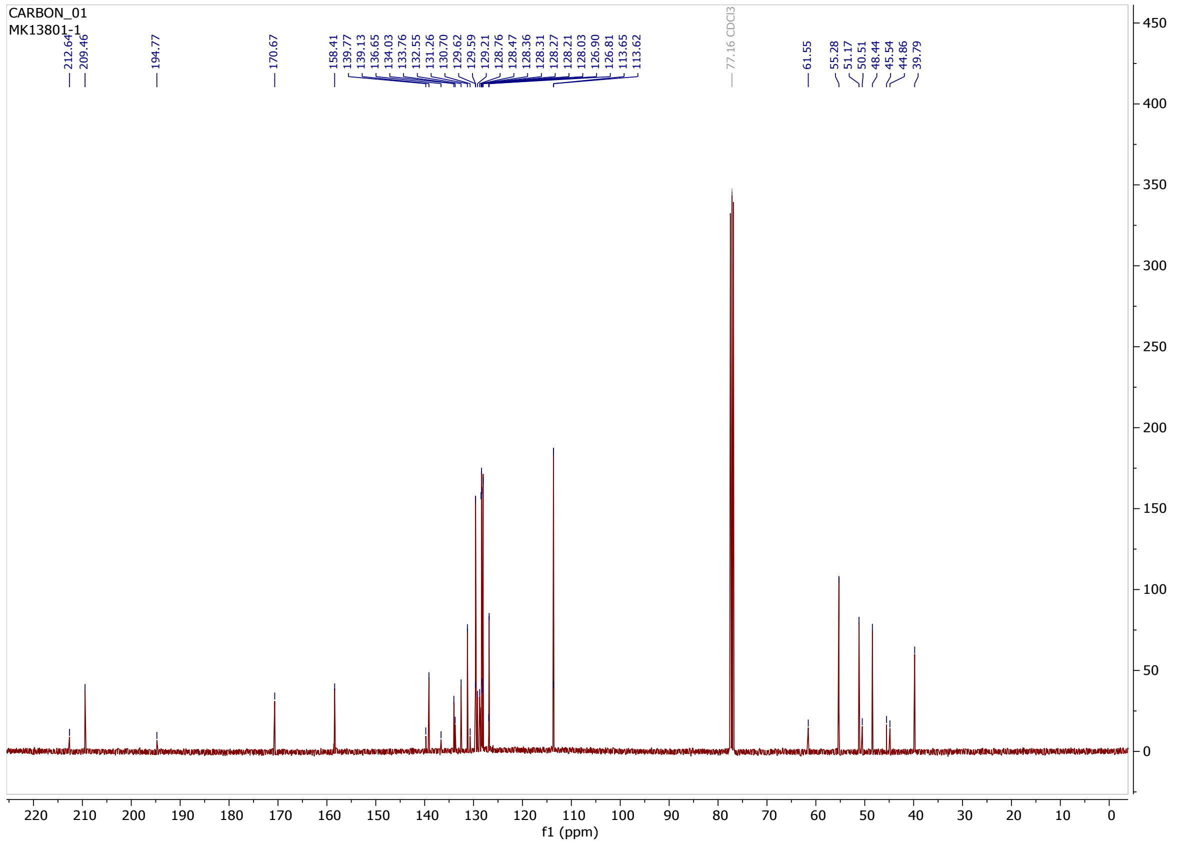

^{13}C {1H} NMR spectrum of **2t** ($CDCl_3$, 101 MHz).

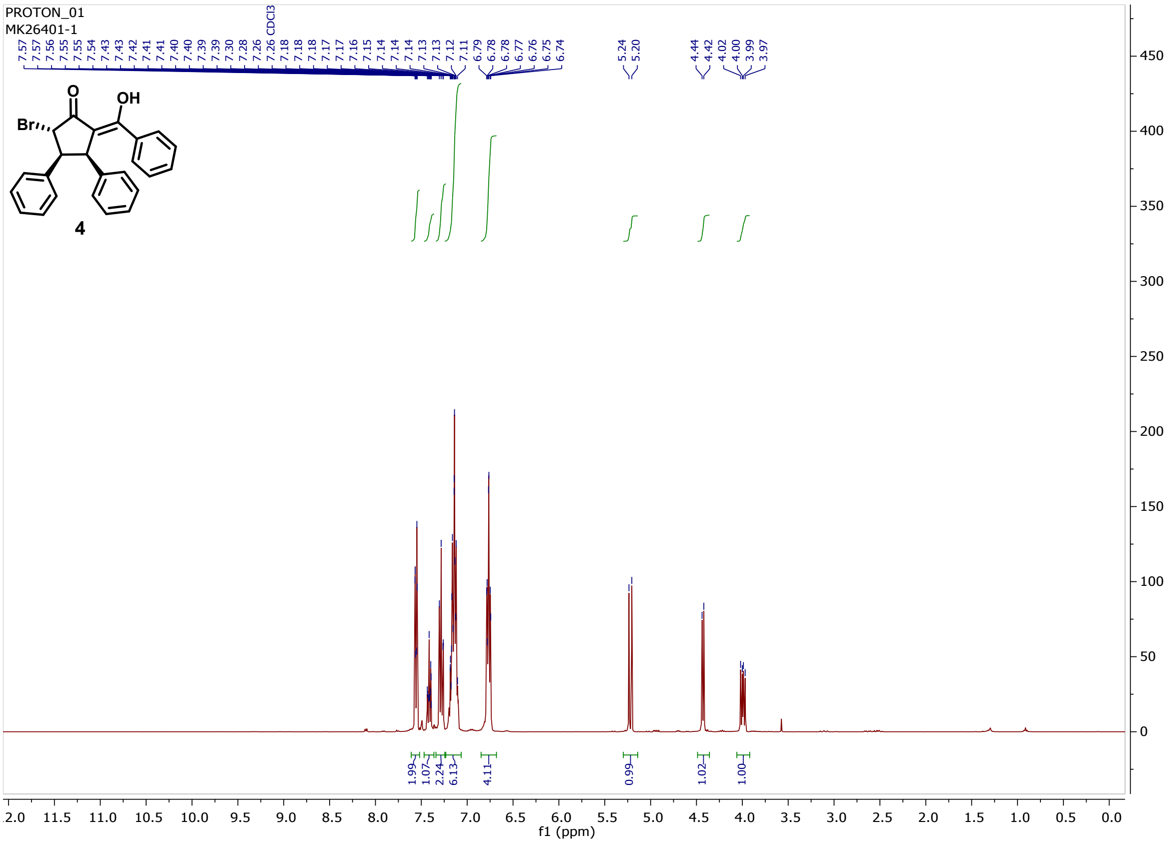

^{1}H NMR spectrum of **4** ($CDCl_3$, 400 MHz).

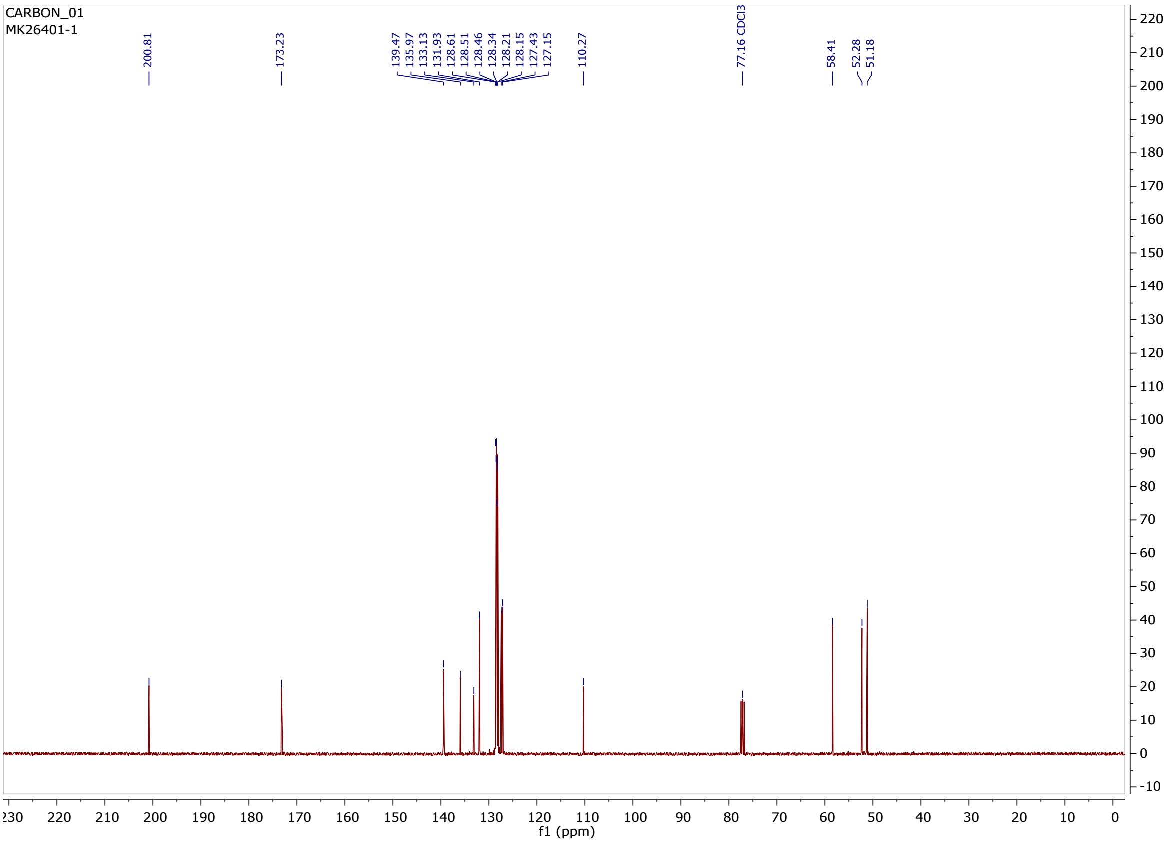

^{13}C {1H} NMR spectrum of **4** ($CDCl_3$, 101 MHz).

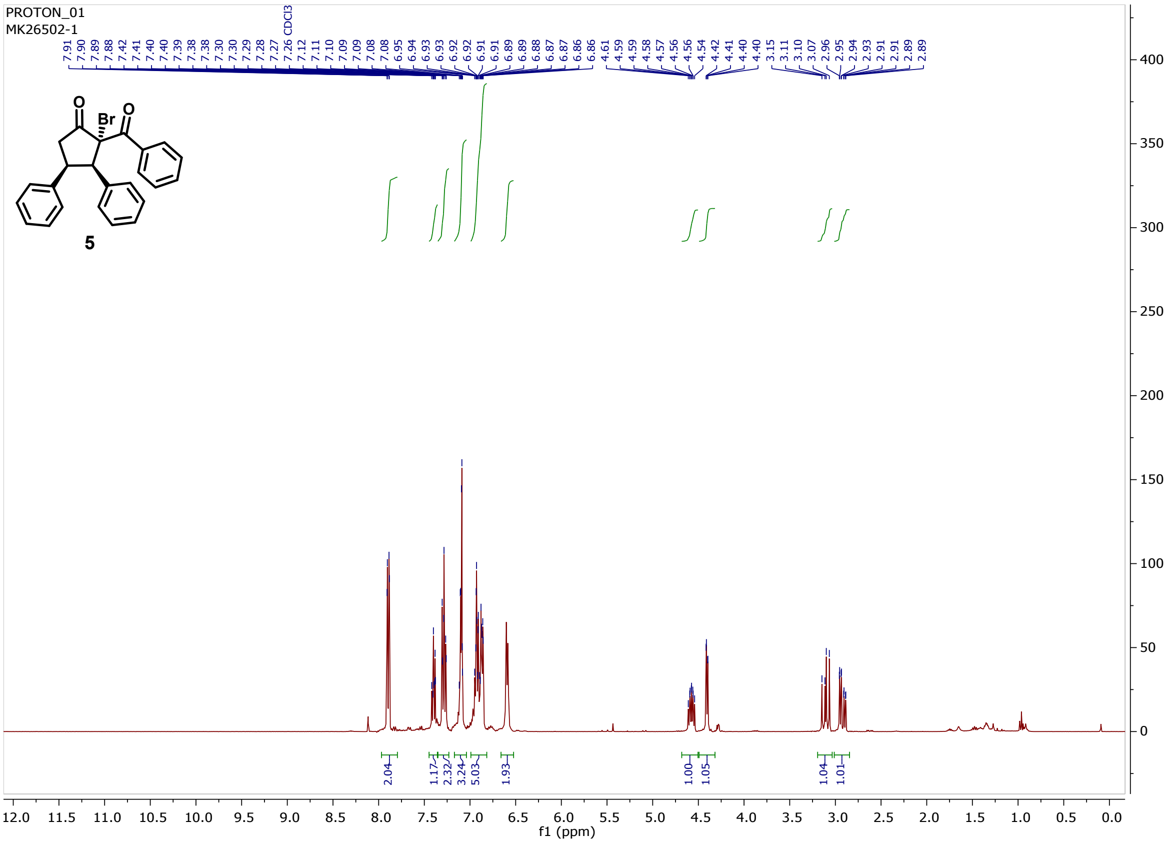

^{1}H NMR spectrum of **5** ($CDCl_3$, 400 MHz).

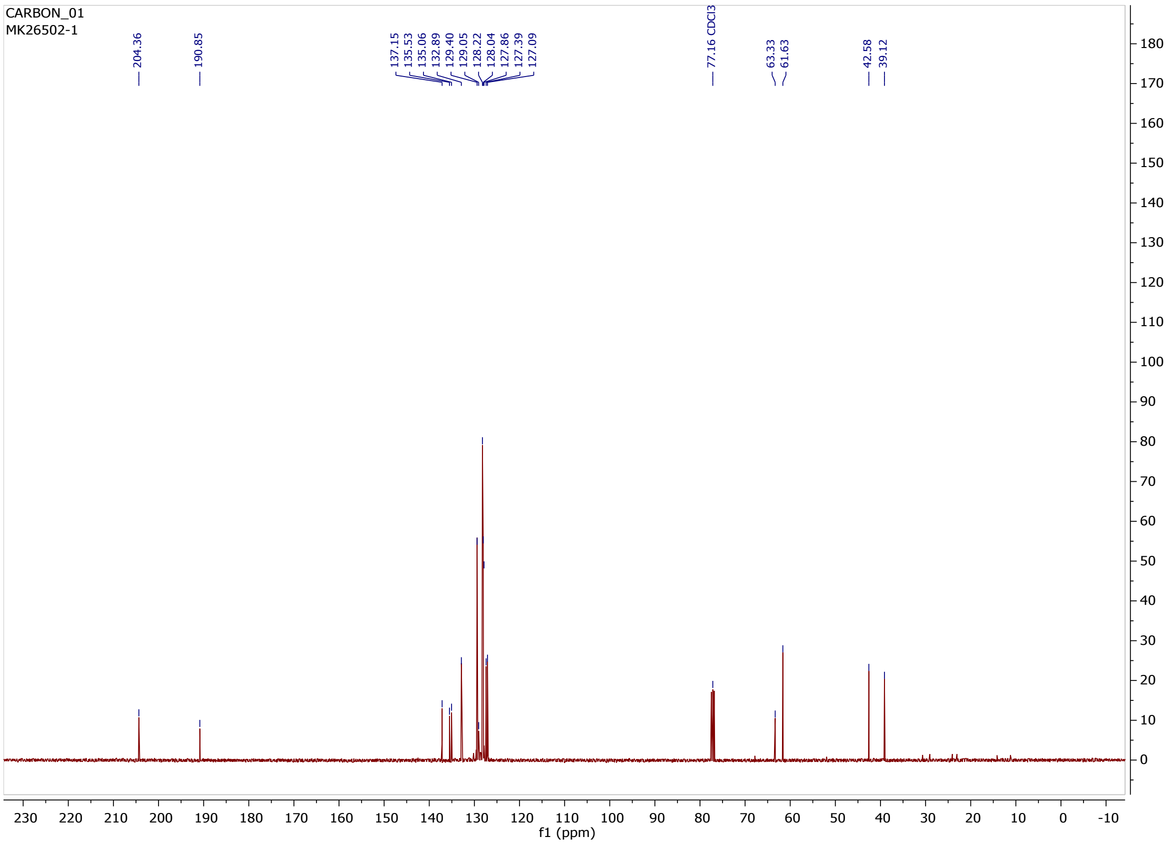

^{13}C {1H} NMR spectrum of **5** ($CDCl_3$, 101 MHz).

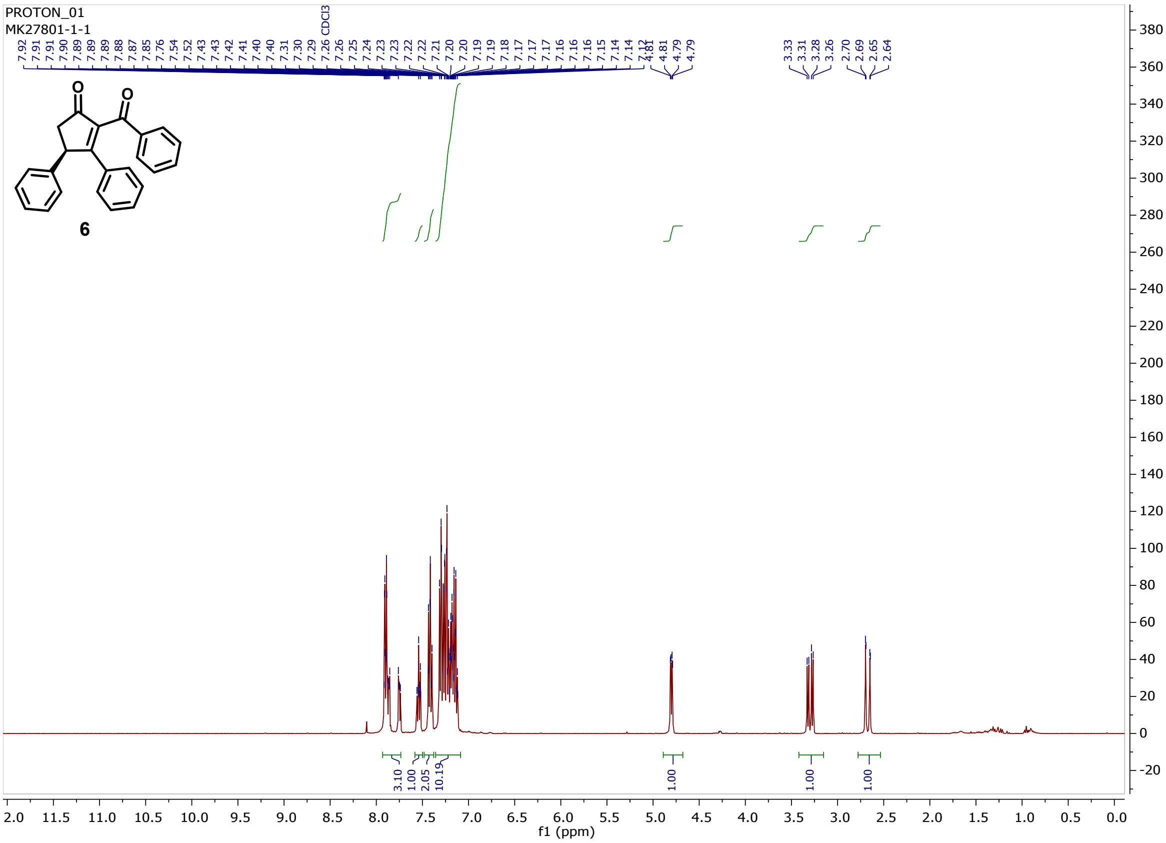

^{1}H NMR spectrum of **6** ($CDCl_3$, 400 MHz).

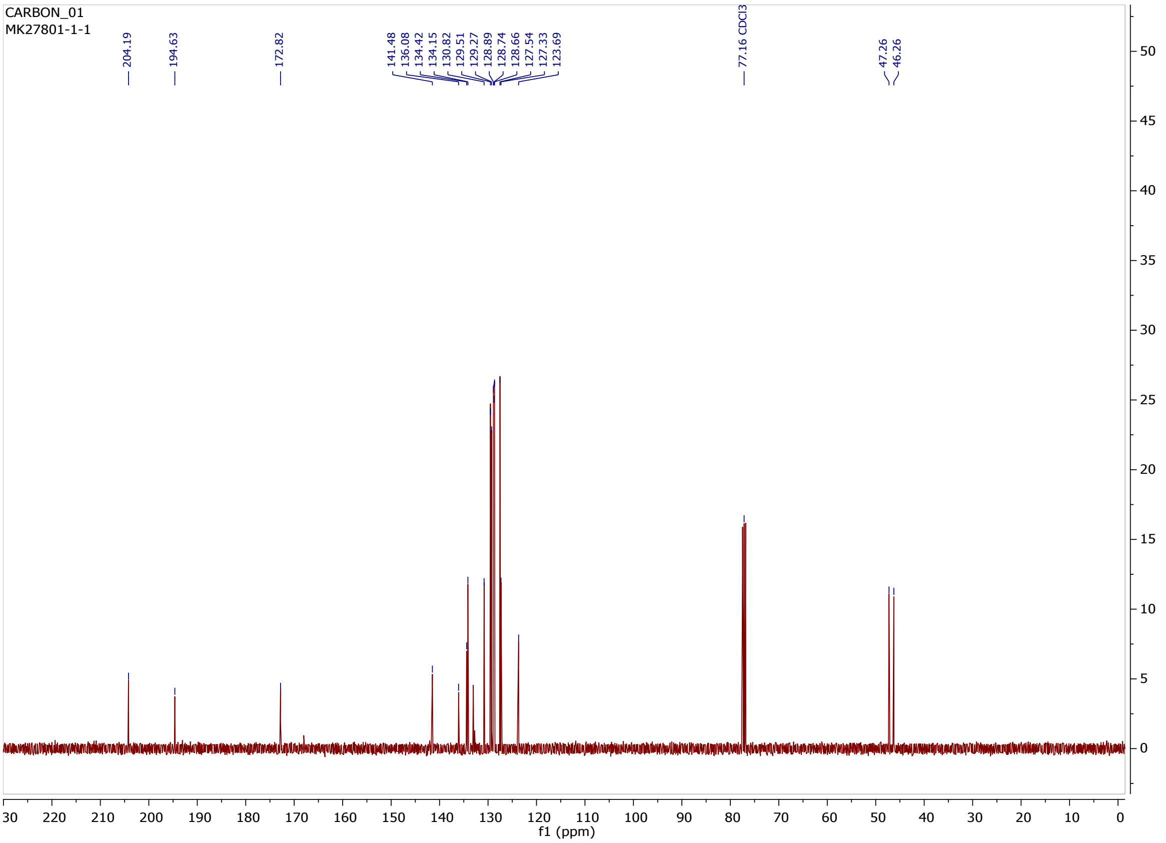

^{13}C {1H} NMR spectrum of **6** ($CDCl_3$, 101 MHz).

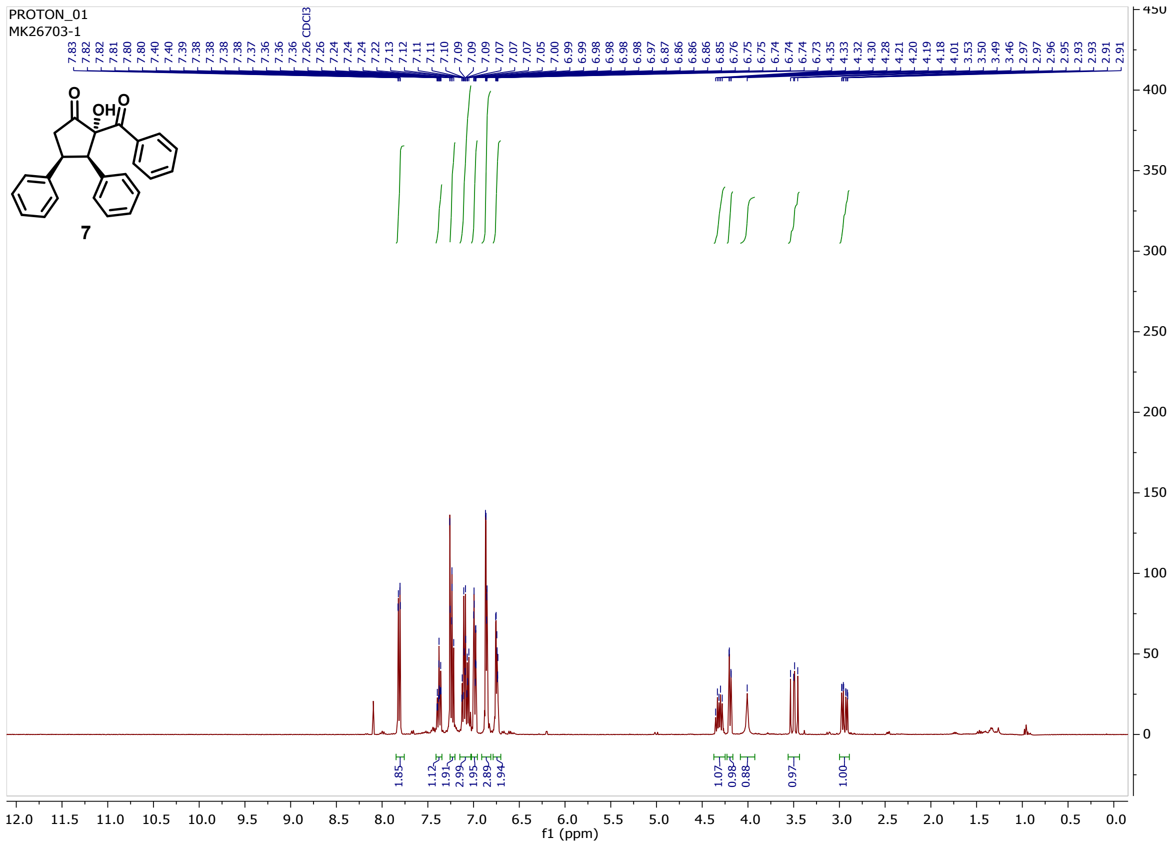

^{1}H NMR spectrum of **7** ($CDCl_3$, 400 MHz).

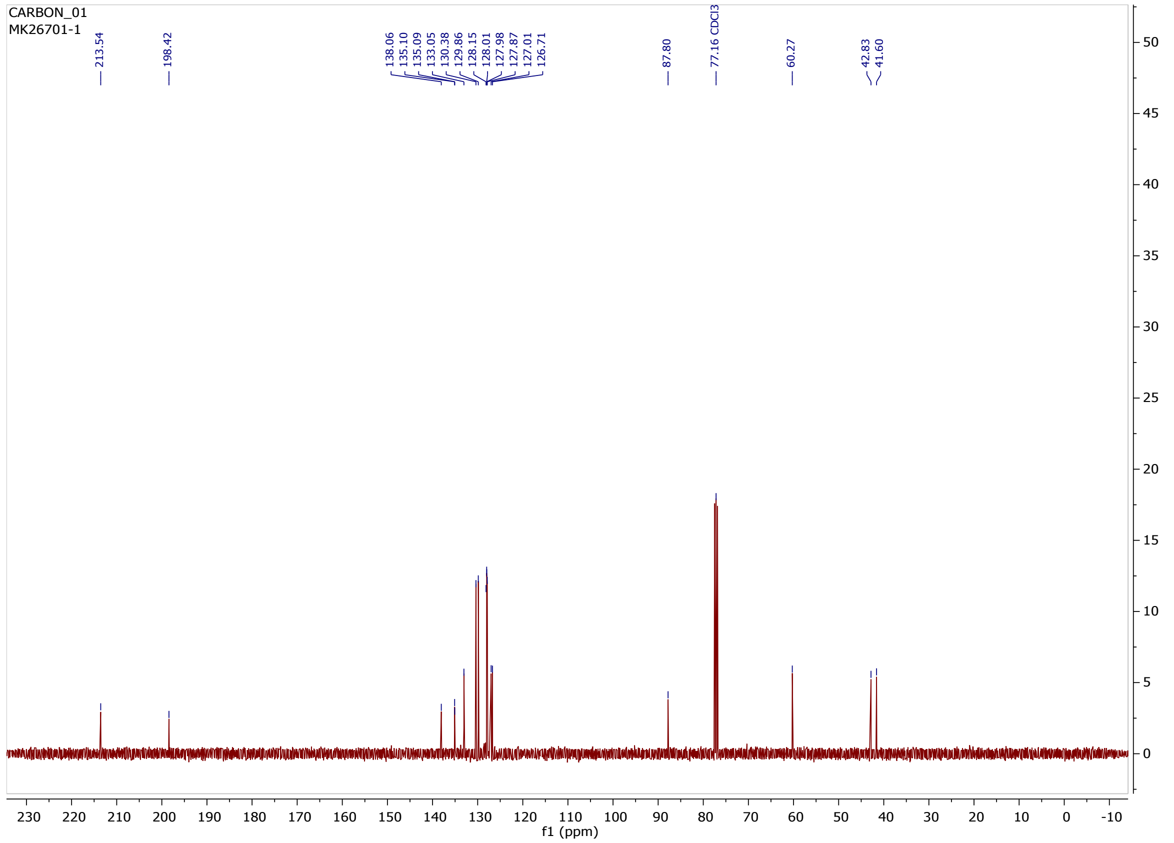

^{13}C {1H} NMR spectrum of **7** ($CDCl_3$, 101 MHz).

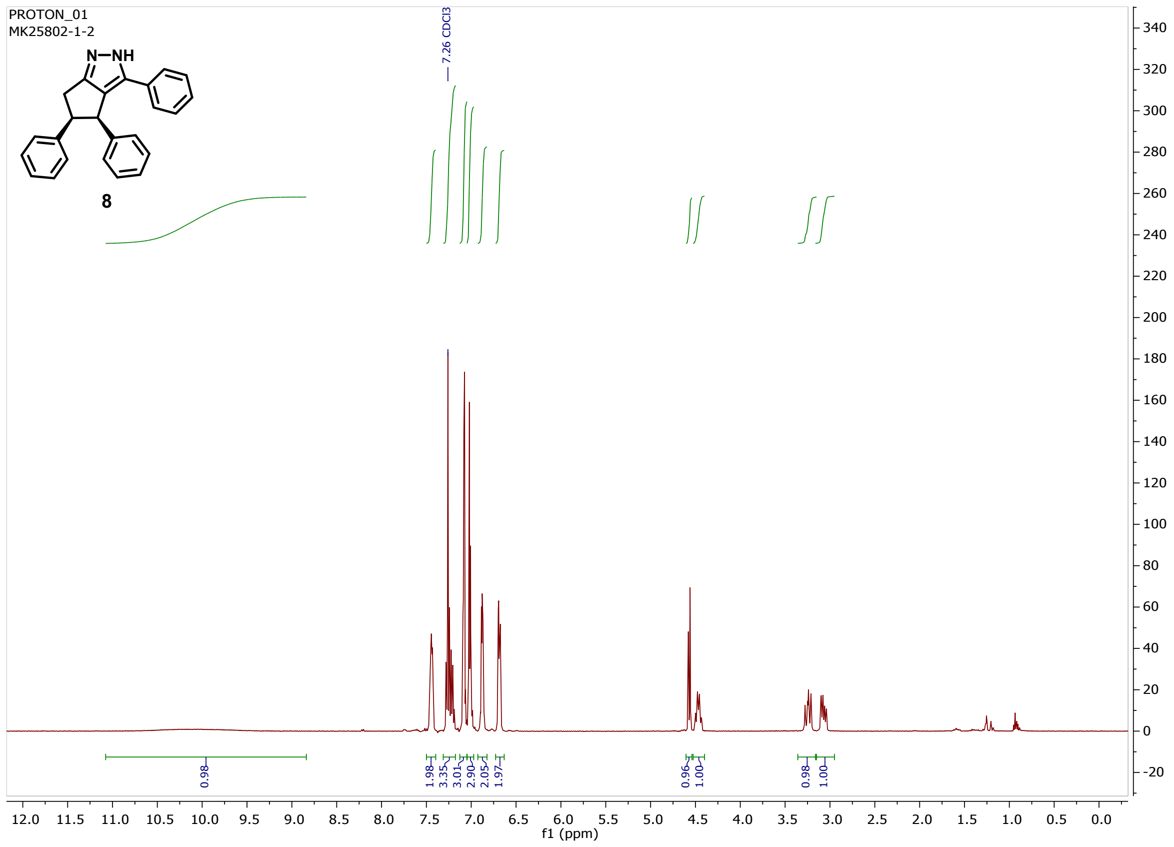

^{1}H NMR spectrum of **8** ($CDCl_3$, 400 MHz).

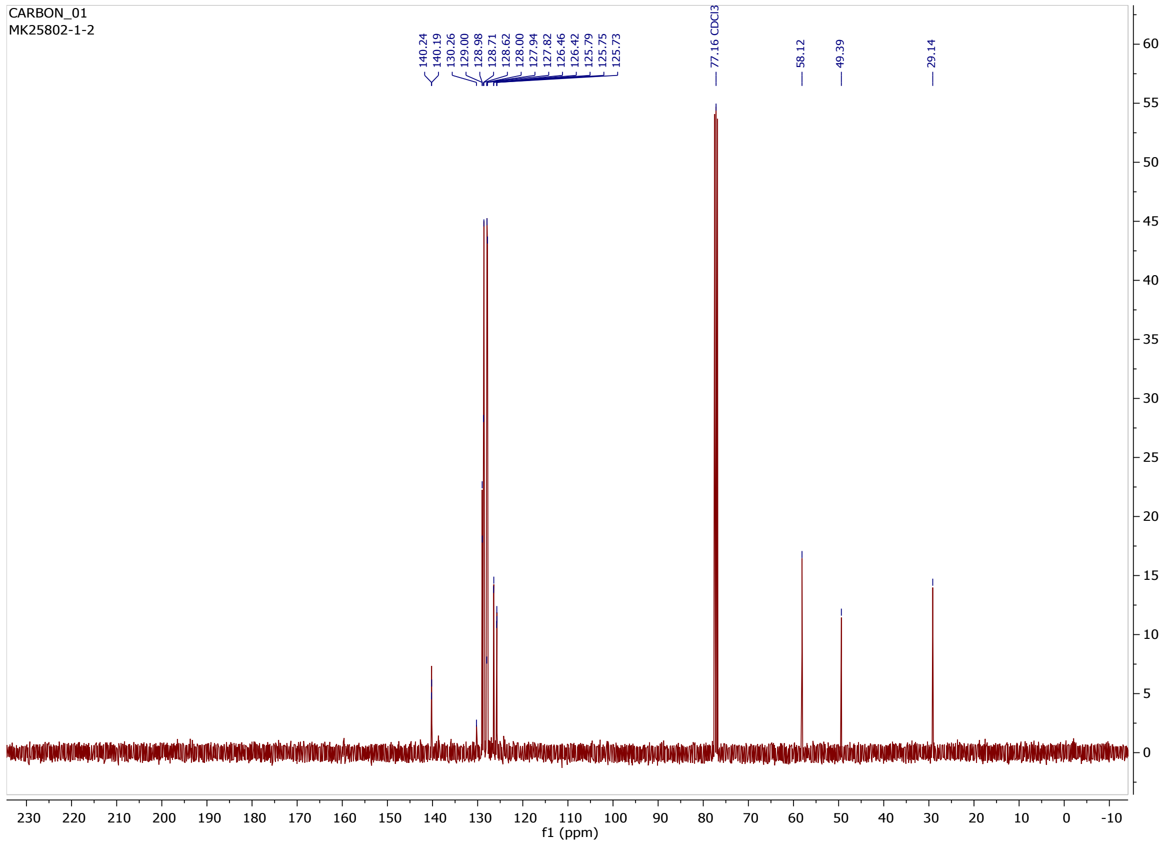

^{13}C {1H} NMR spectrum of **8** ($CDCl_3$, 101 MHz).

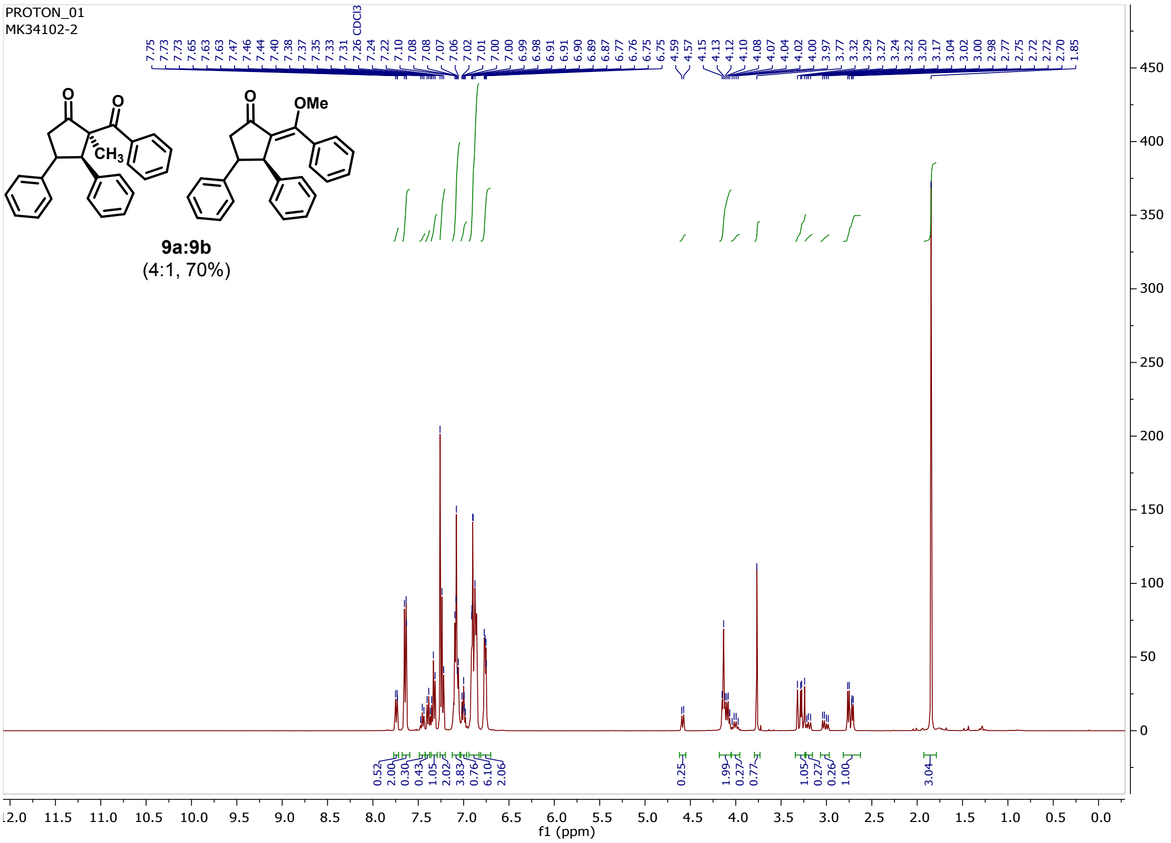

^{1}H NMR spectrum of **9a** and **9b** ($CDCl_3$, 400 MHz).

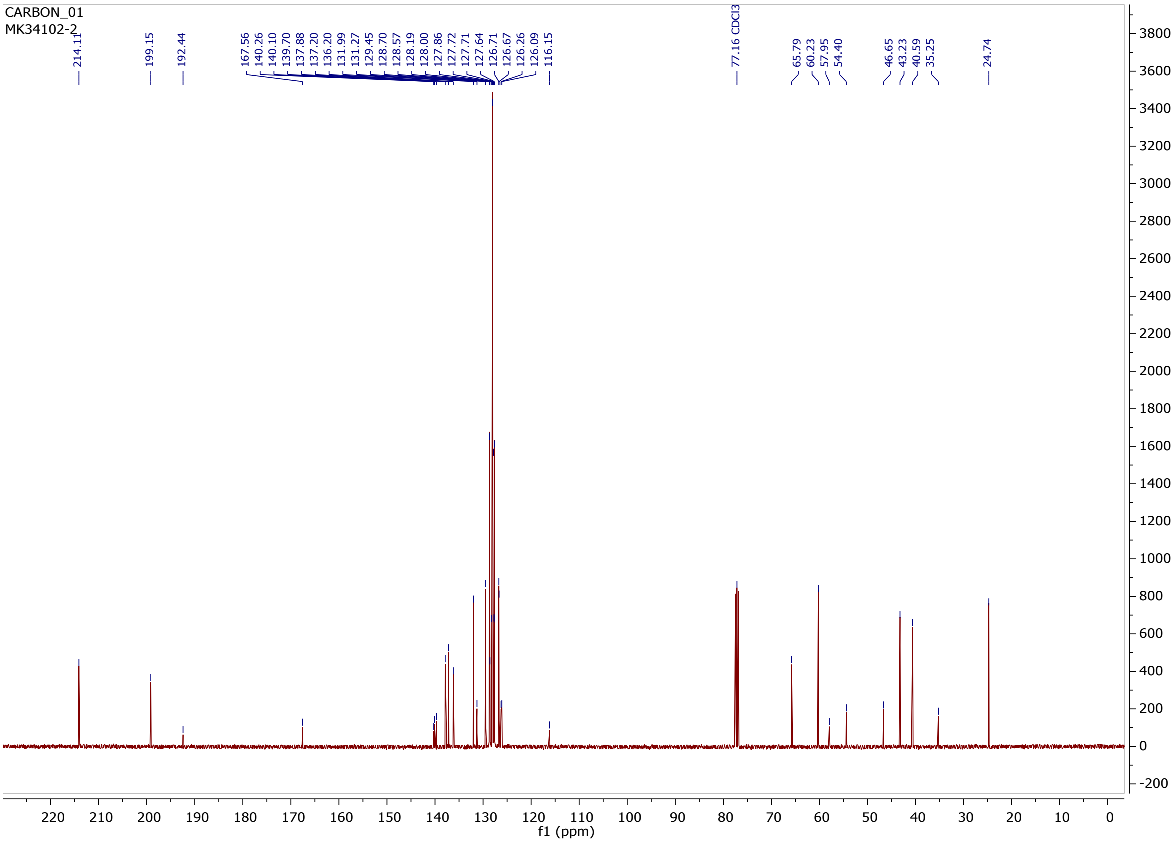

^{13}C {1H} NMR spectrum of **9a** and **9b** ($CDCl_3$, 101 MHz).

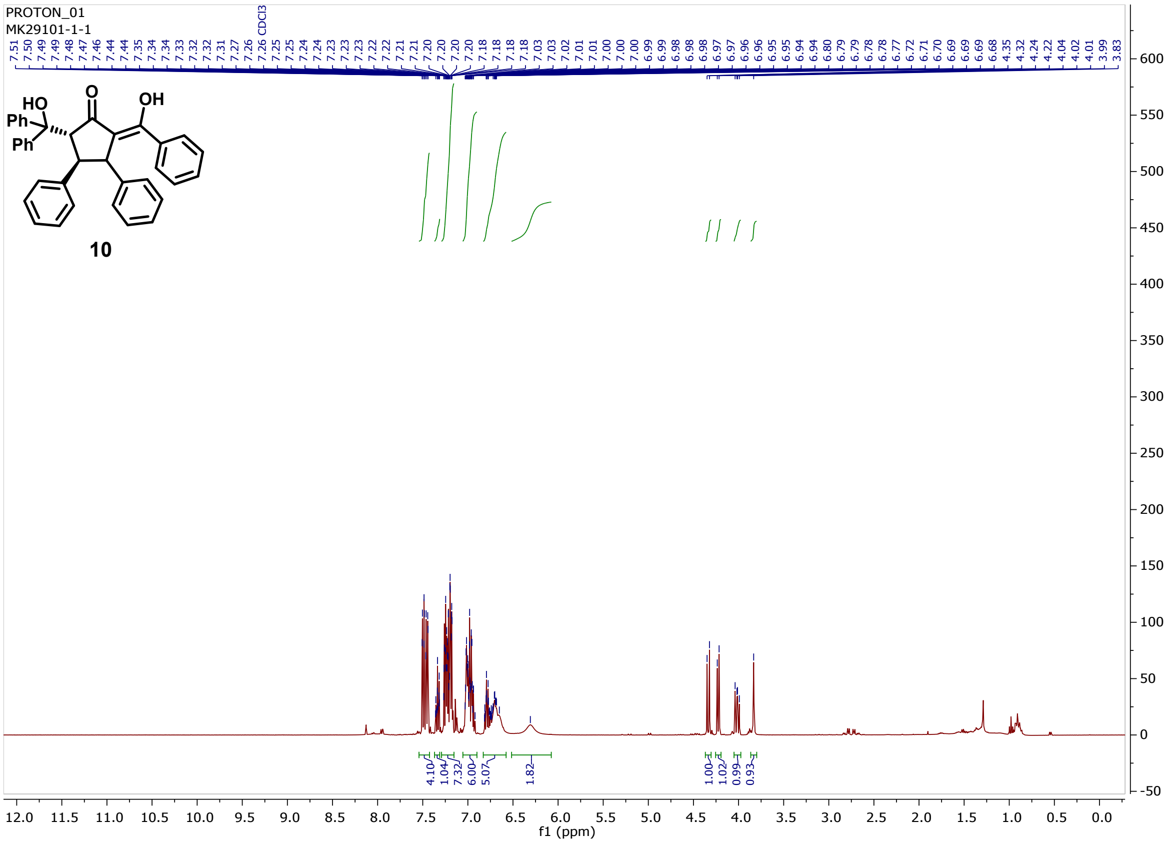

^{1}H NMR spectrum of **10** ($CDCl_3$, 400 MHz).

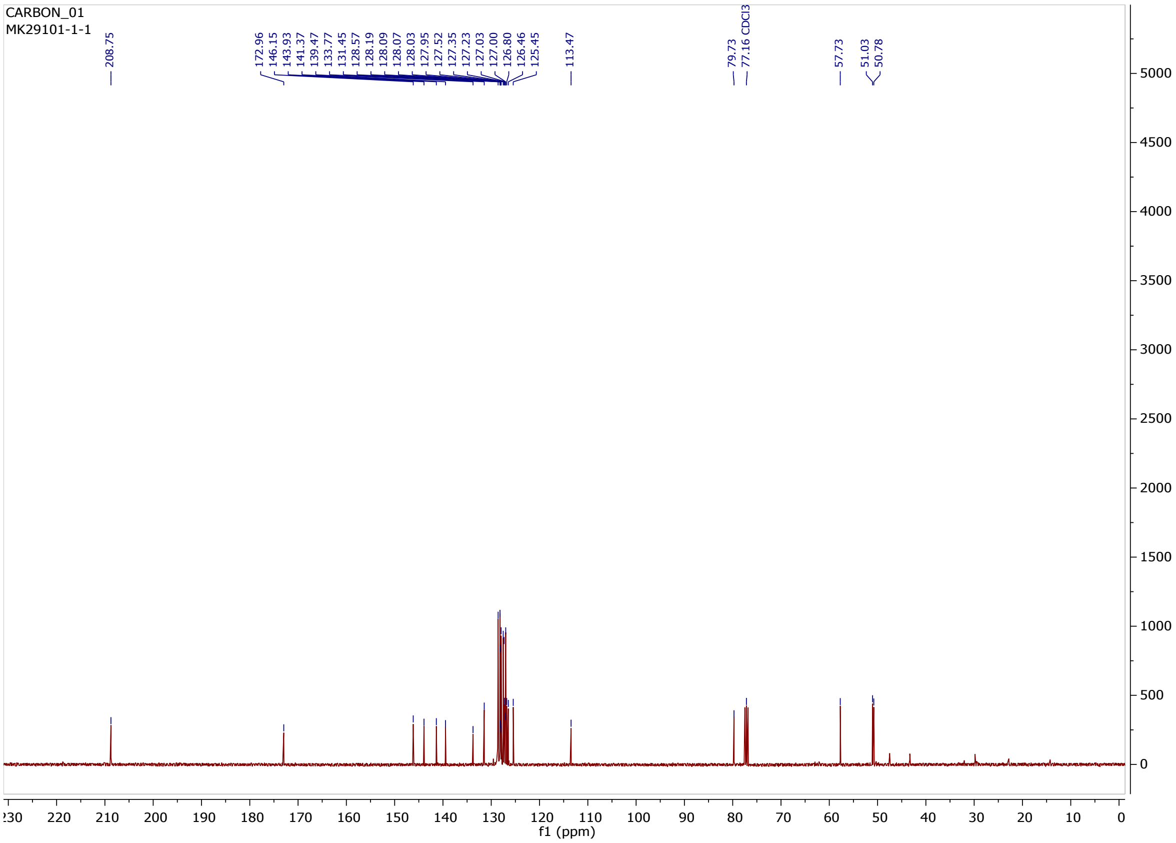

^{13}C {1H} NMR spectrum of **10** ($CDCl_3$, 101 MHz).

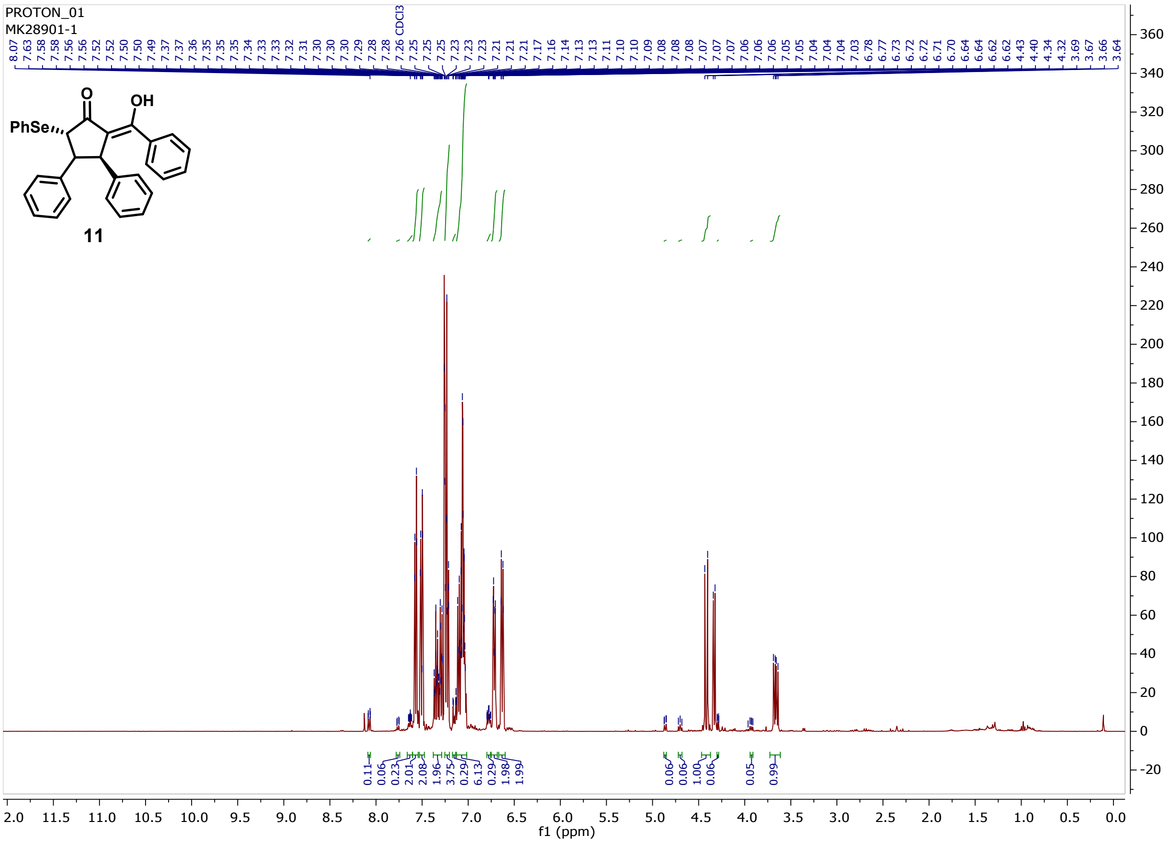

^{1}H NMR spectrum of **11** ($CDCl_3$, 400 MHz).

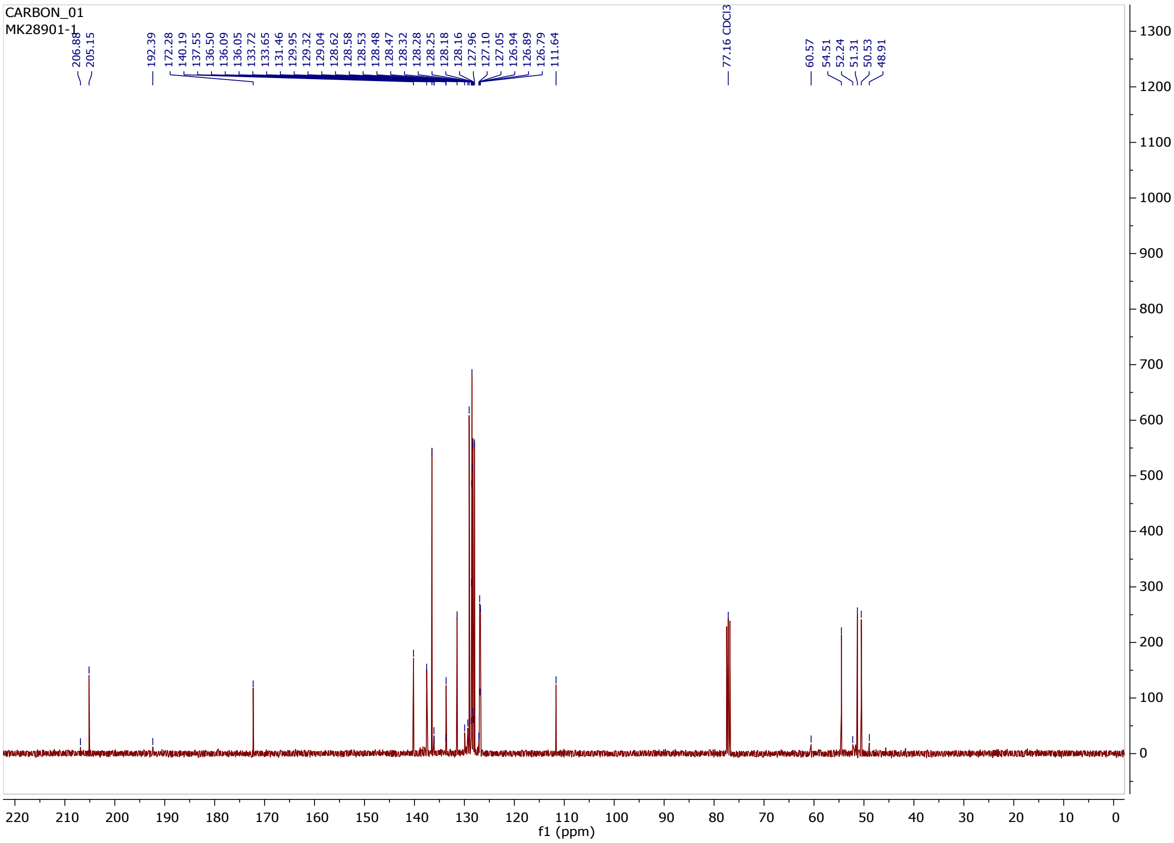

^{13}C {1H} NMR spectrum of **11** ($CDCl_3$, 101 MHz).

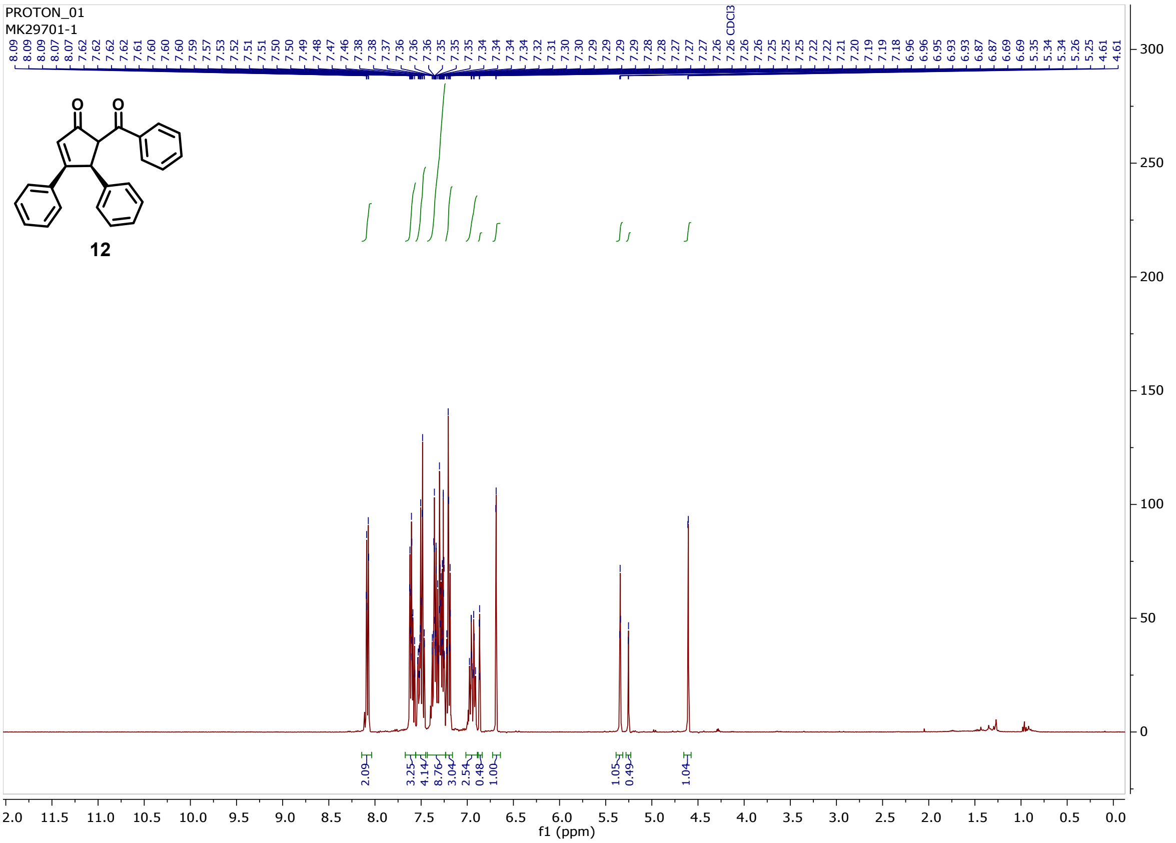

^{1}H NMR spectrum of **12** ($CDCl_3$, 400 MHz).

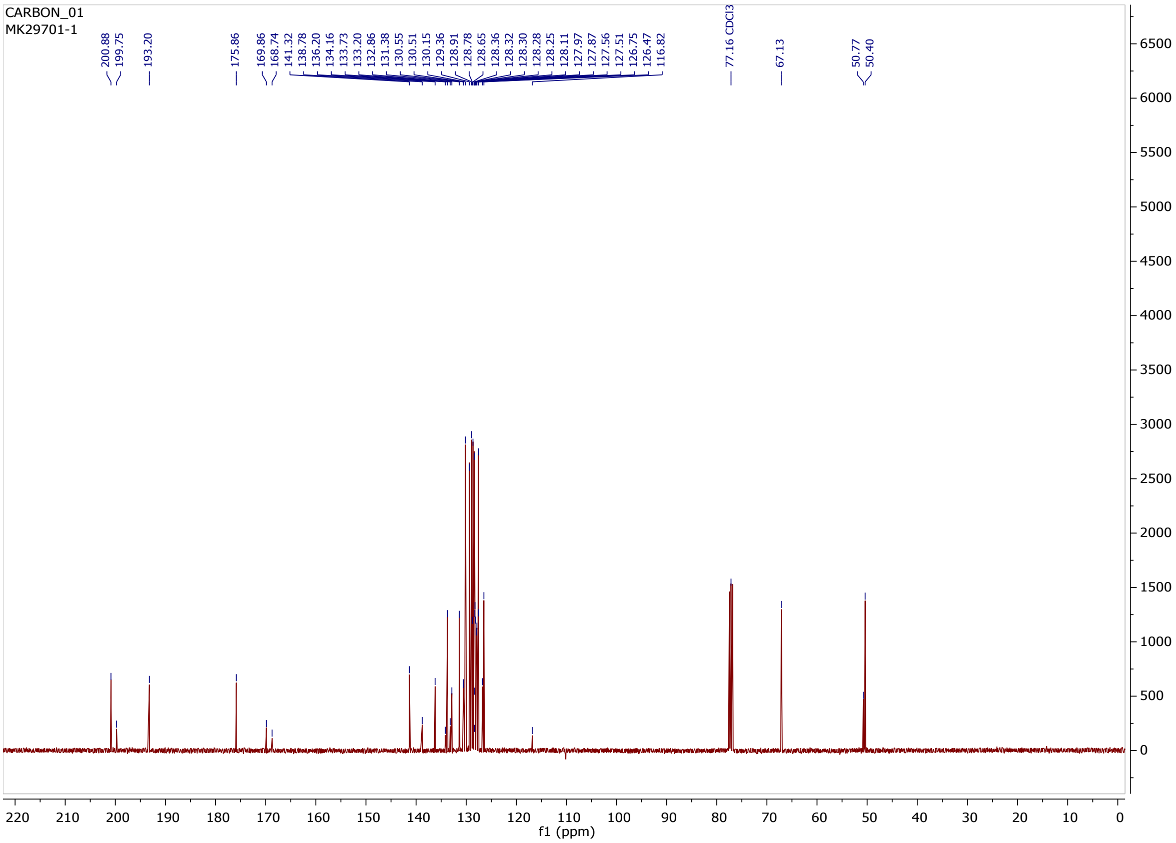

^{13}C {1H} NMR spectrum of **12** ($CDCl_3$, 101 MHz).

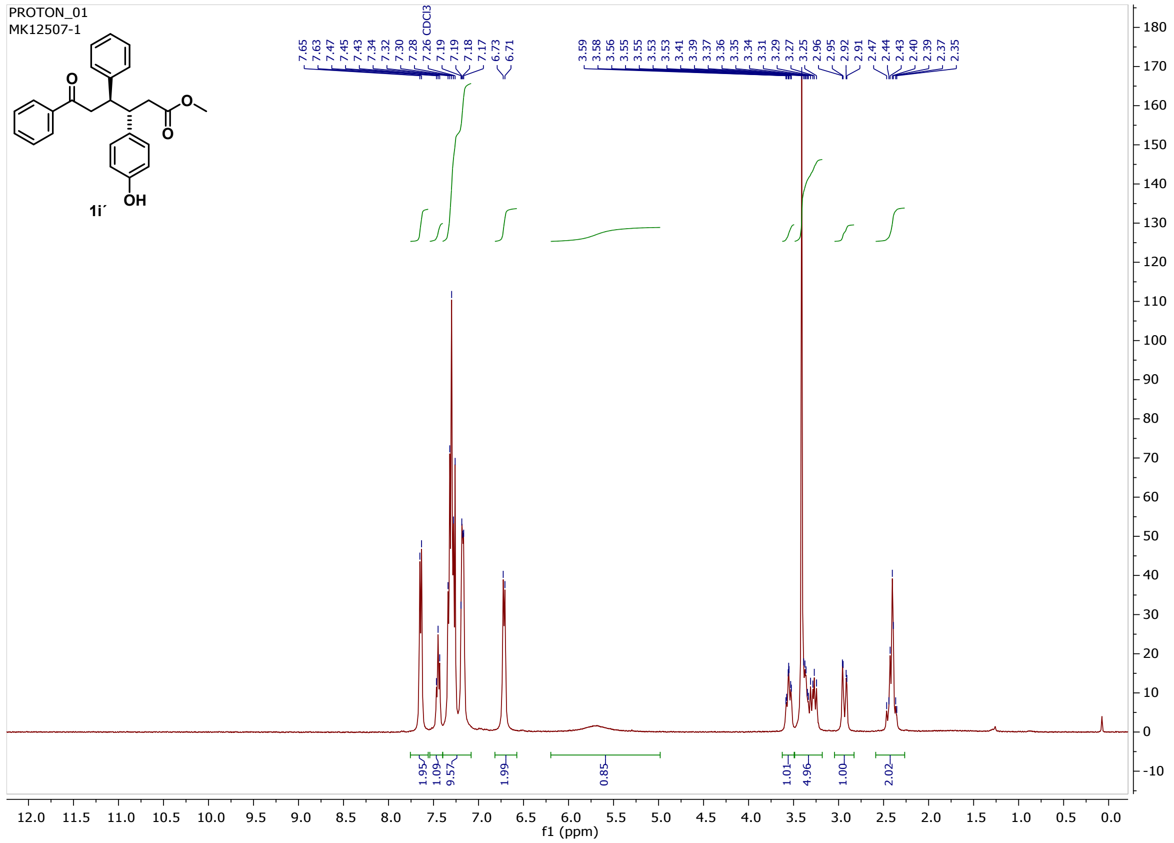

^{1}H NMR spectrum of **1i´** ($CDCl_3$, 400 MHz).

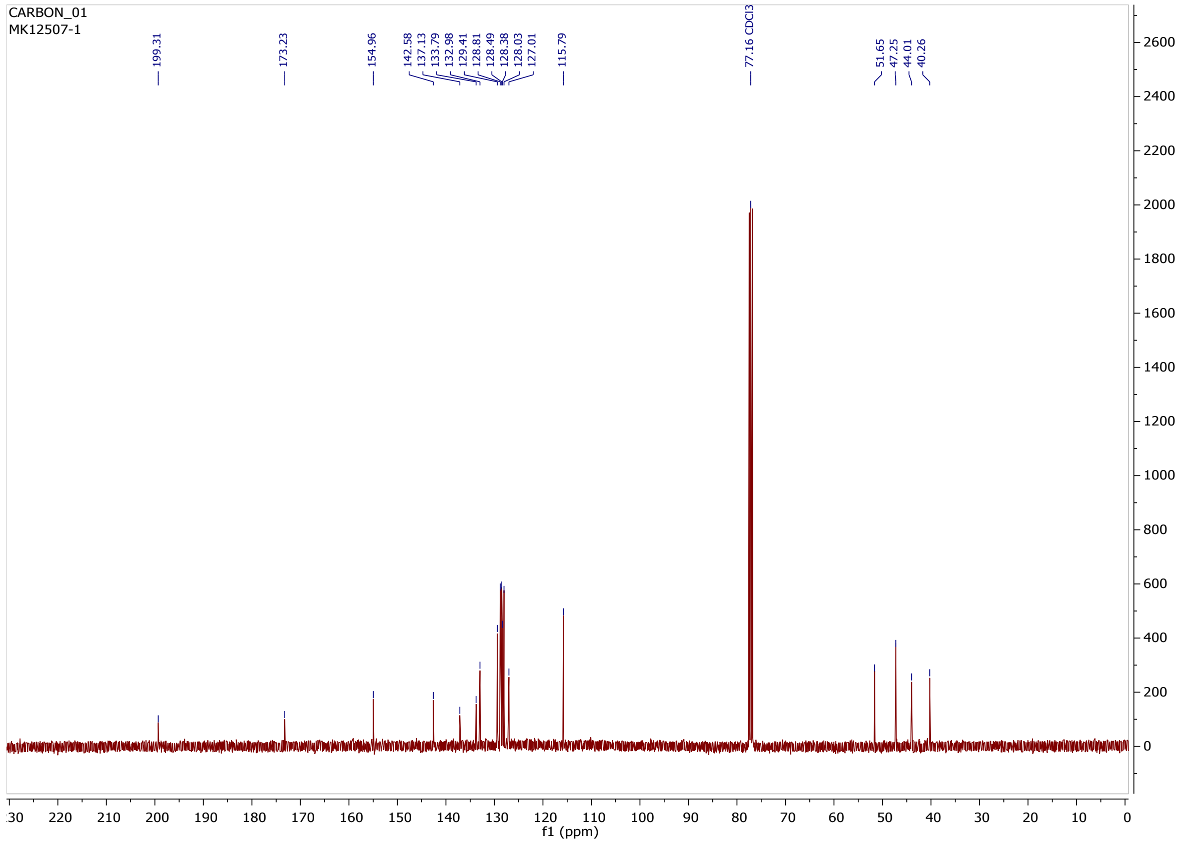

^{13}C {1H} NMR spectrum of **1i´** ($CDCl_3$, 101 MHz).

X-ray data collection

Crystallographic data for **2a, 4** and **5** were collected on Bruker D8 VENTURE Kappa Duo PHOTONIII by IμS micro-focus sealed tube either MoKα (λ= 0.71073) (**5)** or CuKα (λ = 1.54178 Å) (**2a**, **4**) at low temperature 120K. The structures were solved by direct methods (XT[1]) and refined by full matrix least squares based on F^2 (SHELXL2018[2]). The hydrogen atoms on carbon were fixed into idealized positions (riding model) and assigned temperature factors either $H_{iso}(H) = 1.2\ U_{eq}$(pivot atom) or $H_{iso}(H) = 1.5\ U_{eq}$ (pivot atom) for methyl moiety. The hydrogen atoms in -O-H moieties were found on difference Fourier map and refined with the assumptions of riding model. The measured crystals of **2a** was refined as non-merohedral twin with the twin matrix applied on *hkl* indicies: -1 0 0; 0 -1 0; 0.117 0 1, with domains ration 0.66:0.34. The low precision of these results excludes reliable determination of absolute structure of **2a**.

X-ray crystallographic data have been deposited with the Cambridge Crystallographic Data Centre under deposition number CCDC **2063174**, **2063176** and **2063175** for **2a, 4** and **5**, respectively and can be obtained free of charge from the Centre via its website (www.ccdc.cam.ac.uk/getstructures).

1. SHELXT: Sheldrick, G.M. (2015). *Acta Cryst.* **A71**, 3-8.
2. SHELXL: Sheldrick, G.M. (2015). *Acta Cryst.* **C71**, 3-8.
3. Parsons,S., Flack, H.D. and Wagner, T. (2013) *Acta Cryst.* **B69**, 249-259.

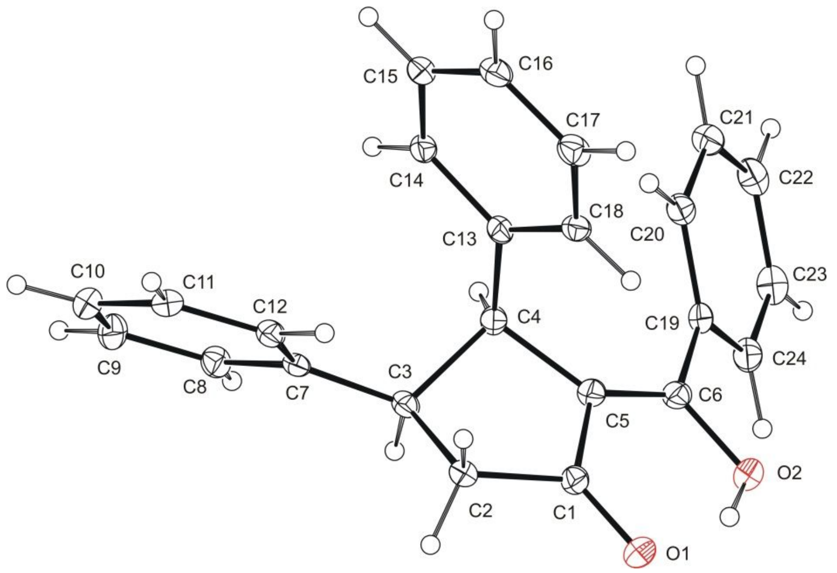

Fig.1. View on molecule of **2a**, displaying *R,S* configuration on chiral carbons C3, C4. The displacement ellipsoids at 30% probability level.

Table S1. Summary of crystallographic data and structure refinement parameters for **2a**.

Compound	**2a**
Formula	$C_{24}H_{20}O_2$
M	340.40
Crystal system	monoclinic
Space group	*P 21*
T/K	120(2)
a/Å	8.5316(6)
b/Å	9.4812(7)
c/Å	10.8081(8)
α/°	90
β/°	92.639(2)
γ/°	90
V/Å^3	873.34(11)
Z	2
μ(Mo Kα)/mm^{-1}	0.636
Diffrns collected	17714
Independent diffrns	5805
Observed[b] diffrns	5715
R_{int}^{c}/%	0.0471
No. of parameters	237
R^c obsd diffrns/%	0.0427
R, wR^c all data/%	0.0447, 0.1421
Δρ/e Å^{-3}	0.318, -0.209
CCDC entry	2063174

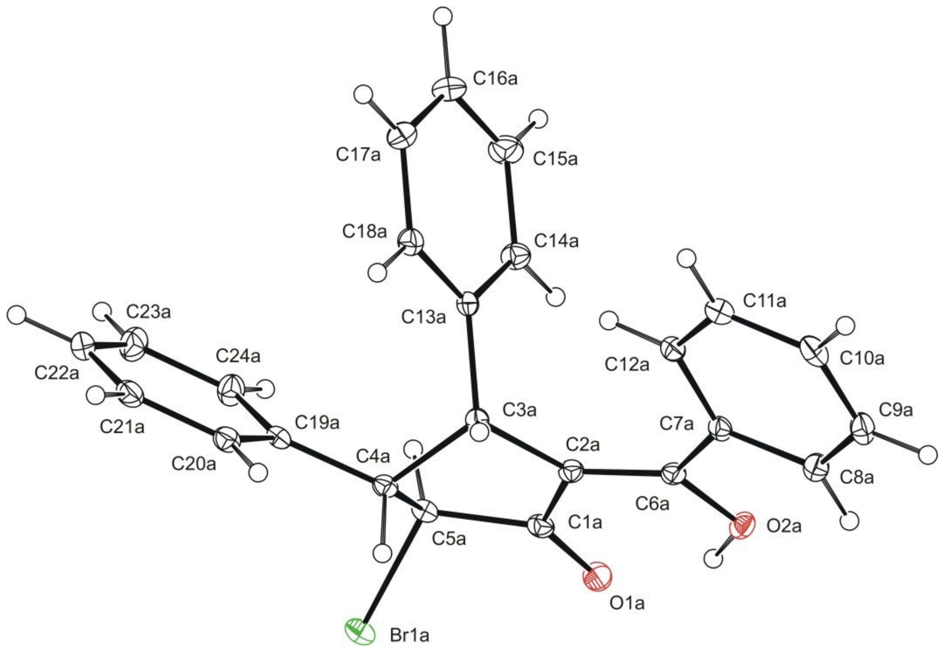

Fig.2. View on **4** displaying one of racemic pair of molecules in the crystal. The displacement ellipsoids at 30% probability level.

Table S2. Summary of crystallographic data and structure refinement parameters for **4**.

Compound	**4**
Formula	$C_{24}H_{19}BrO_2$
M	419.30
Crystal system	monoclinic
Space group	*P 21/n*
T/K	120(2)
a/Å	10.5560(5)
b/Å	9.7162(5)
c/Å	37.1846(17)
α/°	90
β/°	97.3050(10)
γ/°	90
V/$Å^3$	3782.9(3)
Z	8
μ(Mo Kα)/mm^{-1}	3.083
Diffrns collected	29899
Independent diffrns	7346
Observed[b] diffrns	7283
$R_{int}{}^{c}$/%	0.0248
No. of parameters	487
R^c obsd diffrns/%	0.0248
R, wR^c all data/%	0.0251, 0.0603
Δρ/e $Å^{-3}$	0.331, -0.410
CCDC entry	2063176

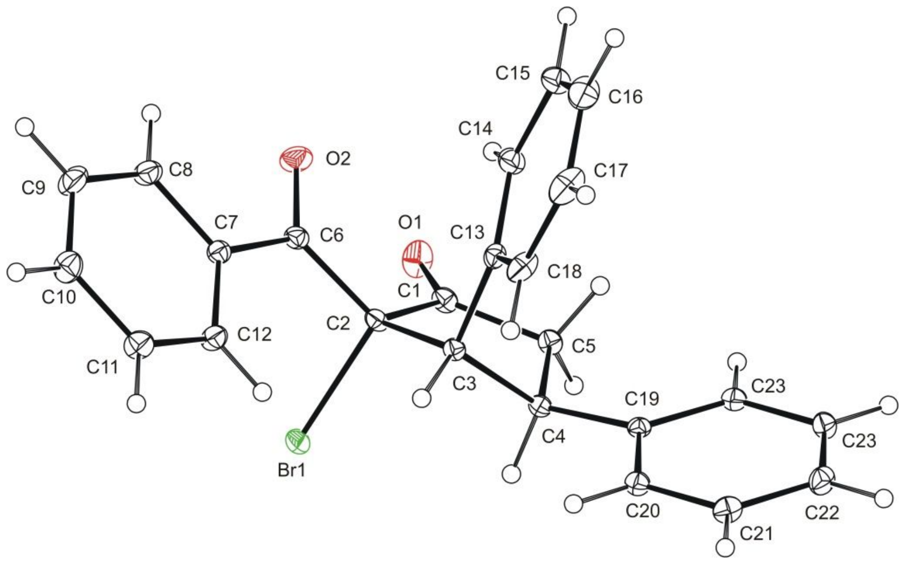

Fig.3. View on **5** displaying one of racemic pair of molecules in the crystal. The displacement ellipsoids at 30% probability level.

Table S3. Summary of crystallographic data and structure refinement parameters for **5**.

Compound	**5**
Formula	$C_{24}H_{19}BrO_2$
M	419.30
Crystal system	monoclinic
Space group	*C 2/c*
T/K	120(2)
a/Å	34.0549(12)
b/Å	9.4007(4)
c/Å	11.9196(4)
α/°	90
β/°	96.004(2)
γ/°	90
V/Å^3	3795.0(2)
Z	8
μ(Mo Kα)/mm^{-1}	2.182
Diffrns collected	22120
Independent diffrns	4349
Observed^b diffrns	4016
$R_{int}{}^c$/%	0.0231
No. of parameters	245
R^c obsd diffrns/%	0.0230
R, wR^c all data/%	0.0261, 0.0594
Δρ/e Å^{-3}	1.014, -0.281
CCDC entry	2063175
